# Construction of Dynamic Hydrogel Inducing Effective and Selective 5-Fluorouracil Monotherapy against Cervical Cancer Cells

**DOI:** 10.1021/acsami.4c20276

**Published:** 2025-02-21

**Authors:** Monika Gosecka, Daria Jaworska-Krych, Mateusz Gosecki, Malgorzata Urbaniak, Ewelina Wielgus, Bartlomiej Gostynski, Monika Marcinkowska, Anna Janaszewska, Barbara Klajnert-Maculewicz

**Affiliations:** † Centre of Molecular and Macromolecular Studies, Polish Academy of Sciences. Sienkiewicza 112, Lodz 90-363, Poland; ‡ Department of General Biophysics, Faculty of Biology and Environmental Protection, University of Lodz, 141/143 Pomorska Street, Lodz 90-236, Poland

**Keywords:** self-healable, injectable hydrogel, topical therapy, selective chemotherapy, selective anticervical cancer therapy, 5-fluorouracil, induced anticancer activity

## Abstract

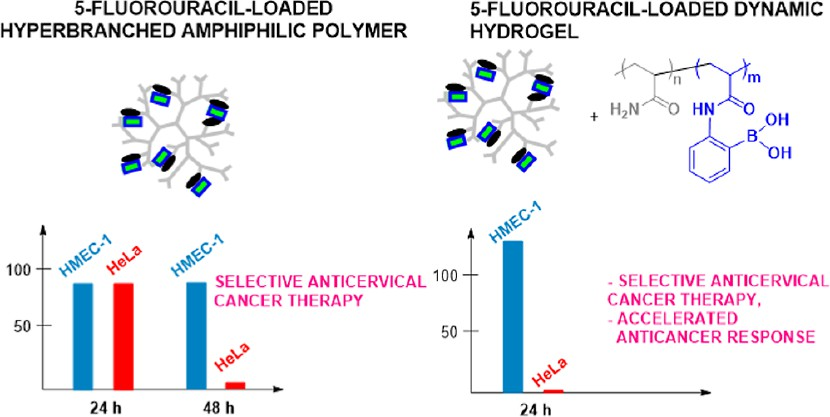

The severe side effects of systemic chemotherapy for cervical cancer encourage the use of topical intravaginal drug delivery systems. 5-fluorouracil, 5-FU, is an anticancer drug accepted in clinical use in the cancer therapy of colorectal, gastric, and hepatocellular carcinoma. However, it shows low activity against cervical cancer cells (HeLa) and thus requires the usage of additional drugs to support the therapy, which is associated with side effects. We report on the polyglycidol/polyacrylamide-based hydrogel carrier providing effective monotherapy against cervical cancer cells, HeLa with 5-FU, along with a neutral effect on normal cells, HMEC-1. The use of hyperbranched polyglycidol modified with aryl groups, i.e., phenylurethane, 1,4-biphenylurethane, and benzoyl ester, respectively, to enhance the solubility of 5-FU in the aqueous medium ensured the drug’s efficacy and selectivity against Hela cells after 48 h at a low dose. Crucially, the cross-linking of drug-loaded aryl-enriched polyglycidol with an acrylamide copolymer bearing 2-acrylamidephenylboronic acid induced the anticervical cancer activity reducing the time required for complete cervical cancer cell death to 24 h. An in vitro study showed that boronic acid moieties are responsible for the promotion of anticervical cancer activity with 5-FU. Reported hydrogels’ structure based on reversibly cross-linked aryl-enriched HbPGLs provides the self-healing properties of the network crucial for the formation of the continuous layer of formulation ensuring the delivery of the drug to the afflicted area of the covered tissue. Among tested hydrogels, the system constructed from HbPGL which linear constitutional units were modified with benzoyl ester or and phenylurethane moieties at approximately 45 mol % showed the highest drug permeability through the STRAT-M model membrane. This study demonstrates the direction of the synthetic design of the hydrogel carrier of 5-FU assuring safe monotherapy of cervical cancer cells, avoiding side effects typical for combinatory therapies.

## 1 Introduction

Cervical cancer is the third most prevalent cancer in women globally and is especially dominant in developing countries. Generally, the treatment of cervical cancer involves systemic chemotherapy, which is associated with many side effects. In addition, low stability of drug(s) in the organism results in low-drug concentration in tumor tissue and thus reduced overall efficacy. Then, high-dose systemic chemotherapy is applied, which results in severe nonspecific toxicity. To reduce the systemic effects, improve the overall patient’s quality, and overcome rapid drug metabolization, topical therapy, i.e., localized delivery of the therapeutic agents to the afflicted site, is of increasing great interest. Such an approach delivers better control of the onsite drug concentration and ensures improvement of the overall patient’s quality of life. Direct delivery of the active compounds to the tissue facilitates the use of a lower dose having to be required. Localized therapy can be used to reduce the size of tumor or can be used after the surgery to reduce the risk of recurrence. For this purpose, it is necessary to ensure prolonged contact of drug molecules with the cervix, i.e., extended exposure of the cancer cells to a therapeutic agent.

For topical vaginal therapies, there is growing interest in hydrogels, i.e., polymer networks with water as a continuous phase) as advanced therapeutic platforms that replace standard vaginal globules and tablets by preventing uncontrolled leakage of drug formulation contents due to their high viscosity provided by the cross-linked structure. Localized chemotherapy with the use of hydrogel-based carriers of bioactive compounds is problematic as many anticancer drugs are poorly soluble in water. The challenge is to ensure the solubility of drugs in the aqueous medium and thus increase their bioavailability. The limited solubility of drugs in aqueous media results mainly from their hydrophobic nature. Then, hydrophobized hydrogels are designed to ensure the solubility of the bioactive compound.^1−4^ However, limited water solubility can also occur with hydrophilic drugs. As exemplary, 5-fluorouracil (5-FU), a pyrimidine analogue, is a hydrophilic drug of anticancer activity that is poorly soluble in water. The difficulty in its solubility in water, even under physiological conditions, is associated with the presence of groups containing strong hydrogen bond donors (i.e., NH) and acceptors (i.e., –CO) prone to self-assembling in the form of fibrils. The phenomenon of fibril formation is a reason for its reduced effectiveness in anticancer activity.^5^*N*-methylated derivatives of 5-FU such as capecitabine and gemcitabine are water-soluble since they are not prone to fibril formation and thus are more effective in cancer treatment.

5-FU was discovered in the 1950s by Heidelberger who added a fluorine atom in the fifth position to the methyl group of uracil creating a compound with high anticancer potential.^6^ Clinical trials conducted in the 1960s and 1970s demonstrated the efficacy of this drug against colorectal cancer.^7,8^ 5-FU has been approved in clinical practice for systemic chemotherapy of colorectal cancer, gastric cancer, and hepatocellular carcinoma. 5-FU is an antimetabolite drug that exerts two mechanisms of anticancer activity, i.e., the inhibition of thymidylate synthase enzyme and misincorporation into DNA and RNA.^9^ 5-FU is converted intracellularly to several active metabolites, i.e., fluorodeoxyuridine monophosphate (FdUMP), fluorodeoxyuridine triphosphate (FdUTP), and fluorouridine triphosphate, which disrupt RNA synthesis and the action of thymidylate synthase.^9^ The administration of 5-FU by systemic route results in a short plasma half-life (11.4 min after intravenous administration) and drug clearance from plasma within 1 h as a consequence of very rapid metabolism by the dihydropyrimidine dehydrogenase or uracil reductase enzymes^10^ 80% of administered 5-FU is normally catabolized, primarily in the liver. This implies low-drug concentration in tumor tissue and reduced overall efficacy. For this reason, high doses of chemotherapeutic drugs need to be used. The efficacy of 5-FU is increased by combination therapy with the usage of additional chemotherapeutics to increase its anticancer activity (response rates were raised to 40–50%).^11,12^ The treatment of cervical cancer with 5-FU has not been approved due to its poor activity. Researchers are focused on the improvement of its activity by using the combination therapy leading to a significant therapeutic synergism with such drugs as cisplatin,^13^ imiquimod,^14^ and norcantharidin.^12,15^ The necessity of the use of additional bioactive compounds is associated with harmful effects.

Limitations arising from the systemic delivery of 5-FU, affecting its reduced effectiveness, enforce the use of topical anticancer therapy with 5-FU. There is a need for a hydrogel-based drug carrier that would ensure maintaining 5-FU structural stability, increase its bioavailability, and promote the gradual release of the drug. To date, hydrogel carriers of 5-FU with sustained release of the active compound have been investigated for the treatment of breast,^16^ colorectal,^12,17,18^ gastric,^19^ and cervical^20^ cancers using 5-FU alone or in combination with another active compound.^17,18,21^ A composite hydrogel^19^ or thermosensitive hydrogel formation based on micelle formation strategy^17^ was used to construct hydrogels to achieve solubilization of 5-FU in an aqueous environment. Synergistic therapeutic activity against cervical cancer cells has been achieved for the hydrogel carrier of 5-FU combined with CuO nanoparticles. However, to date, there are no data on a hydrogel carrier of 5-FU for the therapy of anticervical cancer that provides rapid, effective, and selective antitumor activity, along with appropriate rheological properties of the carrier, such as injectability, which is of great importance in view of the site of administration of the formulation.

In this article, we present the strategy of a significant increase of 5-FU efficacy in a monotherapy against cervical cancer cells along with a negligible effect on the normal cells by proper adjustment of the hydrogel carrier’s building components. The hydrogel systems have been constructed of aryl-modified hyperbranched polyglycidol with an acrylamide copolymer bearing 2-acrylamidephenylboronic acid (2-AAPBA) moieties. Aryl-enriched hyperbranched polyglycidols were responsible for the enhancement of anticancer activity by the enhancement of 5-FU solubility in the aqueous media, whereas boronic acid motifs, besides cross-linking of polyglycidol macromolecules, unexpectedly induced the selective anticervical cancer activity. The investigations were performed by using dermal microvascular endothelium cells (HMEC-1) and human cervical cancer endothelial cells (HeLa). We revealed the strict relationship between the molecular structure of building components of the drug carrier, the bioavailability of loaded 5-FU, and its biological response. According to our knowledge, this work presents a strategy for the formation of a hydrogel platform of 5-FU, which ensures both selective and efficient anticervical cancer therapy and may represent a breakthrough in the field of anticervical cancer therapy.

## 2 Experimental Section

### 2.1 Materials

Glycidol and 1,1,1-tris(hydroxymethyl)propane were purchased from Sigma-Aldrich. 2,2-dimethoxypropane, benzoyl chloride, and phenyl isocyanate were purchased from Alfa Aesar. Anhydrous pyridine was purchased from Acros-Organics. *p*-toluene sulfonic acid (PTSA) (Sigma-Aldrich) was dried with benzene. Glycidol was dried with 4 Å molecular sieves and distilled under reduced pressure.

### 2.2 Synthesis of Hyperbranched Polyglycidol

**Scheme 1:**
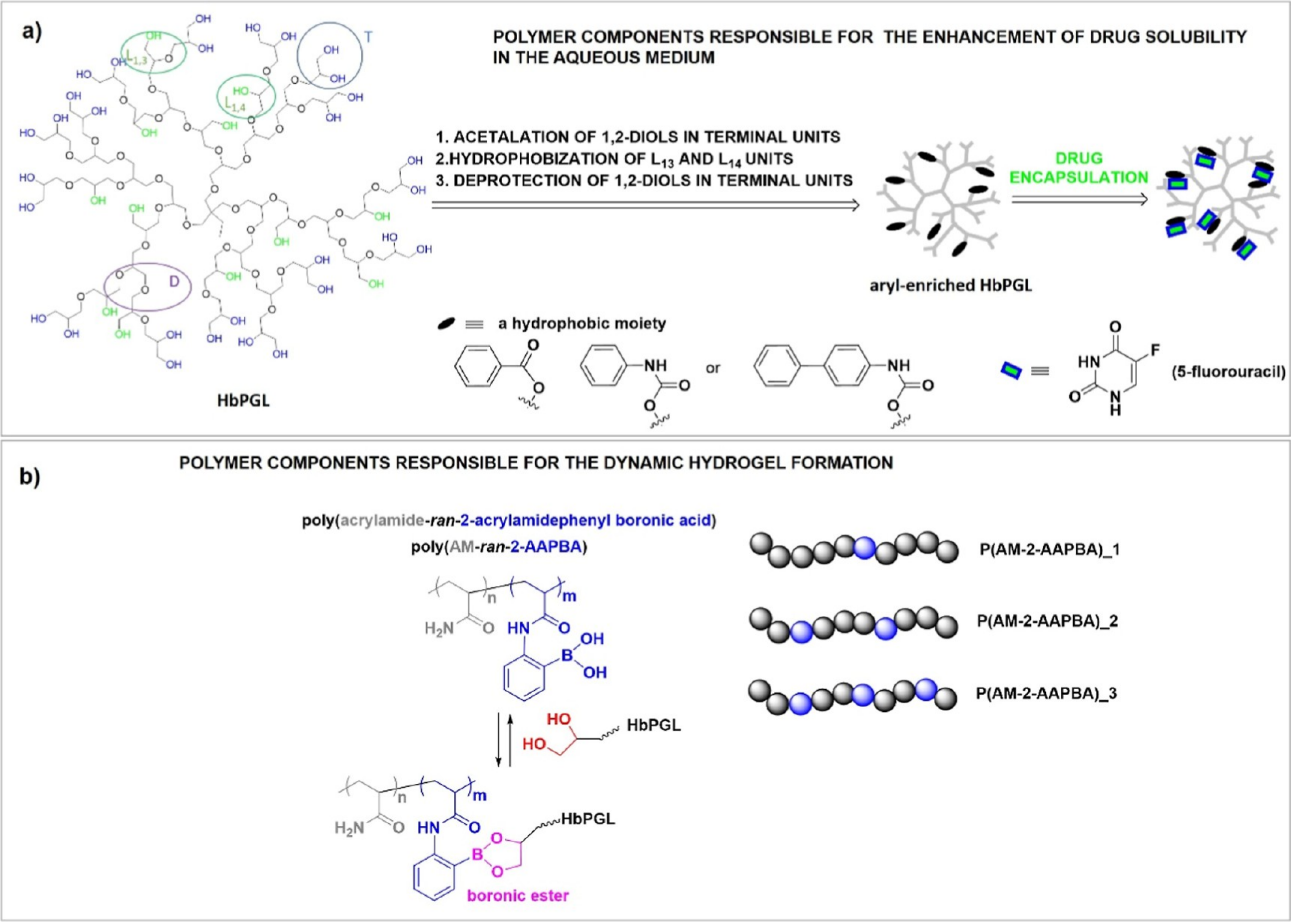
(a) Schematic Representation of Constitutional Repeating Units of Hyperbranched Polyglycidol and Selective Modification of Linear L_1,3_ and L_1,4_ units with Aromatic Groups Incorporated via Ester or Urethane Linkages along with the Loading of 5-Fluorouracil within Amphiphilic constructs (b) The structure of acrylamide copolymers with 2-acrylamidephenylboronic acid [poly(AM–*ran*–2–AAPBA)] prone to cross-linking with 1,2-diols of hyperbranched polyglycidol present in terminal T constitutional repeating units.

Hyperbranched polyglycidol was obtained according to the procedure described previously. The structure of the synthesized polymer was characterized by ^13^C INVGATE and DEPT NMR spectroscopies (Figures S1 and S2). The degree of branching of synthesized neat HbPGL was 0.55. The molar fraction of dendritic (D) and total linear constitutional units L_13_ and L_14_ bearing monohydroxyl groups was 0.26 and 0.41, respectively, whereas the molar fraction of terminal units (T) containing diol moieties was 0.33. D, L_13_, L_14_, and T units are denoted in Scheme 1. The weight-average molecular mass *M*_w_ was determined based on GPC results using water as an eluent and is equal to 12,000 g/mol, while the dispersity *D̵* = 1.8 (Figure S3).

^13^C INVGATE NMR (400 MHz, DMSO) δ (ppm): 80.54 (CH-L_1,3_), 78.64 (CH-D), 73.29 (CH_2_-2L_1,4_), 72.00-71.18 (CH_2_-2D, 2T), 69.91 (CH_2_-L_13_), 69.31 (CH-L_1,4_), 63.51 (CH_2_-T), 61.43 (CH_2_-L_1,3_).

### 2.3 Synthesis of HbPGL with Protected 1,2-Diols in Terminal Constitutional Repeating Units

1,2-diols of terminal constitutional units of hyperbranched polyglycidol (40 g) were chemically protected according to the procedure described previously^22^ in reaction with 2,2-dimethoxypropane (192 mL, 1.56 mol) in the presence of PTSA (0.384 g, 2.22 mmol) by ultrasonication at 40 °C for 3 h. The crude product was diluted with chloroform and extracted three times with a saturated Na_2_CO_3_ solution to remove PTSA. The organic phase was dried over MgSO_4_ and dialyzed in chloroform for 24 h. The product was then dried under high vacuum and analyzed by ^1^H NMR, ^13^C INVGATE, and DEPT NMR spectroscopies in deuterated DMSO to confirm the conversion of terminal 1,2-diol groups to acetals (acetal), Figures S4–S6.

^1^H NMR (400 MHz, DMSO) δ (ppm): 4.84-4.36 (OH-L_13_, L_14_); 4.14 m, 1H, C*H*-PG (T_Acetal_); 3.97 (m, 1H, C*H*(H)-PG (T_Acetal_); 3.80-3.20 (5H-HbPGL backbone, m, 1H, CH(*H*)-PG (T_Acetal_)); 1.30 (3H-CH_3_ T_Acetal_), 1.26 (3H-CH_3_ T_Acetal_)

^13^C INVGATE NMR (400 MHz, DMSO) δ (ppm): 80.65 (CH-L_1,3_), 78.37 (CH-D), 74.70 (CH-T_Acetal_), 73.37 (CH_2_-2L_1,4_), 72.33-71.17(CH_2_-2D, T_Acetal_), 69.94 (CH_2_-L_13_), 68.99 (CH-L_1,4_), 66.51 (CH_2_-T_Acetal_), 61.32 (CH_2_-L_1,3_), 27.10 (CH_3_-T_Acetal_), 25.83 (CH_3_-T_Acetal_).

### 2.4 Hydrophobization of HbPGL Core with Phenylurethane or 1,4-Biphenylurethane Moieties

Hyperbranched polyglycidol with protected 1,2-diol groups (5 g) in terminal units was dried with benzene, dissolved in pyridine (30 mL) under argon conditions, and heated to 50 °C. Then, 4-biphenyl isocyanate solution in pyridine (0.106 g/mL) or 0.63 or 0.93 mL of phenyl isocyanate, respectively, was dropped. The reaction was carried out for 24 h. The reaction mixture was dialyzed against DMSO. After evaporation of the solvent, the degree of hydrophobization of all monohydroxyl units was determined based on the ^1^H NMR spectrum recorded in deuterated DMSO-*d*_6_, comparing the integration of methyl protons from acetal groups in terminal units (1.15 and 1.35 ppm) and aromatic protons from biphenyl groups (7.10 to 7.75 ppm). In the case of the phenyl-HbPGL derivatives, 30 or 42 mol % of all L_13_ and L_14_ units were hydrophobized, i.e., 19.5 or 27.8 hydrophobized units per macromolecule, respectively (Figures S7 and S8) while for the biphenyl-enriched HbPGL derivative, 40 mol % of all L_13_ and L_14_ units were hydrophobized, i.e., 25.6 hydrophobic units per macromolecule (Figure S9).

^1^H NMR (400 MHz, DMSO-*d*_6_) δ (ppm) for the PC-HbPGL derivative: 9.78-9.55 (1H-NH); 7.46-2H, 7.25-2H, 6.97-1H-phenyl); 4.97 (1H-CH- L_1,4-hydrophobized_); 4.86-4.38 (1H-OH-L_1,3_, L_1,4_); 4.20 (1H-CH_2_-L_1,3-hydrophobized_); 4.11 (1H-CH_2_-T_Acetal_); 3.94 (1H-CH_2_-T_Acetal_); 3.82-3.02 (HbPGL-backbone); 1.29 (3H-CH_3_ T_Acetal_); 1.23 (3H-CH_3_ T_Acetal_).

^1^H NMR (400 MHz, DMSO-*d*_6_) δ (ppm) for the BPh-HbPGL derivative: 9.97-9.68 (1H-NH); 7.57 (6H-biphenyl); 7.40 (2H-biphenyl); 7.30 (1H-biphenyl); 5.01 (1H-CH- L_1,4-hydrophobized_); 4.89-4.44 (1H-OH-L_1,3_, L_1,4_); 4.24 (1H-CH_2_-L_1,3-hydrophobized_); 4.12 (1H-CH_2_-T_Acetal_); 3.94 (1H-CH_2_-T_Acetal_); 3.82-3.02 (HbPGL-backbone); 1.29 (3H-CH_3_ T_Acetal_); 1.23 (3H-CH_3_ T_Acetal_).

Subsequently, 1,2-diol groups of the polymer in terminal units were deprotected by the addition of an aqueous solution of 0.1 M HCl to the polymer solution in DMSO and stirred overnight at room temperature. The mixture was dialyzed against deionized water for 24 h, changing the solvent until a neutral pH. The product was lyophilized and characterized by using ^1^H NMR spectroscopy in deuterated pyridine (Figures S11–S13) and ^1^H DOSY NMR spectra (Figures S15–S17).

^1^H NMR (400 MHz, Py-*d*_5_) δ (ppm) for PC-HbPGL derivative: 11-10.4-1H-NH; 7.91-2H, 7.36-2H, 7.05-1H-phenyl; 5.48-1H-CH- L_1,4-hydrophobized_; 4.58-1H-CH_2_-L_1,3-hydrophobized_; 4.50-1H-CH_2_-L_1,3-hydrophobized_; 4.42-3.07-HbPGL-backbone.

^1^H NMR (400 MHz, Py-*d*_5_) δ (ppm) for BPh-HbPGL derivative: 11.10-10.5-1H-NH; 7.99-2H, 7.67-4H, 7.41-7.38-2H, 7.29-1H-biphenyl; 5.51-1H-CH-L_1,4-hydrophobized_; 4.60-1H-CH_2_-L_1,3-hydrophobized_; 4.50-1H-CH_2_-L_1,3-hydrophobized_; 4.41-3.00-HbPGL-backbone.

### 2.5 Hydrophobization of AC-HbPGL Core with Benzoate Groups (Ester Derivative)

Hyperbranched polyglycidol with protected 1,2-diol groups (5 g) in terminal units was dried with benzene, dissolved in pyridine (30 mL) under argon conditions, and cooled in an ice-bath to 0 °C. Then, 0.81 mL of benzoyl chloride was dropped. The reaction was carried out for 24 h. The reaction mixture was dialyzed against DMSO. After evaporation of the solvent, the degree of hydrophobization of all monohydroxylated units was based on the ^1^H NMR spectrum recorded in deuterated DMSO-*d*_6_, comparing the integration of methyl protons from acetal groups in terminal units (1.15 and 1.35 ppm) and aromatic protons from phenyl groups (7.21 to 8.02 ppm) (Figure S10). The degree of the substitution of L_13_ and L_14_ units was equal to 45 mol %.

^1^H NMR (400 MHz, DMSO-*d*_6_) δ (ppm) for the BE-HbPGL derivative: 7.95-2H, 7.62-1H, 7.50-2H (phenyl); 5.25 (1H-CH-L_1,4-hydrophobized_); 5.01-4.31 (1H-OH-L_1,3_, L_1,4_); 4.24 (1H-CH_2_-L_1,3-hydrophobized_); 4.13 (1H-CH_2_-T_Acetal_); 3.94 (1H-CH_2_-T_Acetal_); 3.82-3.14 (HbPGl-backbone); 1.29 (3H-CH_3_ T_Acetal_); 1.25 (3H-CH_3_ T_Acetal_).

After the determination of the hydrophobization degree, the diol groups were deprotected by adding a 0.1 M HCl aqueous solution to the polymer solution in DMSO and stirred overnight. Finally, the mixture was dialyzed against deionized water and analyzed with ^1^H NMR spectroscopy in deuterated pyridine (Figure S14) and ^1^H DOSY NMR spectroscopy (Figure S18).

^1^H NMR (400 MHz, Py-*d*_5_) δ (ppm) for the BE-HbPGL derivative: 8.21-2H, 7.47-1H, 7.38-2H (phenyl); 5.72-1H-CH-L_1,4-hydrophobized_; 4.75-1H-CH_2_-L_1,3-hydrophobized_; 4.6-1H-CH_2_-L_1,3-hydrophobized_; 4.46-2.58-HbPGL-backbone.

### 2.6 Synthesis of Poly(acrylamide-*ran*-2-acrylamidephenylboronic acid) [Poly(AM–*ran*–2–AAPBA)]

A series of acrylamide copolymers with 2-acrylamidephenylboronic acid, 2-AAPBA have been synthesized using a conventional radical copolymerization of acrylamide and 2-AAPBA pinacol ester initiated with AIBN. The molar ratio of acrylamide to 2-AAPBA was as follows: 90:10; 80:20; and 70:30, respectively. The initial molar ratio of comonomers to AIBN was 220:1. The copolymerization reactions were carried out in 15 mL of a DMF/dioxane mixture (5:1 v/v) at 70 °C for 16 h. Each copolymerization mixture was diluted in water, and each copolymer was precipitated in acetone and dried. Next, each copolymer was dissolved in an alkaline solution of NaOH (1 wt %) and dialyzed against deionized water using a 1000 MW cutoff dialysis membrane, at first against the alkaline aqueous solution and then against water, which was changed several times to reach the neutral pH. Dialysis was necessary to hydrolyze pinacol boronic ester units and remove released pinacol. ^1^H NMR spectra recorded in DMSO-*d*_6_ were used to determine the molar composition of the synthesized copolymers (Figures S19–S21), whereas ^1^H DOSY NMR spectroscopy (Figures S22–S24) was used to confirm the copolymer structure. GPC chromatograms are presented in Figures S25–S27. The structural characteristics of synthesized AM-2-AAPBA copolymers are given in Table 1.

**Table 1:** Characteristics of Acrylamide-2-acrylamidephenylboronic Acid Copolymers Obtained via Uncontrolled Radical Copolymerization

copolymer	theoretical molar ratio of AM–2–AAPBA	experimental molar ratio of AM–2–AAPBA	*M*_*n*_	*D̵*
P(AM–2–AAPBA)_1	90:10	90.8:9.2	54300	1.7
P(AM–2–AAPBA)_2	80:20	82.4:17.6	31000	1.9
P(AM–2–AAPBA)_3	70:30	78.4:21.6	27000	2.2

^1^H NMR (400 MHz, DMSO-*d*_6_) δ (ppm) for the poly(AM–*ran*–2–AAPBA): 7.65-6.50 (4H, aromatic), 2.74-1.00 (2H, CH_2_, 1H, CH, backbone).

### 2.7 Solubilization of 5-Fluorouracil within Aryl-Enriched Hyperbranched Polyglycidol

A stock solution of 5-FU (5 mg/mL) in methanol was prepared. A total of 75 mg of each internal hydrophobized polyglycidol was dissolved in 3 mL of methanol. A series of drug and polymer mixtures in methanol were prepared in the following molar ratio of 5-FU to HbPGL: 5:1; 10:1; and 20:1. Each mixture was stirred for 6 h. Subsequently, methanol was allowed to evaporate at 37 °C.

### 2.8 Solubilization of 5-Fluorouracil within P(AM–*ran*–2–AAPBA) Copolymers

A total of 10 mg of each P(AM–*ran*–2–AAPBA) copolymer was dissolved in the mixture composed of 2 mL of methanol and 2 mL of water. 5-FU solution was added to the copolymer solution at a molar ratio of 5-FU to boronic acid equal to 0.6:1. The mixture was stirred for 6 h. Subsequently, the solvents were allowed to evaporate at 37 °C overnight.

### 2.9 Hydrogel Formation

Hydrogel samples were prepared using the constant weight fraction of both aryl-enriched hyperbranched polyglycidol and P(AM–*ran*–2–AAPBA)_1 by mixing 0.08 mL of aqueous solution containing 0.019 g of poly(AM–*ran*–2–AAPBA) with 0.05 mL of aqueous solution containing 0.021 g of each drug-loaded HbPGL derivative.

### 2.10 NMR Spectroscopy

All ^1^H NMR spectra were carried out at 295 K on a Bruker Avance NEO 400 spectrometer. The structure of polymers was confirmed based on spectra registered in DMSO-*d*_6_ or pyridine-*d*_5_.

### 2.11 Cell Culture

Dermal microvascular endothelium cells (HMEC-1) and human cervical cancer endothelial (HeLa) cells were grown according to the procedure described previously.^4^

### 2.12 Determination of Cytotoxicity

The effects of the aryl-enriched hyperbranched polyglycidols and 5-FU-loaded hyperbranched polyglycidols, and a free drug on the cell viability, were determined using the (3-[4,5-dimethylthiazol-2-yl]-2,5 diphenyl tetrazolium bromide) (MTT) assay.

To the 96-well plates containing cells at a density of 1.5 × 10^4^ cells/well in an appropriate medium were added different concentrations of all compounds. Then, cells were incubated with aryl-enriched hyperbranched polyglycidols in 1–300 μM 5-FU concentration range (1; 10; 50; 100; and 300 μM) for 24 and 48 h in a 37 °C humidified atmosphere containing 5% CO_2_. After the incubation time, cells were washed with 50 μL of phosphate-buffered saline (PBS). The cells were then incubated for 3 h under normal culture conditions after the addition of 50 μL of a 0.5 mg/mL MTT solution in PBS to each well. After incubation, the remaining MTT solution was removed and the resulting formazan precipitate was dissolved in DMSO (100 μL/well). The conversion of the tetrazolium salt (MTT) to a colored formazan by mitochondrial and cytosolic dehydrogenases is a marker of cell viability. Before absorbance measurements, plates were vortexed for 1 min, and the absorbance at 570 nm was measured using a PowerWave HT Microplate Spectrophotometer (BioTek).

The determinations were carried out in two biological replicates and eight technical replicates.

#### 2.12.1 Microscopic Images

HMEC-1 and HeLa cells were suspended in a culture medium at 500,000 and 400,000 cells/mL, respectively, and seeded outside the cell culture insert (ibidi, Munich, Germany. www.ibidi.de). After 24 h (after ensuring that the cells covered the entire free surface outside the inset), a 30 μL volume of the respective gel (pure hydrogel, hydrogel with 5-FU was pipetted into each chamber of the ibidi insert. The volume of 30 μL gel was constructed of drug-loaded aryl-enriched hyperbranched polyglycidols in which 5/10/20 mol 5-FU, respectively, were encapsulated per mole of each polymer. The ibidi inserts were removed, and images were taken immediately and after 24 h of incubation with gels, at 4× magnification, using a Nikon ECLIPSE E200 microscope and ImageJ software (NIH, Rockville).

### 2.13 Computational Details

The initial structures of 5-FU nucleotide with boronic acid were optimized with the semiempirical GFN2-xTB^23^ method via xTB 6.7.1 software^24^ in collaboration with the Orca 6.0 program.^25^ The resulting structures subsequently became input geometries to the conformational search performed with Crest 3.0.2 software.^26^ The best conformer was then optimized using a Gaussian16 set of codes^27^ with ωb97XD/6-311 + g(d) method; solvent (water) was modeled as a continuum-solvation CPCM approximation. Various initial structures of the noncovalently bound complexes created using the optimized substrate structures were treated with the same workflow, and their energetically lowest conformer was taken to final calculations of the free energy change (Δ*G*) of their complexation reactions.

### 2.14 Rheology

The oscillation frequency sweep tests were performed on a Thermoscientific HAAKE MARS 40 rheometer in the linear viscoelastic regime using a parallel plate–plate geometry of an 8 mm diameter with a 0.3 mm gap at 25 and 37 °C. The self-healing tests for all investigated hydrogels were performed in the oscillation time mode (1 Hz) at 37 °C by monitoring both storage and loss moduli. The hydrogel sample was first placed under a 1% strain for 180 s, and then it was destroyed with a 5 s 300% strain pulse, after which 1% was again applied for sample restoration.

### 2.15 Franz Cell

For the study, a piece of the hydrogel sample constructed of drug-loaded HbPGL derivative cross-linked with poly(AM–*ran*–2–AAPBA) was placed on a Strat-M membrane into the donor compartment of the Franz cell.

For comparison, an aqueous suspension of 5-FU was prepared in the simulated vaginal fluid at the same drug concentrations, which was investigated in the hydrogel systems.

The receptor compartment was filled with 12.5 mL of the simulated vaginal fluid at 37 ± 1 °C. Aliquots of 0.5 mL of the receptor solution were taken at different time intervals for 168 h. Each withdrawn aliquot was immediately replaced with the same volume of the corresponding fresh portion of the simulated vaginal fluid.

The concentrations of 5-FU were determined by an ACQUITY UPLC I-Class chromatography system coupled with a SYNAPT G2-Si mass spectrometer equipped with an electrospray source and a quadrupole-time-of-flight mass analyzer (Waters Corp., Milford, MA). An Acquity BEHTM C18 column (100 × 2.1 mm, 1.7 μm) maintained at 45 °C temperature was used for the chromatographic separation of an analyte. The mobile phase was prepared by mixing 0.1% formic acid (A) and 0.1% formic acid in acetonitrile (B). The elution gradient was: 32% B (0–1.0 min), 32–95% B (1.0–3.0 min), 95–95% B (3.0–3.5 min), 95–32% B (3.5%–3.52 min), and 32–32% B (3.52–7.0 min). The flow rate was 0.45 mL/min, and the injection volume was 0.5 μL for 5-FU.

For mass spectrometric detection, the electrospray source was operated in a positive resolution mode using a capillary voltage of 3.0 kV, a cone voltage of 20 V, a desolvation gas flow of 400 L/h, a temperature of 350 °C, a nebulizer gas pressure of 6.5 bar, and a temperature of the source equal to 100 °C. The mass spectra were recorded in the *m*/*z* range from 100 to 1200. The system was controlled by MassLynx software (Version 4.1), and data processing was performed by the TargetLynxTM program.

The initial stock calibration solutions of 5-FU were created in the simulated vaginal fluid. The stock solutions were serially diluted with 0.5 mL of the simulated vaginal fluid and 0.5 mL of deionized water to obtain working solutions at several concentration levels. The calibration curves were prepared at 10 different concentrations of 5-FU solutions and were linear over a concentration range from 0.709 to 11.390 μg/mL with a correlation coefficient of >0.995.

## 3 Results and Discussion

### 3.1 Selection of Polymer Components for the Construction of the Hydrophobized Hydrogel-Based Carrier of 5-Fluorouracil

For the loading of poorly water-soluble 5-FU, we selected amphiphilic hyperbranched polyglycidols with a core enriched with different aryl moieties immobilized via ester or urethane linkages. Among incorporated aryl moieties, benzoyl ester, phenylurethane, and 1,4-biphenylurethane moieties were used (Scheme 1). The hydrophobized hyperbranched polyglycidols (hHbPGLs) were synthesized according to Scheme S1 by the selective modification of monohydroxylated linear constitutional repeating units in the inner region of hyperbranched polyglycidol, HbPGL in the reaction with benzoyl chloride, phenyl isocyanate, and 1,4-biphenyl isocyanate, respectively, whereas 1,2-diols in terminal units remained intact. Such construction of amphiphilic polyethers required the protection of 1,2-diols in terminal units of HbPGL with 2,2-dimethoxypropane prior to the modification of linear units L_13_ and L_14_ with aryl groups and then the deprotection of 1,2-diols groups. The protection of all 1,2-diol groups in terminal units was confirmed based on ^13^C INVGATE NMR spectrum based on the complete decay of carbon atom of one of methylene group in the terminal unit at 63.5 ppm typical for unprotected HbPGL along with the appearance of methylene carbon signals at 66.5, 72.3 ppm, and a signal at 74.7 ppm corresponding to methine carbon in the protected (acetylated) terminal units (Figure S5). The chemical shifts of carbon atoms in the protected terminal units were determined based on the DEPT spectrum (Figure S6).

For the study, we selected hHbPGLs in which linear units were modified with benzoyl ester, phenylurethane, and 1,4-biphenylurethane groups, respectively, at approximately 40 mol %. In addition, to evaluate the effect of a degree of hydrophobization of all linear repeating constitutional units of HbPGL, phenylurethane derivatives at the degree of modification of L_13_ and L_14_ units equal to 30 mol % (PC30) were synthesized.

The degree of substitution of linear (L_13_ and L_14_) constitutional repeating units in HbPGL was initially estimated for acetylated forms of aryl-enriched HbPGLs based on ^1^H NMR spectra (Figures S7–S10) comparing the integration of methyl protons of acetal groups and aromatic protons and known molar fractions of all linear (L_13_ and L_14_) and terminal constitutional repeating units known based on the ^13^C INVGATE NMR spectrum recorded for the neat HbPGL (Figure S1). The degree of substitution of final products, i.e., after the deprotection step of 1,2-diol units of terminal units, was determined based on the spectra recorded for all amphiphilic HbPGL derivatives in pyridine-*d*_5_ (Figures S11–S14) comparing the integration of aromatic protons with the integration of CH and CH_2_ protons of the HbPGL backbone coming from all repeating units (D, T, L_13_, L_14_, hydrophobized L_13_, and hydrophobized L_14_), relative to the molar fraction of all linear constitutional repeating units (L_13_, L_14_) known based on the ^13^C INVGATE NMR spectrum recorded for the neat HbPGL (Figure S1). In addition, ^1^H DOSY NMR spectra (Figures S15–S18) revealed comparable values of diffusion coefficients of protons of both aryl groups and polyether backbone (at around 3.5 ppm).

Hydrophobized hyperbranched polyglycidols, thanks to the presence of 1,2-diol groups in the corona, were applied to the formation of hydrogel-based drug carriers constructed of dynamic cross-links based on boronic esters. For this purpose, acrylamide (AM) copolymers with 2-AAPBA, characterized by different molar ratios of AM to 2-AAPBA were synthesized via uncontrolled radical copolymerization. The molar ratio of both comonomers was determined based on ^1^H NMR spectra recorded in D_2_O (Figure S19–S21). ^1^H DOSY NMR spectra (Figure S22–S24) confirmed the structure of AM-2AAPBA copolymers as values of the diffusion coefficients of both aliphatic backbone and aromatic protons were comparable, in spite of the initial composition of copolymerization mixtures.

## 4 5-Fluorouracil Loading within Aryl-Enriched Hyperbranched Polyglycidols

HbPGLs with an aryl-enriched core modified via ester or urethane linkages were applied to load 5-FU. The low solubility of 5-FU in water results in the formation of the fibrils and thus low bioavailability required to adjust the environment for the structure of drug molecules. We established that the presence of diverse functional groups in the structure of hHbPGL, i.e., ether, monohydroxyl, diol, ester/urethane groups, and aryl moieties, should prevent drug molecules from aggregation in the aqueous medium. The process of 5-FU loading within amphiphilic constructs was carried out according to a solvent evaporation method using methanol as a good solvent for both hHbPGL and the drug. Thanks to the usage of different molar ratios of drug molecules per macromolecule, a series of drug-loaded amphiphilic constructs were obtained (Table 2). Such an approach should facilitate pointing out the most promising system for the formation of hydrogel-based drug delivery systems.

**Table 2:** Data of Molar Ratio of 5-Fluorouracil Loaded within Internally Hydrophobized Hyperbranched Polyglycidols Bearing Various Aromatic Moieties

aryl-enriched HbPGL derivative	sample	molar ratio of drug to macromolecule	molar ratio of aryl-modified units in HbPGL to 5-FU	molar ratio of L_13_ and L_14_ units in HbPGL to 5-FU
PC30	CAP_PC30_5	5	2	5.97
	CAP_PC30_10	10	0.95	2.98
	CAP_PC30_20	20	0.62	1.49
PC42	CAP_PC42_5	5	3.57	4.95
	CAP_PC42_10	10	1.76	2.46
	CAP_PC42_20	20	0.88	1.23
BE45	CAP_BE45_5	5	3.70	4.83
	CAP_BE45_10	10	1.85	2.41
	CAP_BE45_20	20	0.92	1.20
BPh40	CAP_BPh40_5	5	3.32	5.20
	CAP_BPh40_10	10	1.66	2.60
	CAP_BPh40_20	20	0.83	1.32

## 5 Biological Results

The potential use of selected polymer components for the construction of carriers of 5-FU designed for the therapy of cervical cancer was investigated by determination of the viability of HeLa cells giving the IC_50_ value for free drug, neat (co)polymers, and drug-loaded (co)polymers. In addition, the effect of all compounds was also analyzed on the noncancerous HMEC-1 endothelial cells. The cytotoxicity profiles performed for aryl-enriched HbPGLs show that neat polymers were nontoxic for both HeLa and HMEC-1 cell lines in the tested concentration range (Figures S28–S31), unfortunately, at the same range as the pure drug, i.e., 5-FU.

The results obtained for pure polymers are consistent with literature reports showing that structures containing HbPGL, which combine low specific viscosity with high water solubility and easy synthetic availability, are nontoxic and can be used safely in living organisms, for example as a replacement for human serum albumin.^28−30^ The half-life of HbPGL-containing structures in plasma is up to 34 h, clearly indicating their potential use as slow-release drug carriers and confirming their suitability as 5-FU delivery systems.

The 5-FU loading within all aryl-enriched hyperbranched polyglycidols resulted in the improvement of the anticancer activity of this drug (Figures S28–S31). The IC_50_ values for 24 and 48 h of incubation, grouped by aryl-type of hyperbranched polyglycidol and drug loading, are shown in Table 3.

**Table 3:** Comparison of IC_50_ Value for 5-Fluorouracil-Loaded Aryl-Enriched Hyperbranched Polyglycidols in HeLa and HMEC1 Cell Lines^a^

			HeLa		HMEC-1	
drug-loaded polymer sample	polymer	molar ratio of 5-FU aryl-enriched HbPGL	24 h	48 h	24 h	48 h
—	PC30	—	>300	>300	>300	>300
—	PC42	—	>300	>300	>300	>300
—	BE45	—	>300	>300	>300	>300
—	BPh40	—	>300	>300	>300	>300
5-FU	—	—	>300	>300	>300	>300
CAP_PC30_5	PC30	5	73.46 ± 2.54****^####^	1.13 ± 6.24****^####^	>300	87.60 ± 5.59****
CAP_PC30_10	PC30	10	>300	>300	>300	22.04 ± 4.20****^####^
CAP_PC30_20	PC30	20	>300	15.46 ± 7.67****^####^	>300	>300
CAP_PC42_5	PC42	5	>300	37.49 ± 4.42****^####^	>300	>300
CAP_PC42_10	PC42	10	>300	59.30 ± 4.82****^####^	>300	>300
CAP_PC42_20	PC42	20	>300	8.02 ± 6.05****^####^	>300	>300
CAP_BE45_5	BE45	5	>300	>300	>300	16.62 ± 2.86****
CAP_BE45_10	BE45	10	>300	>300	>300	18.42 ± 2.24****
CAP_BE45_20	BE45	20	>300	25.15 ± 4.85****	>300	>300
CAP_BPh40_5	BPh40	5	257.86 ± 6.14****^####^	17.59 ± 3.00**** ####	>300	41.05 ± 5.83****
CAP_BPh40_10	BPh40	10	>300	25.98 ± 3.52****	>300	27.86 ± 2.38****
CAP_BPh40_20	BPh40	20	>300	2.92 ± 6.97****^####^	>300	170.68 ± 5.03****

a The values are presented as mean ± SD (*n* = 3). Analysis of variance with Dunnett’s post hoc test was used to compare results between free drug and polymer-loaded with 5-fluorouracil (****p < 0.0001) and between two cell lines (####*p* < 0.0001). All statistics were calculated using the GraphPad Prism 8.

Since many reports in the literature over the past two decades delivered significantly different IC_50_ values and assessments of the effectiveness of 5-FU against HeLa cancer cells, ranging from 2.96^31^ to 105.63 μM (MGH)^32^ and 153.41 μM (Sanger)^32^ and over 200 μM,^33^ it is difficult to discuss our results with literature data. Therefore, we focused on the comparison of the efficacy of drug-polymer complexes depending on the polymer used, considering the obtained drug IC_50_ range (1.13–59.30 μM) as satisfactory compared to other literature data.

By comparing the IC_50_ values for free 5-FU and loaded within aryl-enriched hyperbranched polyglycidols, it can be seen that, except for the CAP_PC30_5 and CAP_BPh40_5 drug-polymer samples, which contain five drug molecules per macromolecule, 5-FU started to exert its anticancer effect after 48 h. Constructs based on drug-loaded polymers PC30, BPh40, PC42, and only BE45 showed the highest cytotoxicity against HeLa cells after 48 h of incubation. The most effective anticancer agents were observed for polymer samples loaded with 20 drug molecules per macromolecule and, perhaps surprisingly, those loaded with only five drug molecules per macromolecule.

All drug-loaded aryl-enriched HbPGLs turned out to be not toxic to noncancerous HMEC-1 cells after 24 h of incubation. It should be also noted that except for the 5-FU-loaded PC42 polymer, all drug-loaded HbPGLs were toxic to noncancerous HMEC-1 cells after 48 h. Among investigated aryl-enriched HbPGLs BE45 derivative turned out to be the most toxic to HMEC-1 cells after 48 h. It indicates that the presence of ester groups, instead of phenylurethane, in combination with drug causes the death of normal cells with no anticancer activity, as seen for BE 45 constructs with 5 and 10 drug molecules per macromolecule.

It is noteworthy, however, that all aryl-enriched HbPGLs loaded with 20 drug molecules per macromolecule exhibit selective anticervical cancer activity despite the used aryl moiety or a chemical linkage (ester or urethane) used for aryl immobilization, i.e., with a neutral effect against HMEC-1. The detailed structural analysis revealed that the anticancer selectivity was achieved merely for drug-loaded aryl-enriched hyperbranched polyglycidols, in which both the molar ratio of hydrophobized constitutional units and the molar ratio of monohydroxylated linear L_13_ and L_14_ units per drug molecule were the lowest, and were equal to 1 and 1.5, respectively (Table 2). It indicates that the molar ratio of all constitutional units present in aryl-enriched HbPGL derivatives can play a key role in the biological behavior of loaded 5-FU. It can result from the fact that 5-FU, as the uracil derivative, is equipped with both donor and acceptor of hydrogen bonds, which can participate in the interactions with ester, urethane, ether, hydroxyl, and diol groups. Therefore, the molar ratio of the functional groups should be taken into consideration in the estimation of the 5-FU biological activity. What is interesting, the selective anticancer properties were also detected for drug-loaded HbPGLs modified with phenylurethane groups, i.e., for PC30 sample loaded with five drug molecules and constructs of higher-modified phenylurethane derivative (PC42) equipped with both 5 and 10 drug molecules. For PC30 derivative with five drug molecules per one macromolecule, the molar ratio of both hydrophobized constitutional units and the molar ratio of monohydroxylated linear L_13_ and L_14_ units in HbPGL to a drug molecule were 2 and 6, respectively, whereas for PC42 derivative were equal to 3.6 aryl and 6 monohydroxylated units for a drug-loaded polymer construct with five drug molecules, and equal to 2 and 2.5 for construct 10 drug molecules per one macromolecule, respectively.

In general, it seems that aryl-enriched HbPGLs enhanced the solubility of 5-FU in aqueous medium and thus facilitated the transport of drug molecules into the cell. However, to obtain the selective anticancer behavior of 5-FU, it is crucial to ensure the optimal molar ratio of constitutional units of HbPGL to 5-FU.

**Figure 1:**
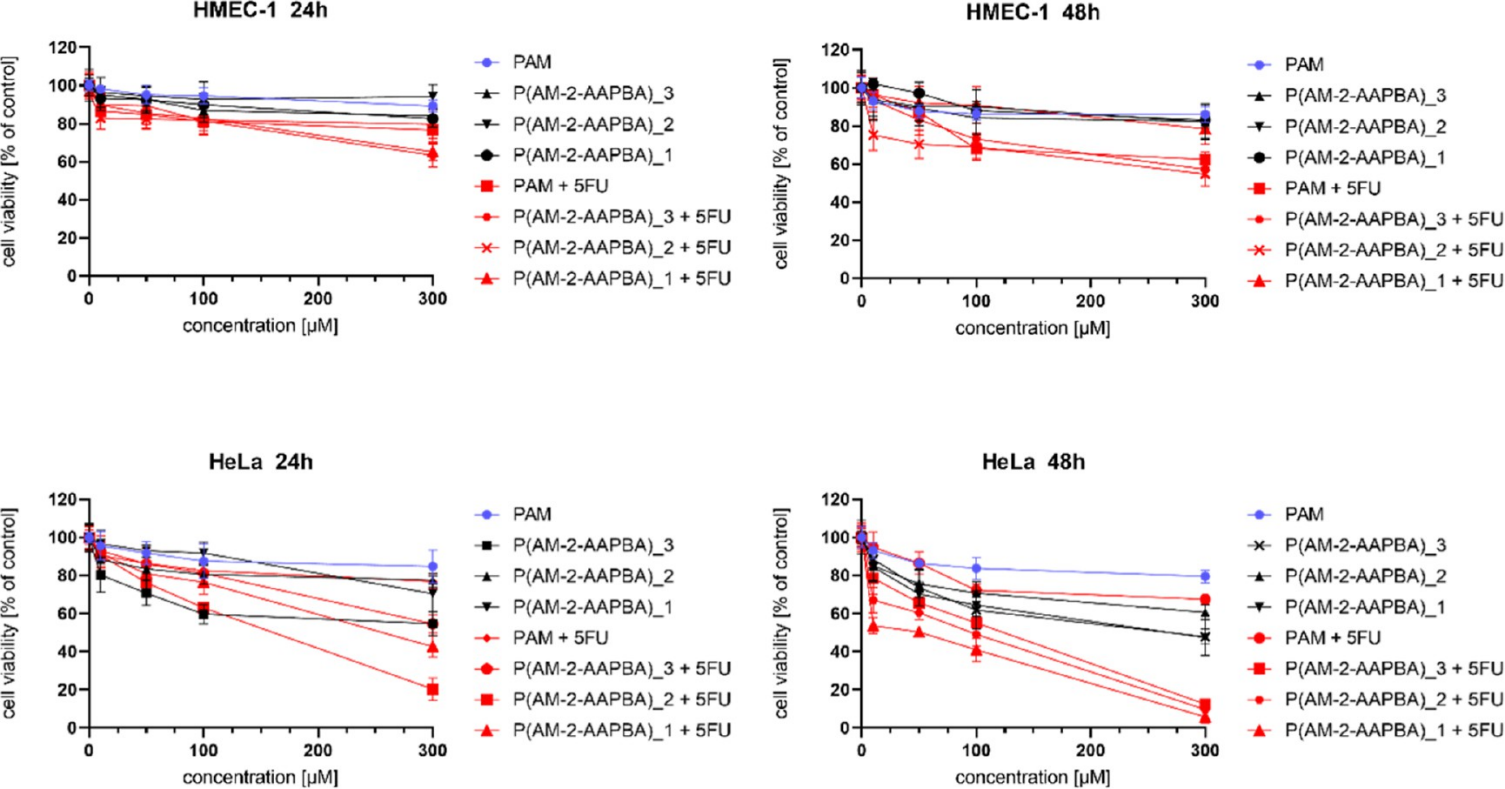
Influence of pure acrylamide homopolymer (PAM) and AM-2-AAPBA copolymers and their mixtures with 5-FU on HeLa (upper panel) and HMEC1 (lower panel) cells viability after 24 and 48 h incubation. Results are expressed as mean ± SD (*n* = 16).

In the next step of the investigations, the toxicity of neat and drug-loaded AM-2-AAPBA copolymers differing in the molar ratio of both comonomers against HeLa and HMEC-1 cell lines was evaluated. The study revealed the nontoxic effect of neat copolymer against both HeLa and HMEC-1 cells (Figure 1 and Table 4). What is interesting is the anti-HeLa selective action of drug-polymer formulations, in which the molar ratio of 5-FU to 2-AAPBA units was approximately equal to 0.6. This observation required, however, the verification of which comonomer, i.e., acrylamide or 2-AAPBA, was responsible for selective anticancer activity. For this goal, we investigated the toxicity of the mixture of acrylamide homopolymer (PAM) with 5-FU. Since both the homopolymer and its mixture with the drug were not toxic, 2-AAPBA units in the acrylamide copolymer are responsible for inducing the anticancer activity of the copolymer mixture with 5-FU. The usage of copolymers with different molar ratios of acrylamide to 2-AAPBA units facilitated the selection of the copolymer of the most favorable molar ratio of both comonomers, which supports the selective transport of 5-FU to HeLa cells (Figure 1). Table 4 shows the results of the IC_50_ parameter for the tested systems in the absence and presence of the drug. We observed the neutral effect of all investigated copolymers against HMEC-1 cells along with slight toxicity against HeLa cells. The selective anticancer activity of 5-FU-loaded poly(AM–*ran*–2–AAPBA) systems is highly advantageous in view of the construction of dynamic hydrogel systems based on aryl-enriched hyperbranched polyglycidols, in which the corona is equipped with 1,2-diol moieties in the terminal constitutional units. It is noteworthy that acrylamide copolymer with the lowest molar fraction of 2-AAPBA units, i.e., containing 10 mol % of 2-AAPBA was the most effective against cancer cells. Therefore, we decided to use this copolymer for the construction of dynamic hydrogels with 5-FU-loaded aryl-enriched HbPGLs. Probably, rarely distributed 2-AAPBA units along the backbone of macromolecules are more favorably interacting to block the mechanism of cancer cells proliferation.

**Table 4:** Comparison of IC_50_ Value for Acrylamide Homopolymer (PAM), Neat P(AM–2–AAPBA), and Their Mixtures with Loaded 5-Fluorouracil in HeLa and HMEC-1 Cell Lines^a^

		HeLa		HMEC-1	
polymer	5-FU	24 h	48 h	24 h	48 h
PAM	–	>300	>300	>300	>300
P(AM–2–AAPBA)_3	–	>300	247.36 ± 4.03	>300	>300
P(AM–2–AAPBA)_2	–	>300	>300	>300	>300
P(AM–2–AAPBA)_1	–	>300	235.17 ± 7.66	>300	>300
PAM	+	>300	>300	>300	>300
P(AM–2–AAPBA)_3	+	>300	148.60 ± 2.64	>300	>300
P(AM–2–AAPBA)_2	+	137.56 ± 4.93	103.80 ± 3.81	>300	>300
P(AM–2–AAPBA)_1	+	161.36 ± 8.48	33.32 ± 4.80	>300	>300

a The values are presented as mean ± SD (*n* = 3).

**Figure 2:**
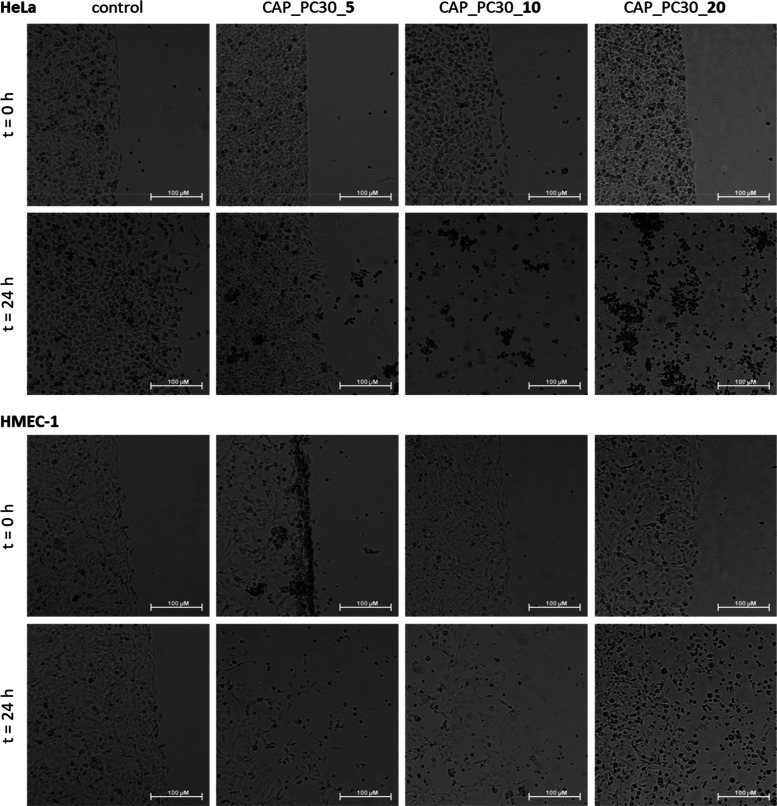
Influence of the hydrogels constructed of PC30 loaded with 5, 10, and 20 5-FU molecules, respectively, per macromolecule on HeLa (upper panel) and HMEC-1 (bottom panel) cell viability immediately after gel administration and after 24 h of incubation. Microscope magnification 4×, bar 100 μm.

**Figure 3:**
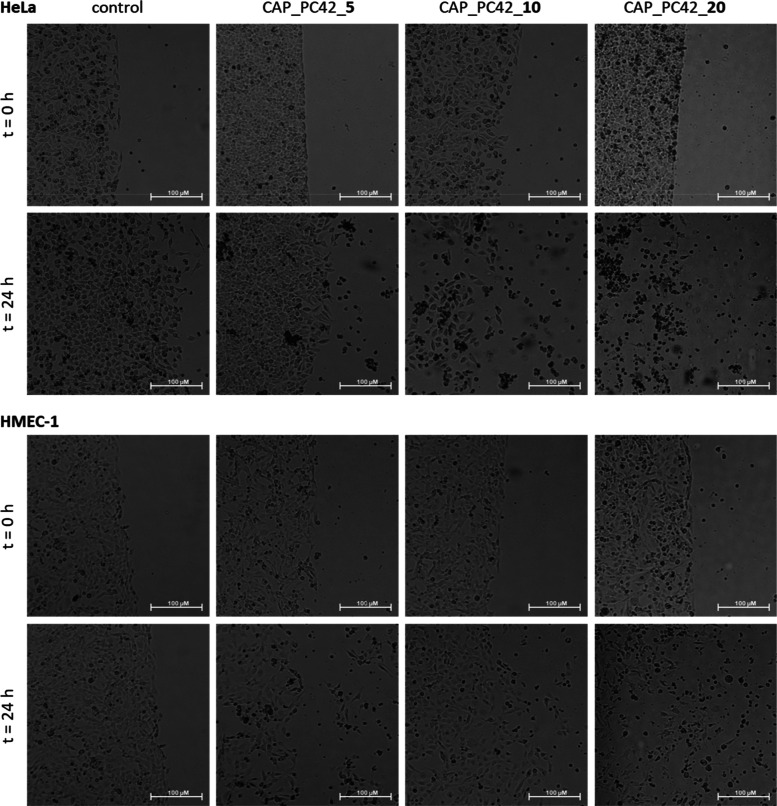
Influence of the hydrogels constructed of PC42 loaded with 5, 10, and 20 5-FU molecules, respectively, per macromolecule on HeLa (upper panel) and HMEC-1 (bottom panel) cell viability immediately after gel administration and after 24 h of incubation. Microscope magnification 4×, bar 100 μm.

**Figure 4:**
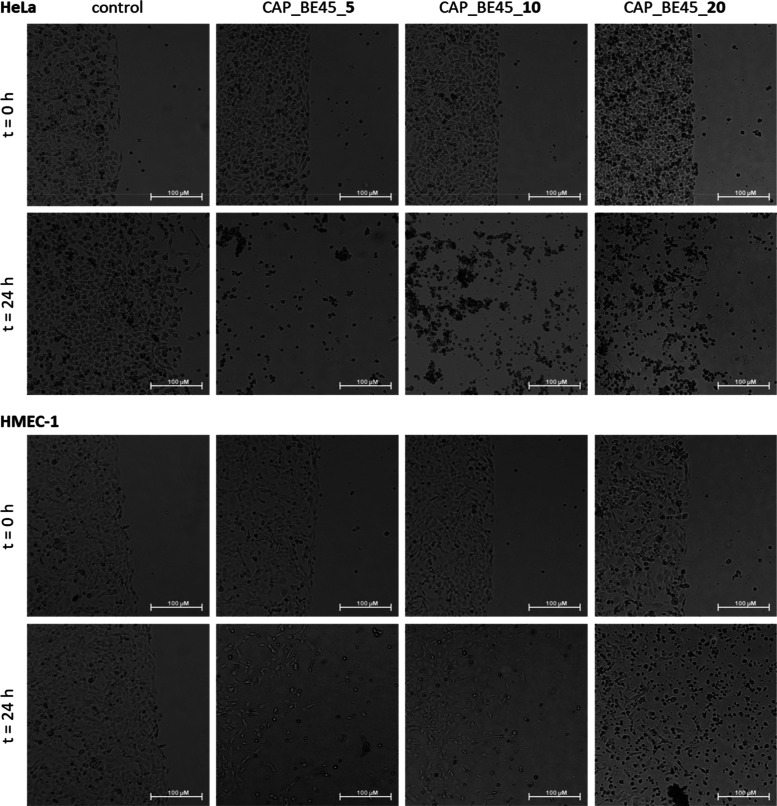
Influence of the hydrogels constructed of BE45 loaded with 5, 10, and 20 5-FU molecules per macromolecule on HeLa (upper panel) and HMEC-1 (bottom panel) cell viability immediately after gel administration and after 24 h of incubation. Microscope magnification 4×, bar 100 μm.

**Figure 5:**
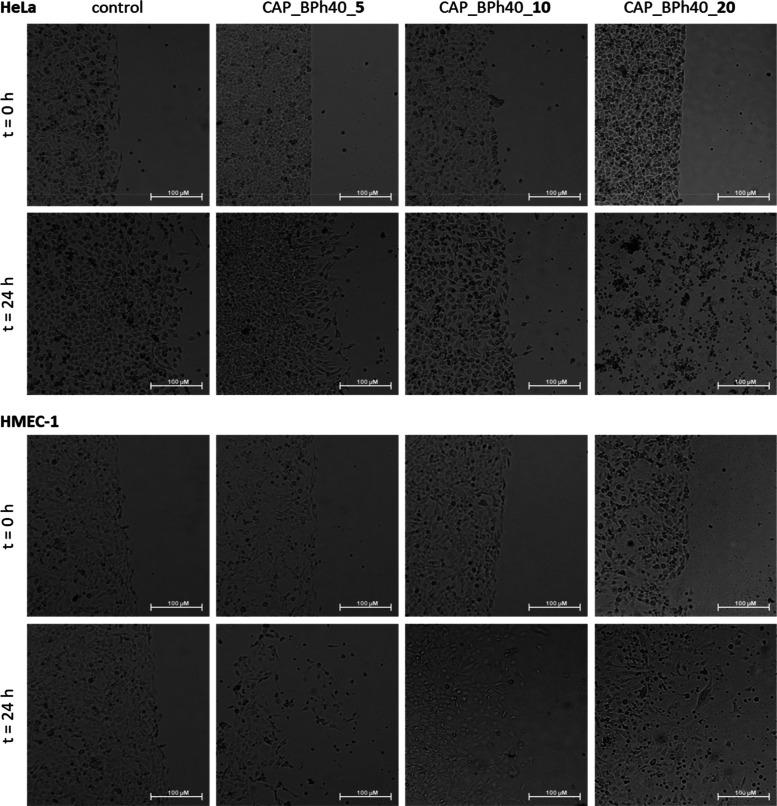
Influence of the hydrogels constructed of BPh40 loaded with 5, 10, and 20 5-FU molecules per macromolecule on HeLa (upper panel) and HMEC-1 (bottom panel) cell viability immediately after gel administration and after 24 h of incubation. Microscope magnification 4×, bar 100 μm.

The hydrogels constructed of dynamic cross-links between aryl-enriched HbPGLs and poly(AM–*ran*–2–AAPBA)_1 were at first investigated for their influence on the viability of both HeLa and HMEC-1 cell lines after 24 h. Using the ibidi system, gel was added to the cell-free space so that, after removal of the separator, the interaction between the gel and the cells at their interface could be observed from time *t* = 0. This form of gel application made it possible to avoid the effect of cutting off the cells from the culture medium by the gel layer. Images are shown in Supporting Information (Figure S18). Subsequently, the viability of both cell lines was assessed after 24 h exposure to drug-loaded hydrogels constructed of PC30, PC42, BE45, and BPh40 polymers containing 5, 10, and 20 5-FU molecules per macromolecule, respectively. Figures 2–5 show the microscopic images of control cells and cells treated with four series of hydrogels. To facilitate the evaluation of the images (Figures 2–5 and S32), we decided to use the ImageJ program^34^ to assess the area with the cells and determine its increase or decrease caused by the action of the tested gels (Tables S1 and S2).

Comparing the areas with the cells listed in Tables S1 and S2 after 24 h of culture, we observed that the number of untreated control cells increased by 65.4% in the HeLa cancer cell line and by 34.0% in the HMEC-1 noncancerous cell line. Neat hydrogels based on PC30, PC42, BE45, and BPh40 polymers were found to be practically harmless to both cell lines, slowing cell division to only a certain extent (Table 5). They increased the number of treated cells by 27.6%, 18.4%, 15.8%, and 4.1% in the HeLa cell line and by 13.3%, 16.8%, 12.2%, and 14.1% in the HMEC-1 cell line (Table 5), respectively.

**Table 5:** Comparison of the Changes in the Number of HeLa Cells and HMEC 1 Cells That Were Exposed for 24 h to the Tested Hydrogels Constructed of Neat and 5-Fluorouracil-Loaded Hyperbranched Polyglycidol Derivatives^a^

	HeLa cells (%)	HMEC 1 cells (%)
Control	65.4↑	34.0↑
pure gel PC30	27.6↑	13.3↑
CAP_PC30_5	98.9↓	1.9↓
CAP_PC30_10	9.4↑	24.7↓
CAP_PC30_20	97.6↓	21.7↓
pure gel PC42	18.4↑	16.8↑
CAP_PC42_5	51.0↓	23.5↑
CAP_PC42_10	7.8↑	6.2↓
CAP_PC42_20	98.7↓	14.9↓
pure gel BE45	15.8↑	12.2↑
CAP_BE45_5	99.8↓	6.1↓
CAP_BE45_10	99.9↓	28.4↓
CAP_BE45_20	94.1↓	36.1↓
pure gel BPh40	4.1↑	14.1↑
CAP_BPh40_5	11.4↑	2.5↑
CAP_BPh40_10	20.8↑	18.8↓
CAP_BPh40_20	99.6↓	2.6↑

a ↑—increase, ↓—decrease.

PC30 polymer-based gels containing 5 and 20 molecules of 5-FU per macromolecule caused a decrease in the number of treated cells in the HeLa cell line by 98.9% and 97.6%, respectively, while gels containing 10 molecules of 5-FU per macromolecule caused a 9.4% increase in HeLa cells number. In the HMEC-1 cell line, all PC30 polymer-based gels containing 5-FU decreased the number of treated cells by 1.9%, 24.7%, and 21.7%, respectively. Hydrogel-based formulations containing 5 and 20 drug molecules per macromolecule displayed selective anticancer properties similar to those of its 2AAPBA-free aqueous solutions.

Similarly, PC42 polymer-based hydrogels containing 5 and 20 molecules of 5-FU per macromolecule caused a decrease in the number of treated cells in the HeLa cell line by 51.0% and 98.7%, respectively, while gel containing 10 molecules of 5-FU per macromolecule caused an 7.8% increase in HeLa cells number. In the HMEC-1 line, only PC42 polymer-based gels containing 10 and 20 molecules of 5-FU decreased the number of treated cells by 6.2% and 14.9%, respectively. The least drug-loaded gel resulted in a 23.5% increase in the HMEC-1 cell number.

In the case of hydrogels composed of an ester derivative of HbPGL regardless of the used molar relationship of the drug molecules to one macromolecule, the incorporation of boronic acid motifs in the environment contributed to the selective anticervical cancer activity. Hydrogel systems based on BE45 loaded with 5, 10, and 20 drug molecules per macromolecule, respectively, caused a decrease in the number of treated cells in the HeLa cell line by 99.8%, 99.9%, and 94.1%, respectively. These gels also resulted in a certain loss of noncancerous HMEC-1 cells of 6.1%, 28.4%, and 36.1% percent, respectively.

In the case of hydrogels based on the BPh40 containing 5-FU, only the highest drug loading caused almost 100% mortality of cancer cells, while only inhibiting the proliferation of noncancerous cells. The other drug-loaded hydrogels constructed of BPh40, however, equipped with lower drug molecules per macromolecule, increased the number of cancer cells by 11.4% and 20.8%, respectively. It is noteworthy that these data are consistent with results obtained for hydrogels based on another urethane derivative, i.e., PC42, a derivative of comparable degree of modification and loaded with 10 molecules of 5-FU per macromolecule, which caused the 7.8% increase in cancer cells.

When comparing the percentages given, it is always necessary to keep in mind the rate of growth of the cancer cells that have not been treated with any of the compounds. A decrease in the proliferation of HeLa cells is clearly visible if we compare the 7–20% “increase” in the number of cancer cells after treatment with the tested gels with the value of the free growth HeLa cells at the level of 65%.

It is noteworthy that the incorporation of 5-FU loaded-aryl-enriched HbPGLs into the dynamic hydrogel network resulted in a much faster anticancer response (24 h) in comparison with 2-AAPBA-free constructs (48 h). Amazingly the action of drug closed in the network of dynamic structure of the hydrogel is significantly faster and effective than it was observed for the aqueous solutions of drug-loaded aryl-enriched HbPGLs or acrylamide copolymers. After the first 24 h, nearly all of the gels caused complete cancer cell death, allowing the gel exposure time to be shortened, thus increasing the protection of noncancerous cells while maintaining the therapeutic effect.

These data revealed that effective and selective anticancer properties of hydrogel systems are an effect of improved solubility of 5-FU in the aqueous medium ensured by the usage of aryl-enriched HbPGL; however, the rate of anticancer activity was significantly increased in the presence of 2-AAPBA copolymer. The selective activity of 5-FU loaded within aryl-enriched HbPGLs in the molar ratio 20 drug molecule to macromolecule was maintained in the hydrogel systems, but the anticancer action was immediate in comparison to 2-AABPA-free solutions. For these formulations, the molar ratio of 5-FU to 2-AAPBA units was the highest and approximately equal to 2.0 in the case of all investigated polymers (Table 6). In addition, the molar ratio of both hydrophobized and monohydroxylated constitutional units to 5-FU was the lowest among the investigated systems (Table 2). Among hydrogels constructed of all aryl-enriched HbPGLs, the formulations based on BE40 turned out to be the most selective against HeLa cell line, despite the used molar ratio of drug molecules per macromolecule.

**Table 6:** Composition of the Hydrogel Carriers of 5-Fluorouracil

hydrogel composition	molar ratio of drug to macromolecule	molar ratio of drug to 2-AAPBA
CAP_PC30_5/P(AM–*ran*–2–AAPBA)	5	0.51
CAP_PC30_10/P(AM–*ran*–2–AAPBA)	10	1.02
CAP_PC30_20/P(AM–*ran*–2–AAPBA)	20	2.00
CAP_PC42_5/P(AM–*ran*–2–AAPBA)	5	0.48
CAP_PC42_10/P(AM–*ran*–2–AAPBA)	10	0.95
CAP_PC42_20/P(AM–*ran*–2–AAPBA)	20	1.92
CAP_BE45_5/P(AM–*ran*–2–AAPBA)	5	0.48
CAP_BE45_10/P(AM–*ran*–2–AAPBA)	10	0.95
CAP_BE45_20/P(AM–*ran*–2–AAPBA)	20	1.92
CAP_BPh40_5/P(AM–*ran*–2–AAPBA)	5	0.44
CAP_BPh40_10/P(AM–*ran*–2–AAPBA)	10	0.84
CAP_BPh40_20 P(AM–*ran*–2–AAPBA)	20	1.72

To facilitate the comparison of the results described above, the changes in the cell numbers of both cell lines tested have been summarized in Table 5.

## 6 Computational Simulation of the Role of a Boronic Acid Motif in the Acceleration of Anticervical Cancer Activity

**Figure 6:**
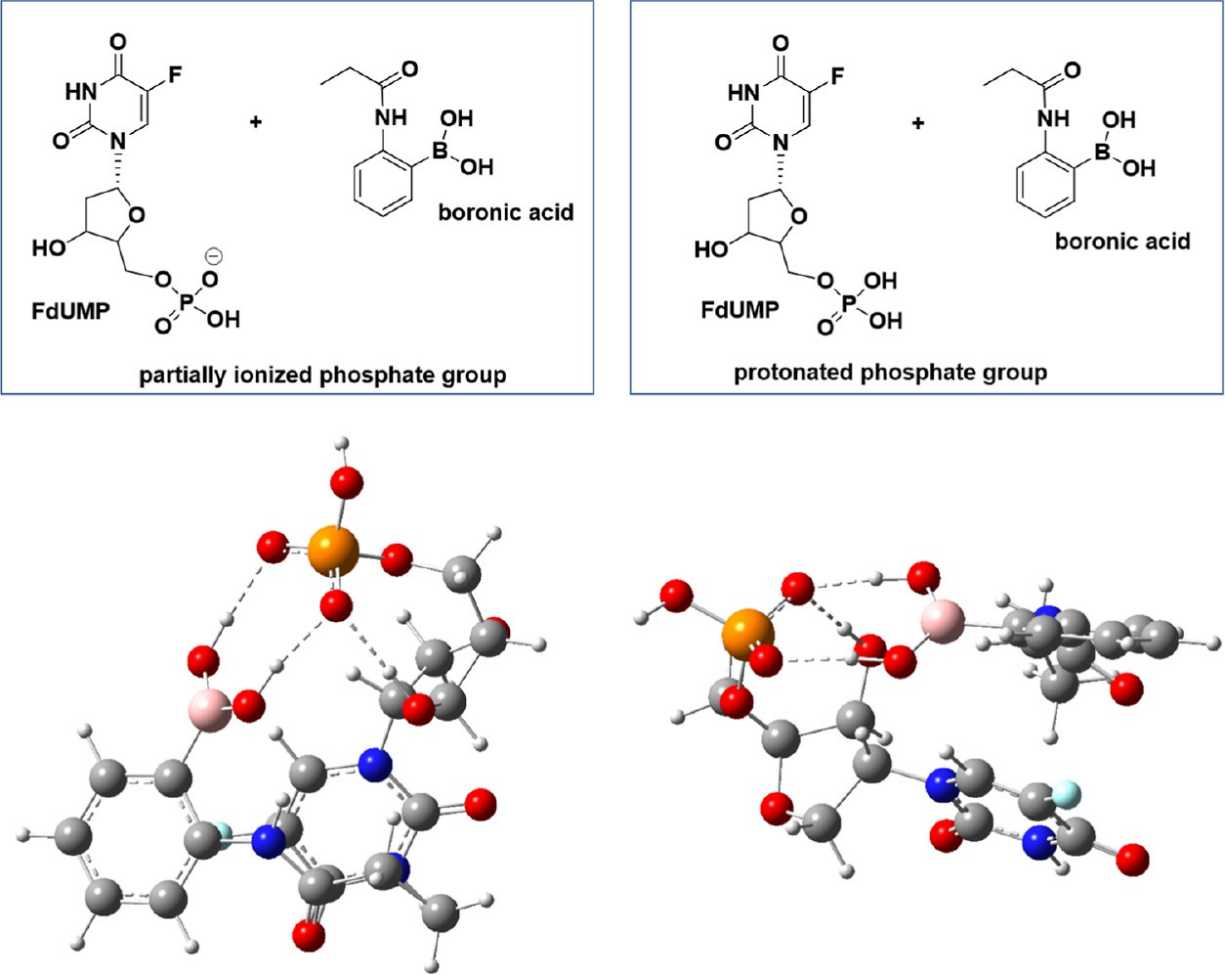
At the top, couples of boronic acid with a nucleotide of 5-FU with partially ionized and protonated phosphate residue. At the bottom, the structure of the most energetically stable noncovalently bonded complex of FdUMP with partially ionized phosphate group determined based on a computer simulation. Dashed lines depict the three hydrogen bonds formed by the partially ionized phosphate group with boronic acid.

Since the presence of 2-AAPBA moieties accelerated the anticancer behavior of 5-FU from 48 to 24 h, we focused on the role of boronic acid in the anticancer properties of this drug. We took into consideration the anticancer activity of 5-FU based on the inhibition of thymidylate synthase, i.e., the enzyme that catalyzes the reductive methylation of deoxyuridine monophosphate (dUMP) to thymidylate (TMP) using 5,10-methylene tetrahydrofolate as the methyl group donor, and we focused on the potential role of 2-AAPBA units in this mechanism. For this goal, we analyzed the structure of the active center of the synthase, in which various amino acids are present, including cysteine, tyrosine, and predominantly arginine containing a guanidinium fragment.^35^ We carried out a computer simulation study of the potential interactions of boronic acid with these amino acids. However, the study revealed that interactions between these couples are energetically unfavorable to occur in water (Δ*G* < 0). However, further computer simulation revealed the occurrence of an energetically favorable interaction of (2-propionamidophenyl) boronic acid with a nucleotide of 5-FU (FdUMP). The main reason for the formation of these complexes are hydrogen bonding, which are formed between -B(OH)_2_ and phosphate residue (protonized, H_2_PO_4_^–^ and partially ionized, HPO_4_^–^ forms), Figure 6. For the ionized form of phosphate in the nucleotide, three strong hydrogen bonds are formed between boronic acid and phosphate residue of the length equal to 1.7 Å and the average strength HB of each bond is 15.0 kcal/mol as estimated by eq 1,^6^ where ρ() denotes the total electron density in the (3, −1) bond critical point. The HB strength was estimated using Multiwfn software.^7^
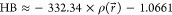
1

Δ*G* of such interactions of boronic acid and phosphate residue (protonated and partially ionized, ^–^H_2_PO_4_ and H_2_PO_4_^–^) is −7.9 and −2.3 kcal/mol, respectively.

Based on the data obtained, we can conclude that it is most likely, due to the interaction of boronic acid with a nucleotide of the drug (FdUMP), the copolymer enters the cell with the drug where its association ability can be increased due to complexation with the phosphate residue and can block the active site of the enzyme, thus accelerating the anticancer properties of 5-FU.

In addition, it is noteworthy that the complexation ability of boronic acid is increased with an increased concentration of phosphate buffer. Imaminato et al. reported that the reaction of 3-nitrophenylboronic acid with 1,2-diol group of Alizarin Red S was accelerated along with the increase of phosphate ions concentration.^36^ The reaction rate was enhanced in the presence of HPO_4_^2–^, whereas H_2_PO_4_^–^ hardly interacted with the boronic acid. These data input that the ability of boronic acid to complexation with different species, including amino acids, may be greater for boronic acid interacting with a phosphate group in the nucleotide than that in its free state.

## 7 Rheology Investigations of Dynamic Drug-Loaded Hydrogel Systems: Self-Healing Behavior

Rheological investigations of hydrogels were carried out at 25 and 37 °C. At first, due to the reversible nature of the cross-links in the network, we verified whether the addition of 5-FU influences the rheological properties of synthesized hydrogels.

**Figure 7:**
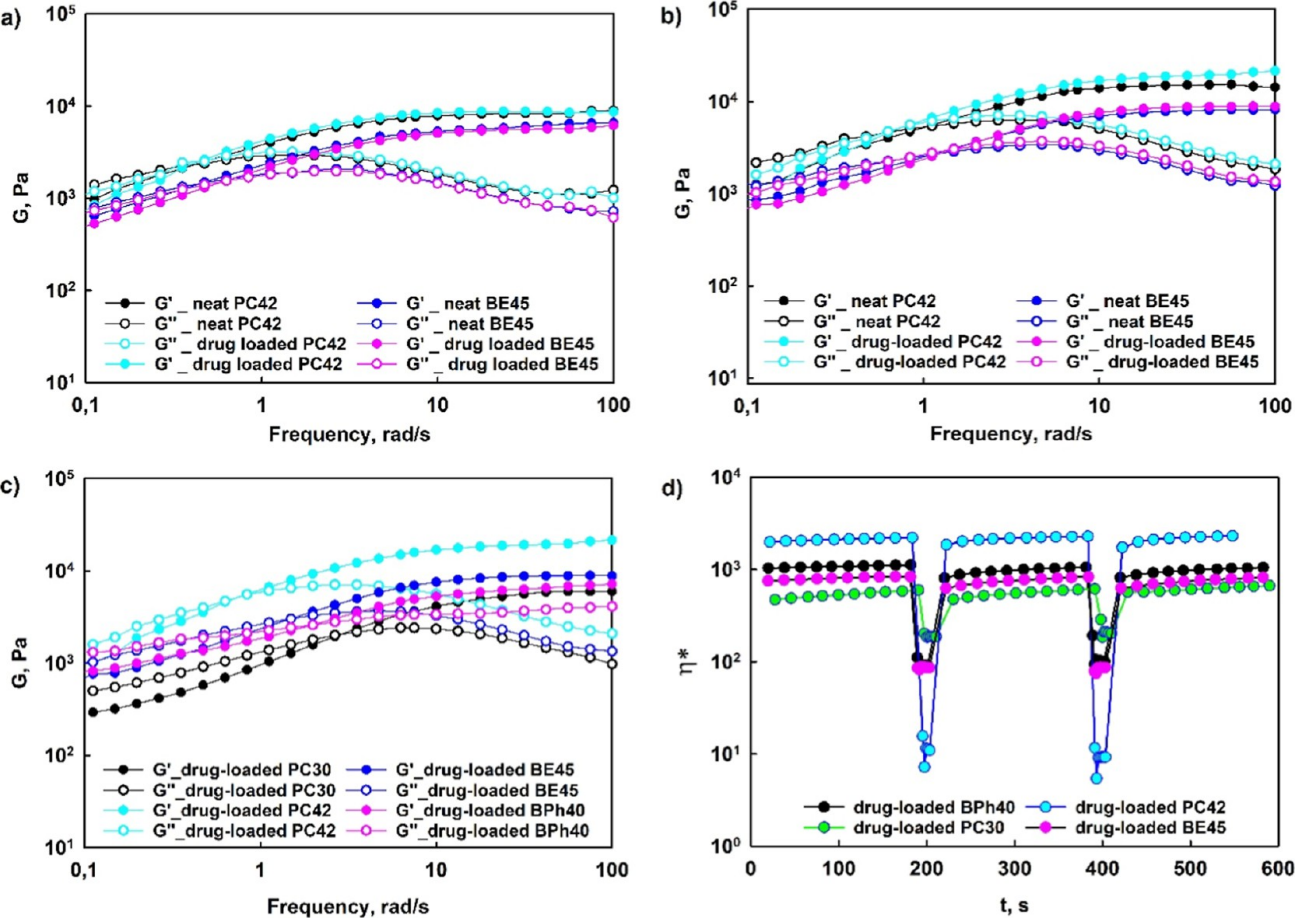
Frequency sweep tests performed at 25 (a) and 37 °C (b) for neat and drug-loaded hydrogels based on hyperbranched polyglycidol modified with phenyl groups incorporated via urethane and ester linkages. The comparison of frequency sweep tests performed at 37 °C for drug-loaded hydrogels constructed of hyperbranched polyglycidols modified with different aromatic groups (c). Time-dependent self-healing of drug-loaded hydrogels constructed of hyperbranched polyglycidols modified with different aromatic groups after failure (d).

Frequency sweep tests performed for neat and drug-loaded hydrogels prepared with HbPGLs modified with phenyl groups at comparable degrees, i.e., PC42 and BE45, via either urethane or ester linkages, showed that the presence of 5-FU in the network did not affect the rheological properties of the hydrogels despite temperature conditions (Figure 7a,b). Both the plateau storage modulus, *G*_N_ determined at the higher range of frequency, and the crossover of the angular frequency value at which *G*′ = *G*″ recorded for neat and drug-loaded hydrogels were identical.

The frequency sweep experiments revealed viscoelastic character for all investigated drug-loaded hydrogel systems (Figure 7c). In the higher frequency range, storage modulus, *G*′, exceeded the value of the loss modulus, *G*″, whereas the reduction of the frequency resulted in the inversion of *G*′ and *G*″ at the crossover frequency, ω_c_, which corresponds to the gelation point, i.e., gel–liquid transition as an effect of the equilibrium between boronic ester cross-links and substrates, i.e., a boronic acid and a diol. ω_c_ denotes the onset of the macroscopic chain displacement, therefore, at frequencies below ω_c_, the material starts flowing as an effect of the dominant contribution of viscous properties. Based on these experiments, we can conclude that all the hydrogels studied exhibit the dynamic behavior^37^ as the effective lifetime of cross-links (τ_R_) in all networks, determined according to the following relation τ_R_ = 1/2πω_c_,^38^ is below 1 min. Comparing the hydrogels based on phenylurethane derivatives (PC30 and PC42) shows that the use of the HbPGL derivative with a higher degree of modification resulted in the higher value of both the effective lifetime of bonds (τ_R_) and *G*_N_. It is noteworthy that among investigated hydrogels constructed of HbPGLs enriched with different aryl moieties, but at a comparable degree, *G*_N_ for PC42 was over twice higher than that observed for both BE45 and BPh40 (Table 7). Due to the fact that *G*_N_ is proportional to a network strand density (υ_χ_ = *G*_N_/R·T)^39^ in the case of PC42-based hydrogel beside boronic ester cross-links, the contribution of hydrophobic associations in the network formation is noticeable. Such a high value of *G*_N_ for the hydrogel based on PC42 derivative can be explained with more exposed aryl groups outside the HbPGL macromolecule in comparison to benzoyl ester groups in BE45 derivative which are effectively surrounded by water molecules,^40^ and thus the possibility of intermolecular hydrophobic associations is diminished. The mobility of the larger 1,4-biphenyl groups in BPh40 derivative may be more limited by polyether chains reducing the possibility of intermolecular hydrophobic association between aromatic rings.

**Table 7:** Rheological Parameters of Hydrogels Constructed of Drug-Loaded Aryl-Enriched Hyperbranched Polyglycidol Cross-Linked with P(AM–*ran*–2–AAPBA) Determined at 37 °C

hydrogel	*G*_N_, Pa	ω_c_, rad/s	τ, s	network strand density, υ_χ_, mol/m^3^
PC30	6000	2.89	0.055	2.33
PC42	20,560	0.74	0.215	7.98
BE45	8880	1.49	0.107	3.45
BPh40	7000	1.90	0.083	2.71

The self-healing experiments performed for drug-loaded hydrogels (Figure 7d) showed that upon the usage of a strain equal to 300%, a significant drop of viscosity was observed along with the inversion of both *G*′ and *G*″ (Figure S33) and thus dominant viscous properties corresponding to the network disruption. However, the decrease of applied strain to 1% resulted in the almost complete regeneration of the hydrogel structure after strain-induced failure as both *G*′ and *G*″ returned to their original values (*G*′ > *G*″) (Figure S33). The exemplary visualization test of self-healing behavior of aryl-enriched HbPGL-based hydrogel constructed of BE45 derivative is also demonstrated in Figure S34. The self-healing tests showed that each of the investigated hydrogel carriers can be injected to the target place as the viscosity of material can be decreased at the high shear rate (strain equal to 300%) and can be maintained on the afflicted tissue, thanks to high enough values of complex viscosity at the low-frequency region (Figure S35) that prevent uncontrolled leakage of the drug carrier.

The self-healing behavior of investigated hydrogels results from the reversible character of boronic ester formation and the equilibrium between boronic ester and boronic acid with diol. In spite of the fact that in the case of PC42-based hydrogel the contribution of hydrophobic association was noticeable, these interactions have a single sticker character without aggregate formation, which would restrict the regeneration of the polymer network.

## 8 Franz Cell Study

**Figure 8:**
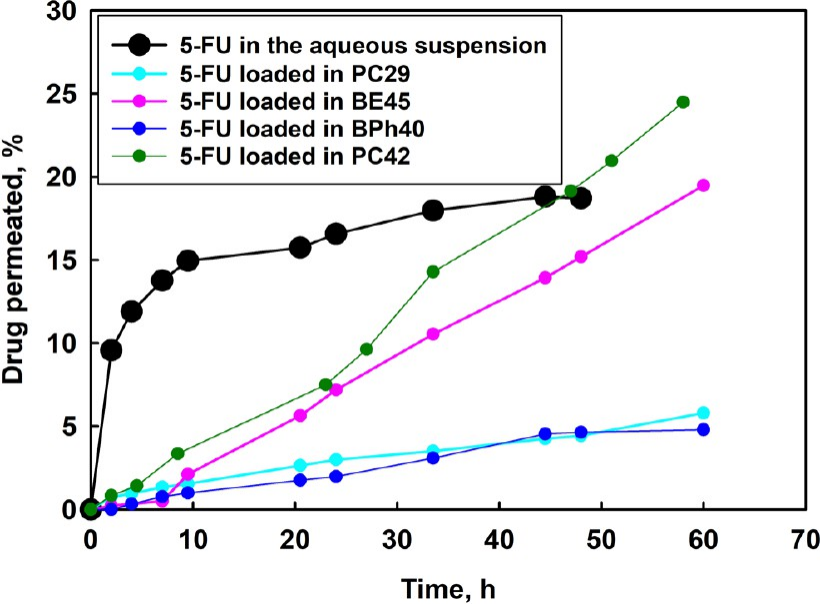
Results of a 5-FU permeability study of various drug formulations using the Strat-M membrane.

Due to the promising anticancer activity of 5-FU-loaded hydrogels regarding their selective and effective activity against cervical cancer cells, we performed in vitro permeability investigations of 5-FU across a Strat-M membrane, i.e., a synthetic nonanimal model membrane for transdermal diffusion which is used to predict the diffusion to the afflicted tissue. 5-FU-loaded hydrogel samples were mounted in the Franz diffusion cell. For comparison, we also investigated the behavior of free drug. Drug permeation studies conducted with Franz cell revealed that the drug suspended in water undergoes rapid permeation through the membrane being in contact with simulated vaginal fluid (Figure 8). The maximum amount of drug permeated (15% of the total amount of drug in the suspension) from the aqueous suspension penetrated the membrane after 9 h. In the case of hydrogel carriers, a smaller amount of drug permeated through the membrane compared to that of the free drug. Moreover, the drug released from benzoyl ester derivative (BE45) and phenylurethane derivative (PC42) permeated the membrane more efficiently, approximately 8%, than that in the case of PC30 and BPh40 derivatives (Figure S12), i.e., 3 and 2% of drug released, respectively. To compare the permeation of free 5-FU and loaded in the investigated hydrogel systems, we calculated the permeability constant, *K*_p_ (*K*_p_ = *Q*·*A*·*t*·*C*_o_, where *Q* is the amount of drug transported through the membrane in time *t*, *A* is the area-exposed membrane, and *C*_o_ is the donor concentration), and the flux, *J* = *Q*/*A*·*t* (Table 8). The permeability constant determined for all hydrogel systems was of the same order, whereas the permeability constant of free drug was one order higher, which results from a certain solubilization of 5-FU in water. The data of the dependence of percentage of drug permeated from aqueous suspension of 5-FU and drug loaded within hydrogels were fitted to known release kinetic models (Figures S36–S40). The release data obtained for hydrogels constructed of aryl-modified-HbPGL derivatives of comparable substitution degree of linear units in HbPGL were fitted to the zero-order model^41^ according to the equation: *Q*_*t*_ = *Q*_0_ + *k*_0_*t*, where *Q*_*t*_ is the amount of released drug at time *t*, *Q*_0_ is the initial amount of drug in the solution, and *k*_0_ is the constant of zero-order release.

**Table 8:** Comparison of the Permeability Constant (*K*_p_) and Steady State Flux (*J*) of 5-Fluorouracil Loaded in the Hydrogel Carriers and Suspended in an Aqueous Medium Using STRAT-M Membrane along with Fitted Kinetic Models to Drug Permeated Data

sample	*K*_p_ 5-FU, cm/min	*J*, mg/cm^2^ min	drug release kinetic model; constant of release
aqueous suspension of 5-FU	4.50 × 10^–5^	4.05 × 10^–4^	Korsmeyer-Peppas; *k*_*r*_ = 9.14
CAP_PC30_20/P(AM–2–AAPBA)	1.32 × 10^–6^	4.52 × 10^–5^	Korsmeyer-Peppas; *k*_*r*_ = 0.41
CAP_BE45_20/P(AM–2–AAPBA)	1.90 × 10^–6^	6.10 × 10^–5^	zero-order; *k*_0_ = 0.41
CAP_PC42_20/P(AM–2–AAPBA)	3.73 × 10^–6^	3.73 × 10^–5^	zero-order; *k*_0_ = 0.35
CAP_BPh40_20/P(AM–2–AAPBA)	1.00 × 10^–6^	2.90 × 10^–5^	zero-order; *k*_0_ = 0.09

The release data obtained for both PC30-based hydrogel and the aqueous suspension of 5-FU were fitted to the Korsmeyer-Peppas model^41^ expressed by the equation:  = *k*_*r*_*t*^*n*^, where  corresponds to the fraction of drug released at time *t*, *k*_*r*_ is the release constant, and *n* is the characteristic for the release mechanism.

The zero-order model is used for pharmaceutical drug carriers that do not disintegrate and have a very slow drug release. It indicates that hydrogels constructed of aryl-modified-HbPGL derivatives of comparable substitution degree of linear units in HbPGL maintain the integrity at the time of the Franz cell study, and the release was governed by the density of the polymer network. The fitting of the release data for the carrier based on PC30 derivative to the Korsmeyer-Peppas model with a value of *n* equal to 0.61 showed that the partial matrix dissolution occurred for this hydrogel sample, and the release mechanism corresponds to an anomalous (non-Fickian) transport.^42^

## 9 Conclusions

In summary, after screening multiple systems, hydrogel carriers of 5-FU, which endowed the drug with both activity and selectivity against cervical cancer cells, have been elaborated to surmount the challenge of the lack of activity of 5-FU in the therapy of cervical cancer.

The drug was loaded within amphiphilic hyperbranched polyglycidols obtained by the modification of the linear monohydroxylated units in the interior of the macromolecules with aromatic groups such as phenyl and 1,4-biphenyl moieties immobilized via ester or urethane linkages.

The molar ratio of 5-FU to aryl-modified formulations turned out to be crucial to obtaining the most effective and selective formulations. The most favorable anticervical cancer activity was shown by both aqueous solution and hydrogel-based formulations in which 20 mol drugs were loaded per mole of polymer. All aqueous solutions of drug-loaded aryl-enriched HbPGLs displayed anticancer activity after 48 h, but only the group of HbPGL modified with phenylurethane moieties showed selectivity in terms of toxicity only toward cancer cells, regardless of the number of drug molecules encapsulated. The incorporation of drug-loaded hyperbranched polyglycidols into the hydrogel, obtained by their cross-linking with acrylamide copolymers of 2-AAPBA, resulted in almost complete mortality of cancer cells after just 24 h, particularly in formulations containing 20 mol encapsulated drug per mole of the polymer. For accelerated anticancer activity of drug-polymer constructs in the hydrogel matrix, boronic acid was responsible. Among all investigated aryl-enriched HbPGLs, hydrogels constructed of hyperbranched polyglycidol modified with benzoyl ester groups loaded with the drug ensured selective almost complete mortality of cervical cancer cells despite the investigated molar ratio of drug to the polymer.

The usage of reversible cross-links to the formation of dynamic hydrogels ensures the self-healing ability of systems despite the type of used aryl-enriched derivative of HbPGL, which can ensure the formation of a continuous hydrogel layer on the afflicted area of tissue. In addition, a permeability study revealed that 5-FU is released in a controlled manner. Among all investigated hydrogel systems, the drug is released to the highest extent from ester derivative of HbPGL.

The proposed hydrogel design makes 5-FU-based monotherapy for the treatment of cervical cancer possible, avoiding the application of additional bioactive compounds that are associated with adverse side effects.

## Supporting Information Available

^1^H NMR, ^1^H DOSY NMR spectra,^13^C INVGATE NMR, and DEPT spectra; GPC chromatograms recorded for synthesized polymers; data on both HeLa and HMEC-1 cell viability on the concentration of 5-FU suspended in water or released from hydrogel carriers; self-healing tests; data of the complex viscosity of the investigated hydrogels on the angular frequency; and fitting the 5-FU release data from hydrogel carriers and the aqueous suspension to the mathematical models (PDF)

## References

[ref1] Gosecka M.; Gosecki M.; Ziemczonek P.; Urbaniak M.; Wielgus E.; Marcinkowska M.; Janaszewska A.; Klajnert-Maculewicz B. Selective Anticervical Cancer Injectable and Self-Healable Hydrogel Platforms Constructed of Drug-Loaded Cross-Linkable Unimolecular Micelles in a Single and Combination Therapy. ACS Appl. Mater. Interfaces 2024, 16 (12), 14605–14625. 10.1021/acsami.4c01524. 38488848 PMC10982937

[ref2] Gosecka M.; Gosecki M.; Jaworska-Krych D. Hydrophobized Hydrogels: Construction Strategies, Properties, and Biomedical Applications. Adv. Funct. Mater. 2023, 33 (25), 2212302 10.1002/adfm.202212302.

[ref3] Jaworska-Krych D.; Gosecka M.; Gosecki M.; Urbaniak M.; Dzitko K.; Ciesielska A.; Wielgus E.; Kadlubowski S.; Kozanecki M. Enhanced Solubility and Bioavailability of Clotrimazole in Aqueous Solutions with Hydrophobized Hyperbranched Polyglycidol for Improved Antifungal Activity. Acs Appl. Mater. Interfaces 2024, 16 (15), 18434–18448. 10.1021/acsami.3c19388. 38579182 PMC11040572

[ref4] Gosecki M.; Ziemczonek P.; Gosecka M.; Urbaniak M.; Wielgus E.; Marcinkowska M.; Janaszewska A.; Klajnert-Maculewicz B. Cross-linkable star-hyperbranched unimolecular micelles for the enhancement of the anticancer activity of clotrimazole. J. Mater. Chem. B 2023, 11 (24), 5552–5564. 10.1039/D2TB02629E. 36877094

[ref5] Banerjee P.; Mukherjee D.; Maiti T. K.; Sarkar N. Unveiling the Self-Assembling Behavior of 5-Fluorouracil and its-Dimethyl Derivative: A Spectroscopic and Microscopic Approach. Langmuir 2017, 33 (41), 10978–10988. 10.1021/acs.langmuir.7b02378. 28930474

[ref6] Heidelberger C.; Chaudhuri N. K.; Danneberg P.; Mooren D.; Griesbach L.; Duschinsky R.; Schnitzer R. J.; Pleven E.; Scheiner J. Fluorinated Pyrimidines, a New Class of Tumour-Inhibitory Compounds. Nature 1957, 179 (4561), 663–666. 10.1038/179663a0. 13418758

[ref7] Ansfield F.; Klotz J.; Nealon T.; Ramirez G.; Minton J.; Hill G.; Wilson W.; Davis H. Jr.; Cornell G. A phase III study comparing the clinical utility of four regimens of 5-fluorouracil: a preliminary report. Cancer 1977, 39 (1), 34–40. 10.1002/1097-0142(197701)39:1<34::AID-CNCR2820390107>3.0.CO;2-2. 318916

[ref8] Ansfield F. J. A LESS TOXIC FLUOROURACIL DOSAGE SCHEDULE. JAMA 1964, 190, 686–688. 10.1001/jama.1964.03070200122030. 14217470

[ref9] Longley D. B.; Harkin D. P.; Johnston P. G. 5-Fluorouracil: Mechanisms of action and clinical strategies. Nat. Rev. Cancer 2003, 3 (5), 330–338. 10.1038/nrc1074. 12724731

[ref10] Macmillan W. E.; Wolberg W. H.; Welling P. G. Pharmacokinetics of Fluorouracil in Humans. Cancer Res. 1978, 38 (10), 3479–3482. 688233

[ref11] Sethy C.; Kundu C. N. 5-Fluorouracil (5-FU) resistance and the new strategy to enhance the sensitivity against cancer: Implication of DNA repair inhibition. Biomed. Pharmacother. 2021, 137, 111285 10.1016/j.biopha.2021.111285. 33485118

[ref12] Tigu A. B.; Toma V. A.; Mot A. C.; Jurj A.; Moldovan C. S.; Fischer-Fodor E.; Berindan-Neagoe I.; Pârvu M. The Synergistic Antitumor Effect of 5-Fluorouracil Combined with Allicin against Lung and Colorectal Carcinoma Cells. Molecules 2020, 25 (8), 1947 10.3390/molecules25081947. 32331446 PMC7221923

[ref13] Kaern J.; Trope C.; Abeler V.; Iversen T.; Kjorstad K. A Phase-Ii Study of 5-Fluorouracil Cisplatinum in Recurrent Cervical-Cancer. Acta Oncol. 1990, 29 (1), 25–28. 10.3109/02841869009089987. 2310600

[ref14] Desravines N.; Hsu C. H.; Mohnot S.; Sahasrabuddhe V.; House M.; Sauter E.; O’Connor S.; Bauman J. E.; Chow H. H. S.; Rahangdale L. Feasibility of 5-fluorouracil and imiquimod for the topical treatment of cervical intraepithelial neoplasias (CIN) 2/3. Int. J. Gynecol. Obstet. 2023, 163 (3), 862–867. 10.1002/ijgo.14983. PMC10782812 37431689

[ref15] Huang Y.; Wan X. W.; Du Y. T.; Feng Y.; Yang L. S.; Liu Y. B.; Chen T.; Zhu Z.; Xu Y. T.; Wang C. C. Norcantharidin Enhances the Antitumor Effect of 5-Fluorouracil by Inducing Apoptosis of Cervical Cancer Cells: Network Pharmacology, Molecular Docking, and Experimental Validation. Curr. Issues Mol. Biol. 2024, 46 (5), 3906–3918. 10.3390/cimb46050242. 38785510 PMC11120450

[ref16] Abdellatif A. A. H.; Mohammed A. M.; Saleem I.; Alsharidah M.; Al Rugaie O.; Ahmed F.; Osman S. K. Smart Injectable Chitosan Hydrogels Loaded with 5-Fluorouracil for the Treatment of Breast Cancer. Pharmaceutics 2022, 14 (3), 661 10.3390/pharmaceutics14030661. 35336035 PMC8950008

[ref17] Yun Q.; Wang S. S.; Xu S.; Yang J. P.; Fan J.; Yang L. L.; Chen Y.; Fu S. Z.; Wu J. B. Use of 5-Fluorouracil Loaded Micelles and Cisplatin in Thermosensitive Chitosan Hydrogel as an Efficient Therapy against Colorectal Peritoneal Carcinomatosis. Macromol. Biosci. 2017, 17( (4), ). 10.1002/mabi.201600262. 27762505

[ref18] Al Sabbagh C.; Seguin J.; Agapova E.; Kramerich D.; Boudy V.; Mignet N. Thermosensitive hydrogels for local delivery of 5-fluorouracil as neoadjuvant or adjuvant therapy in colorectal cancer. Eur. J. Pharm. Biopharm. 2020, 157, 154–164. 10.1016/j.ejpb.2020.10.011. 33222768

[ref19] Aycan D.; Gül I.; Yorulmaz V.; Alemdar N. Gelatin microsphere-alginate hydrogel combined system for sustained and gastric targeted delivery of 5-fluorouracil. Int. J. Biol. Macromol. 2024, 255, 128022 10.1016/j.ijbiomac.2023.128022. 37972837

[ref20] Dutta G.; Chinnaiyan S. K.; Palaniyandi T.; Sugumaran A.; Narayanasamy D. Biogenic synthesized CuO nanoparticles and 5-fluorouracil loaded anticancer gel for HeLa cervical cancer cells. Discover Nano 2024, 19 (1), 217 10.1186/s11671-024-04166-7. 39729148 PMC11680562

[ref21] Chen W.; Shi K.; Liu J.; Yang P. P.; Han R. X.; Pan M.; Yuan L. P.; Fang C.; Yu Y. Y.; Qian Z. Y. Sustained co-delivery of 5-fluorouracil and cis-platinum via biodegradable thermo-sensitive hydrogel for intraoperative synergistic combination chemotherapy of gastric cancer. Bioact. Mater. 2023, 23, 1–15. 10.1016/j.bioactmat.2022.10.004. 36406247 PMC9650011

[ref22] Türk H.; Shukla A.; Alves Rodrigues P.; Rehage H.; Haag R. Water-soluble dendritic core-shell-type architectures based on polyglycerol for solubilization of hydrophobic drugs. Chem.—Eur. J. 2007, 13 (15), 4187–4196. 10.1002/chem.200601337. 17310496

[ref23] Bannwarth C.; Ehlert S.; Grimme S. GFN2-xTB-An Accurate and Broadly Parametrized Self-Consistent Tight-Binding Quantum Chemical Method with Multipole Electrostatics and Density-Dependent Dispersion Contributions. J. Chem. Theory Comput. 2019, 15 (3), 1652–1671. 10.1021/acs.jctc.8b01176. 30741547

[ref24] Bannwarth C. C. E.; Caldeweyher E.; Ehlert S.; Hansen A.; Pracht P.; Seibert J.; Spicher S.; Grimme S. Extended tight-binding quantum chemistry methods. Wiley Interdiscip. Rev. Comput. Mol. Sci. 2020, 11, e01493 10.1002/wcms.1493.

[ref25] Neese F. Software update: The ORCA program system-Version 5.0. Wiley Interdiscip. Rev. Comput. Mol. Sci. 2022, 12 (5), e1606 10.1002/wcms.1606.

[ref26] Pracht P.; Bohle F.; Grimme S. Automated exploration of the low-energy chemical space with fast quantum chemical methods. Phys. Chem. Chem. Phys. 2020, 22 (14), 7169–7192. 10.1039/C9CP06869D. 32073075

[ref27] Frisch M. J.; Trucks G. W.; Schlegel H. B.; Scuseria G. E.; Robb M. A.; Cheeseman J. R.; Scalmani G.; Barone V.; Petersson G. A.; Nakatsuji H.; Li X.; Caricato M.; Marenich A. V.; Bloino J.; Janesko B. G.; Gomperts R.; Mennucci B.; Hratchian H. P.; Ortiz J. V.; Izmaylov A. F.; Sonnenberg J. L.; Williams-Young D.; Ding F.; Lipparini F.; Egidi F.; Goings J.; Peng B.; Petrone A.; Henderson T.; Ranasinghe D.; Zakrzewski V. G.; Gao J.; Rega N.; Zheng G.; Liang W.; Hada M.; Ehara M.; Toyota K.; Fukuda R.; Hasegawa J.; Ishida M.; Nakajima T.; Honda Y.; Kitao O.; Nakai H.; Vreven T.; Throssell K.; Montgomery J. A. Jr.; Peralta J. E.; Ogliaro F.; Bearpark M. J.; Heyd J. J.; Brothers E. N.; Kudin K. N.; Staroverov V. N.; Keith T. A.; Kobayashi R.; Normand J.; Raghavachari K.; Rendell A. P.; Burant J. C.; Iyengar S. S.; Tomasi J.; Cossi M.; Millam J. M.; Klene M.; Adamo C.; Cammi R.; Ochterski J. W.; Martin R. L.; Morokuma K.; Farkas O.; Foresman J. B.; Fox D. J. Gaussian 16, Revision C.01; Gaussian, Inc.: Wallingford CT, 2016.

[ref28] Kainthan R. K.; Janzen J.; Kizhakkedathu J. N.; Devine D. V.; Brooks D. E. Hydrophobically derivatized hyperbranched polyglycerol as a human serum albumin substitute. Biomaterials 2008, 29 (11), 1693–1704. 10.1016/j.biomaterials.2007.11.030. 18194812

[ref29] Kainthan R. K.; Hester S. R.; Levin E.; Devine D. V.; Brooks D. E. biological evaluation of high molecular weight hyperbranched polyglycerols. Biomaterials 2007, 28 (31), 4581–4590. 10.1016/j.biomaterials.2007.07.011. 17688941

[ref30] Kainthan R. K.; Brooks D. E. biological evaluation of high molecular weight hyperbranched polyglycerols. Biomaterials 2007, 28 (32), 4779–4787. 10.1016/j.biomaterials.2007.07.046. 17706767

[ref31] Takara K.; Sakaeda T.; Yagami T.; Kobayashi H.; Ohmoto N.; Horinouchi M.; Nishiguchi K.; Okumura K. Cytotoxic effects of 27 anticancer drugs in HeLa and MDR1-overexpressing derivative cell lines. Biol. Pharm. Bull. 2002, 25 (6), 771–778. 10.1248/bpb.25.771. 12081145

[ref32] www.cancerrxgene.org (accessed November 17, 2024).

[ref33] Mavrikou S.; Tsekouras V.; Karageorgou M. A.; Moschopoulou G.; Kintzios S. Detection of Superoxide Alterations Induced by 5-Fluorouracil on HeLa Cells with a Cell-Based Biosensor. Biosensors 2019, 9 (4), 126 10.3390/bios9040126. 31623083 PMC6956086

[ref34] Schneider C. A.; Rasband W. S.; Eliceiri K. W. NIH Image to ImageJ: 25 years of image analysis. Nat. Methods 2012, 9 (7), 671–675. 10.1038/nmeth.2089. 22930834 PMC5554542

[ref35] Carreras C. W.; Santi D. V. The catalytic mechanism and structure of thymidylate synthase. Annu. Rev. Biochem. 1995, 64, 721–762. 10.1146/annurev.bi.64.070195.003445. 7574499

[ref36] Imaminato K.; Kusuyama D.; Suzuki S.; Sugaya T.; Ishihara K. Influence of Phosphate Buffer on the Reaction of 3-Nitrophenylboronic Acid with Alizarin Red S. ChemistrySelect 2023, 8 (30), e202302600 10.1002/slct.202302600.

[ref37] Aida T.; Meijer E. W.; Stupp S. I. Functional Supramolecular Polymers. Science 2012, 335 (6070), 813–817. 10.1126/science.1205962. 22344437 PMC3291483

[ref38] Herbst F.; Döhler D.; Michael P.; Binder W. H. Self-Healing Polymers via Supramolecular Forces. Macromol. Rapid Commun. 2013, 34 (3), 203–220. 10.1002/marc.201200675. 23315930

[ref39] Feldman K. E.; Kade M. J.; Meijer E. W.; Hawker C. J.; Kramer E. J. Model Transient Networks from Strongly Hydrogen-Bonded Polymers (vol 42, pg 9072, 2009). Macromolecules 2011, 44 (13), 5537 10.1021/ma201245h.

[ref40] Jaworska-Krych D.; Gosecka M.; Maczugowska P.; Halagan K.; Szutkowski K.; Gosecki M.; Urbaniak M.; Kozanecki M. Thermosensitive Behavior of Internally Phenyl-Enriched Hyperbranched Polyglycidols in Water-The Influence of a Covalent Bond Used for the Hydrophobization. Macromolecules 2024, 57 (19), 9135–9156. 10.1021/acs.macromol.4c01709.

[ref41] Salamanca C. H.; Barrera-Ocampo A.; Lasso J. C.; Camacho N.; Yarce C. J. Franz Diffusion Cell Approach for Pre-Formulation Characterisation of Ketoprofen Semi-Solid Dosage Forms. Pharmaceutics 2018, 10 (3), 148 10.3390/pharmaceutics10030148. 30189634 PMC6161298

[ref42] Dash S.; Murthy P. N.; Nath L.; Chowdhury P. Kinetic modeling on drug release from controlled drug delivery systems. Acta Polym. Pharm. 2010, 67 (3), 217–223. 20524422

