# Supplementary material for: Construction of Dynamic Hydrogel Inducing Effective and Selective 5-Fluorouracil Monotherapy against Cervical Cancer Cells
Source: ACS Appl Mater Interfaces. 2025 Feb 21;17(9):13427–44. doi: 10.1021/acsami.4c20276 (PMC11891837; doi:10.1021/acsami.4c20276)
Supplement: Supplementary file 1 — am4c20276_si_001.pdf [file am4c20276_si_001.pdf]

Supporting Information

for

Construction of dynamic hydrogel inducing effective and selective 5-fluorouracil monotherapy against cervical cancer cells

Monika Gosecka[a]*, Daria Jaworska-Krych[a], Mateusz Gosecki[a], Malgorzata Urbaniak[a], Ewelina Wielgus[a], Bartlomiej Gostynski[a], Monika Marcinkowska[b], Anna Janaszewska[b]*, Barbara Klajnert-Maculewicz[b]

a. Centre of Molecular and Macromolecular Studies, Polish Academy of Sciences Sienkiewicza 112, 90-363 Lodz, Poland

* Correspondence: mdybko@cbmm.lodz.pl

b. Department of General Biophysics, Faculty of Biology and Environmental Protection, University of Lodz, 141/143 Pomorska Street, 90-236 Lodz, Poland

* Correspondence: anna.janaszewska@biol.uni.lodz.pl

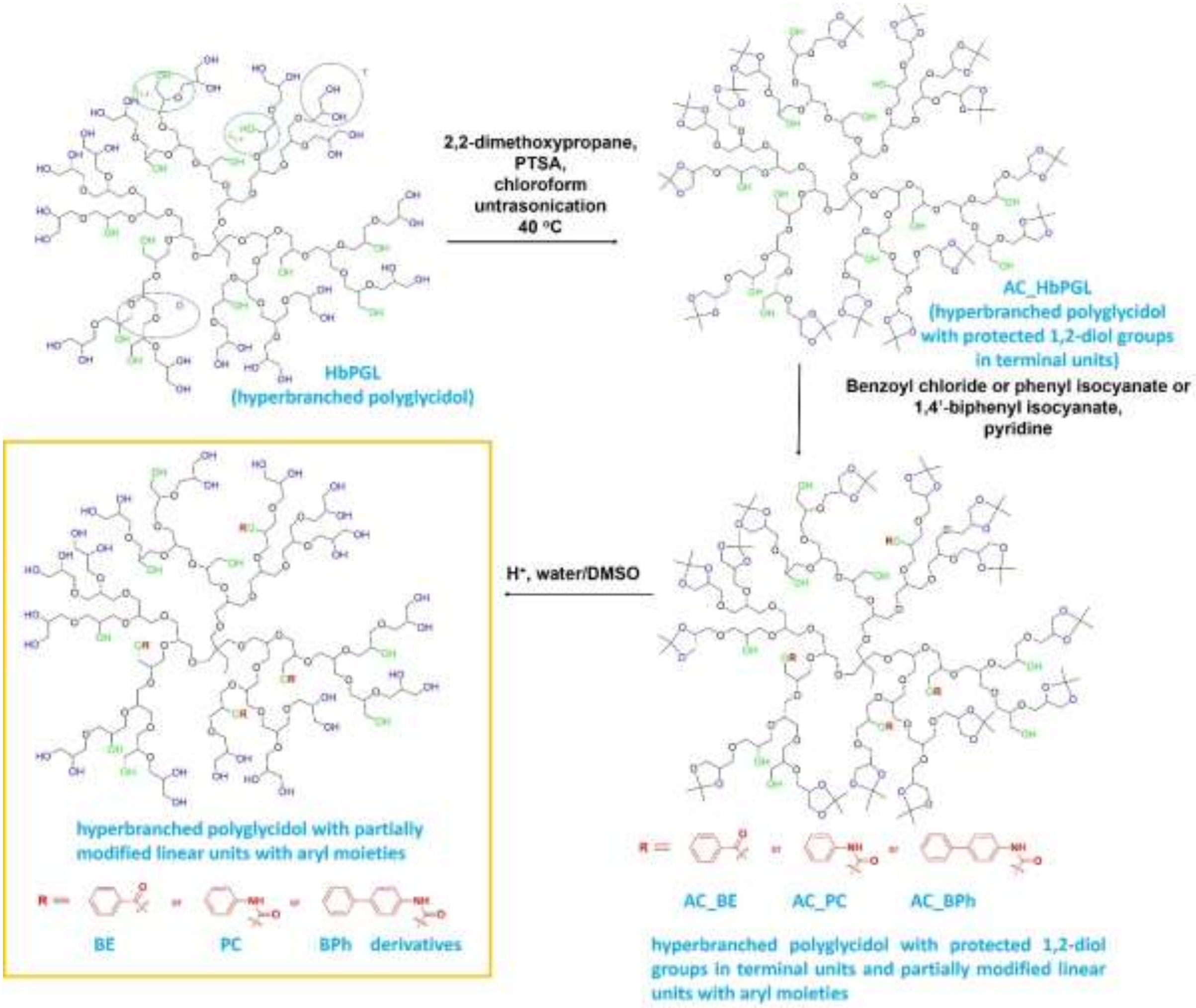

Scheme S1. Schematic representation of multistep process resulting in the formation of aryl-enriched hyperbranched polyglycidol.

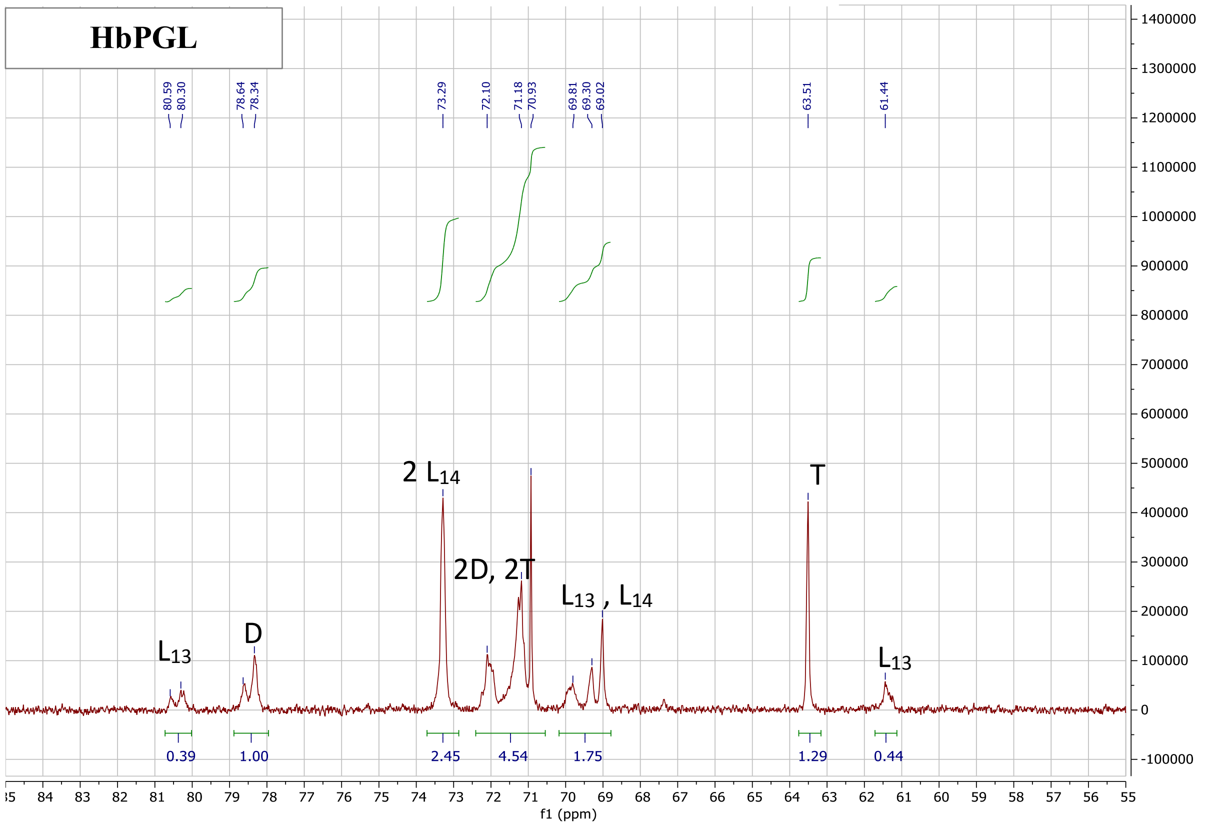

Figure S1. ^{13}C INVGATE NMR spectrum of HbPGL recorded in DMSO-d_6.

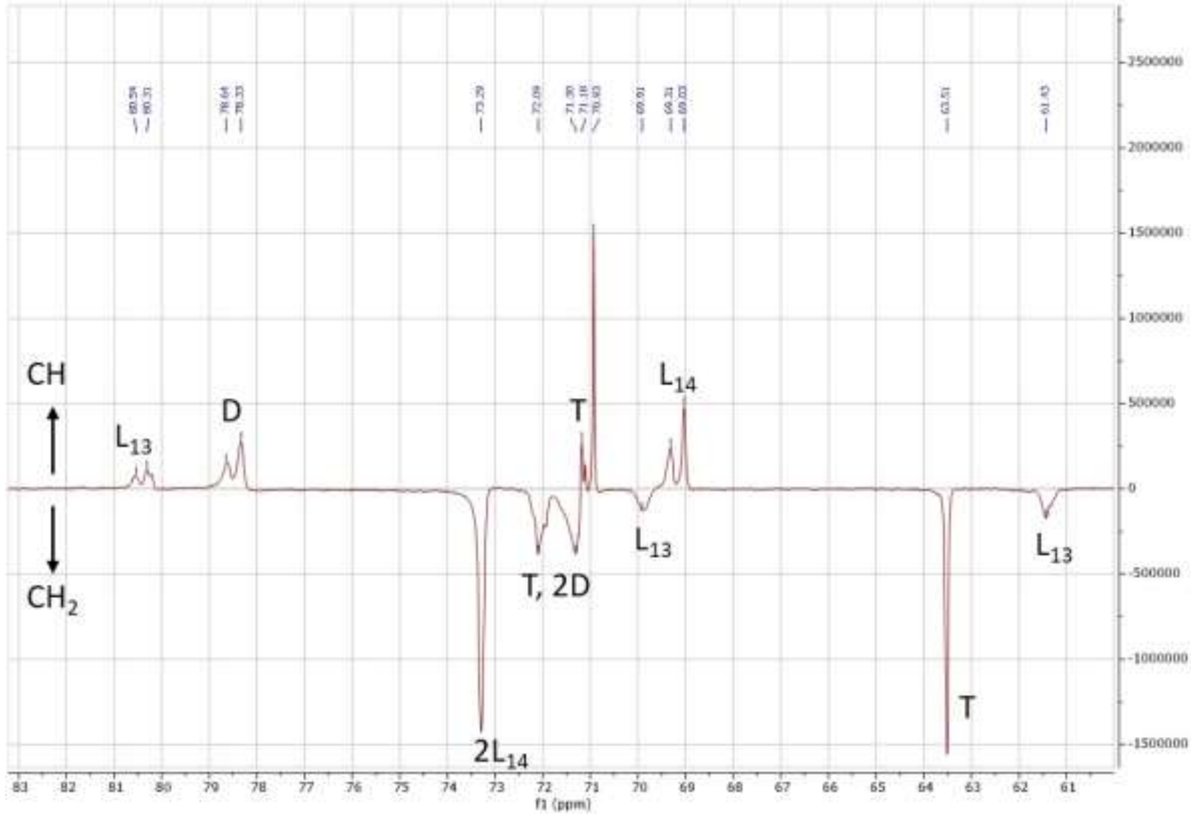

Figure S2. DEPT NMR spectrum of hyperbranched polyglycidol, HbPGL recorded in DMSO-d_6.

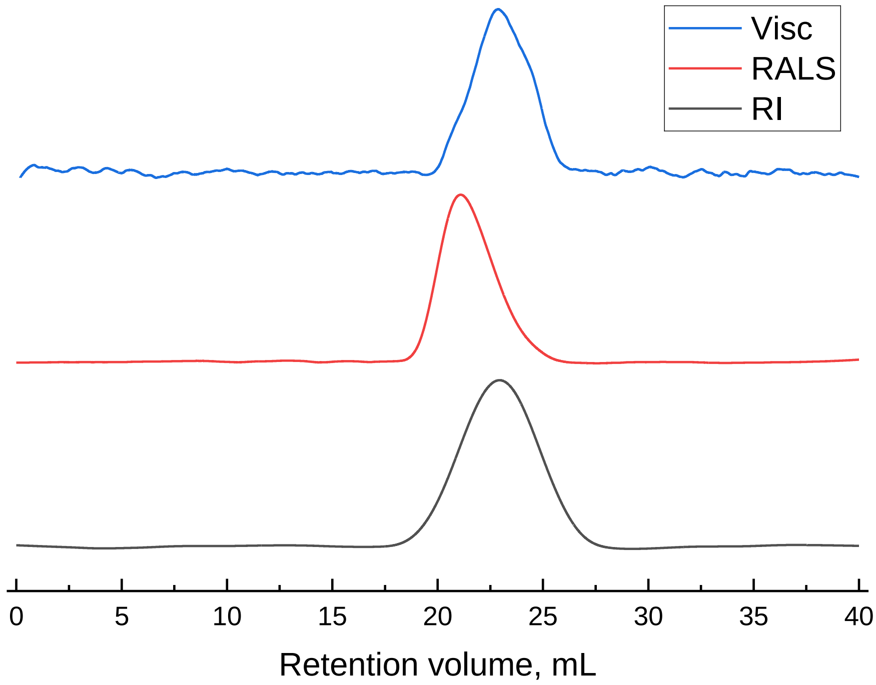

Figure S3. GPC chromatogram of hyperbranched polyglycidol, HbPGL performed in an aqueous solution at a flow rate of 1.0 mL min using a chromatograph (Knauer K-501 HPLC pump) equipped with a degasser (4-Channel Degasser; K-5004, Knauer), three TSK-GEL columns: G5000 PW XL 1 3000 PW XL 1 2500 PW XL (7.8 3 300 mm; Tosho; 26 8C), an LDC RI, RALS and viscometer detectors. Poly(ethylene oxide) was used as a standard.

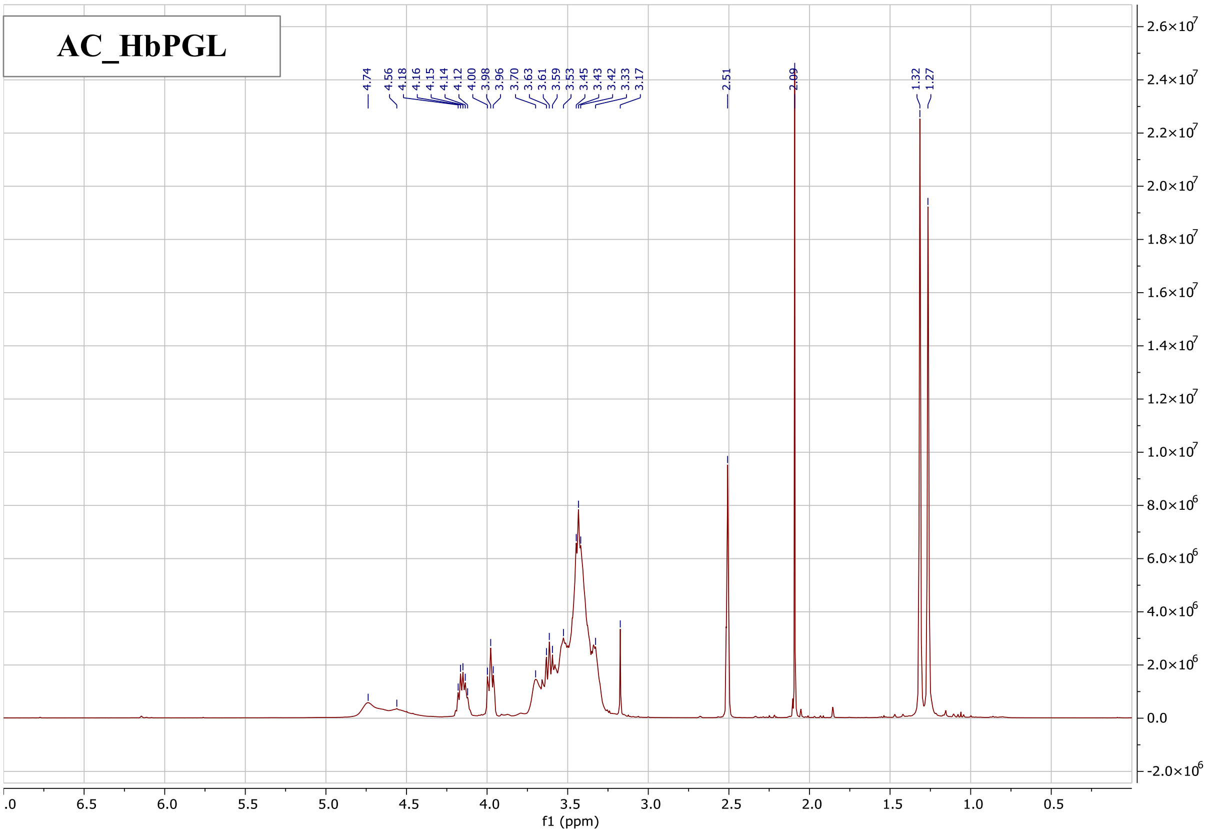

Figure S4. ^{1}H NMR spectrum of AC_HbPGL recorded in DMSO-d_6.

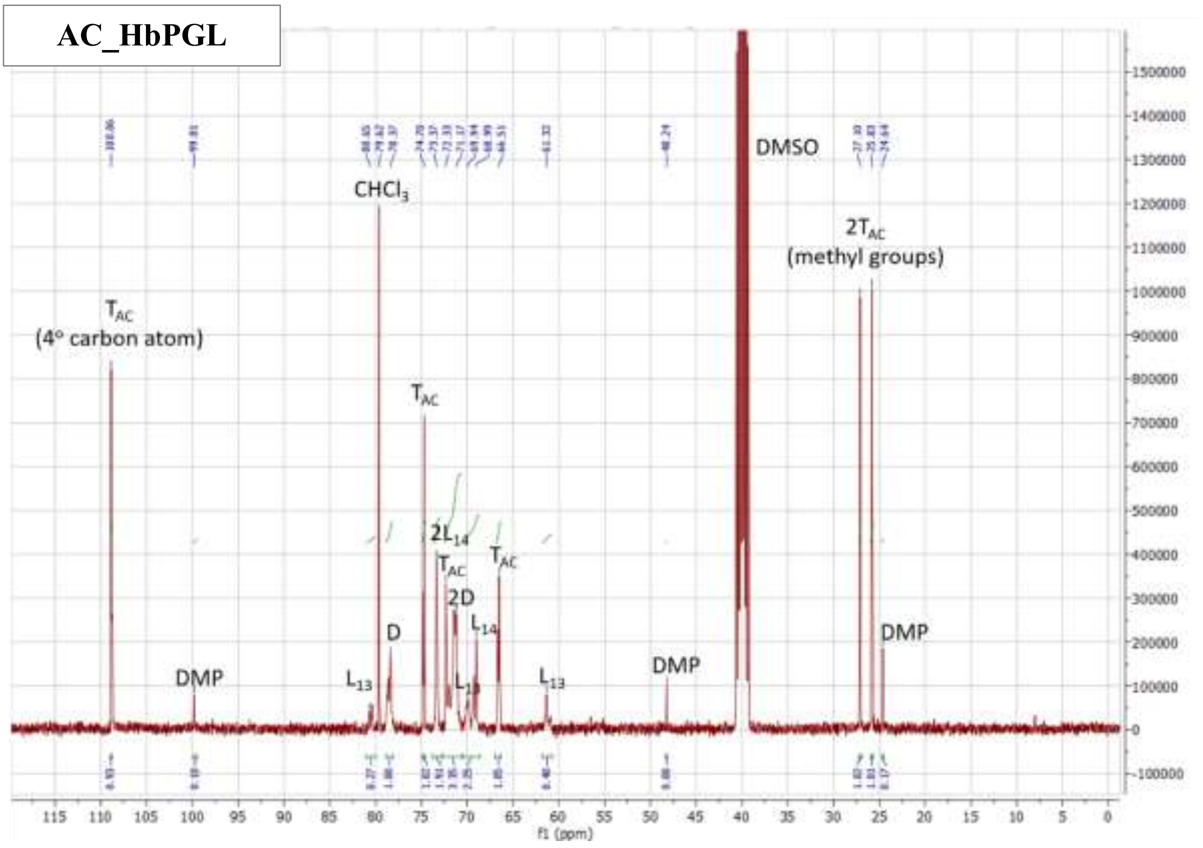

Figure S5. ^{13}C INVGATE NMR spectrum of AC_HbPGL recorded in DMSO-d$_6$. DMP denotes 2,2-dimethoxypropane (contamination after the protection of 1,2-diols).

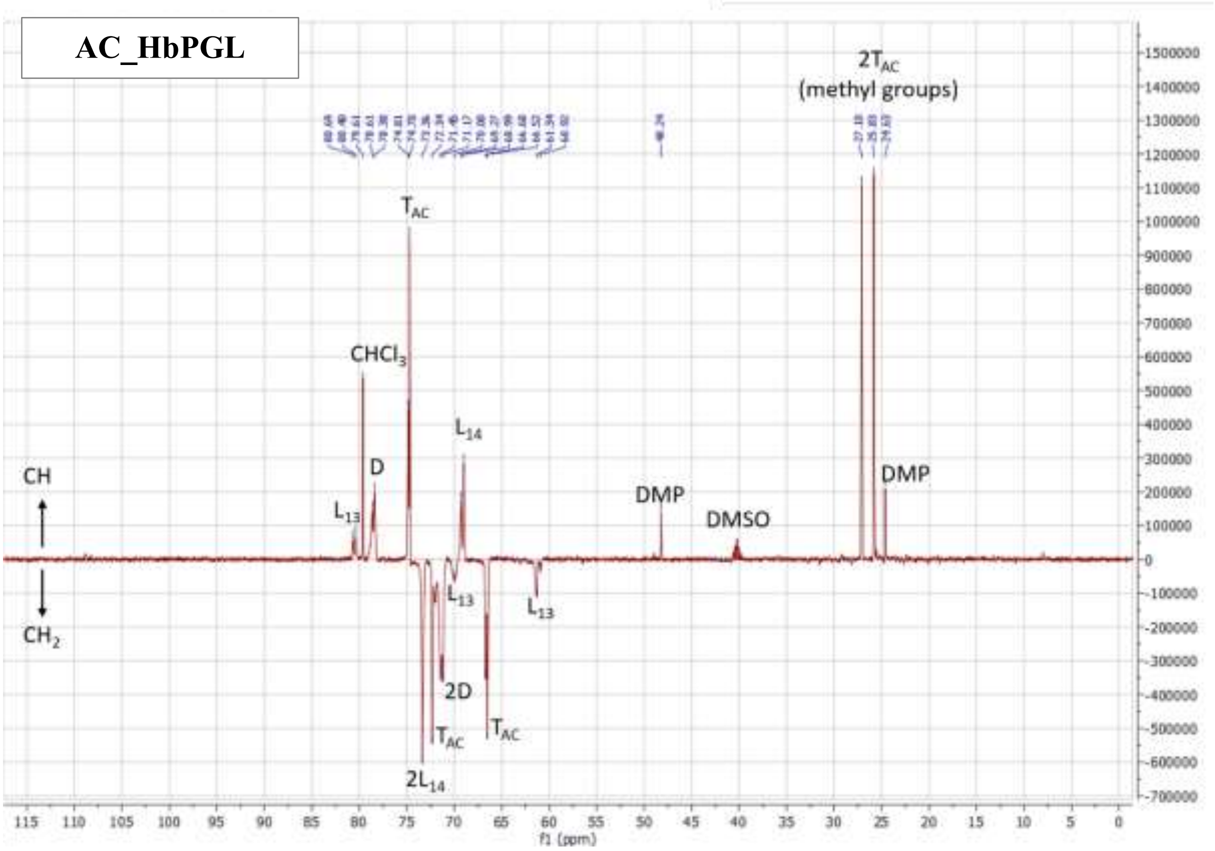

Figure S6. DEPT NMR spectrum of AC_HbPGL recorded in DMSO-d$_6$. DMP denotes 2,2-dimethoxypropane (contamination after the protection of 1,2-diols).

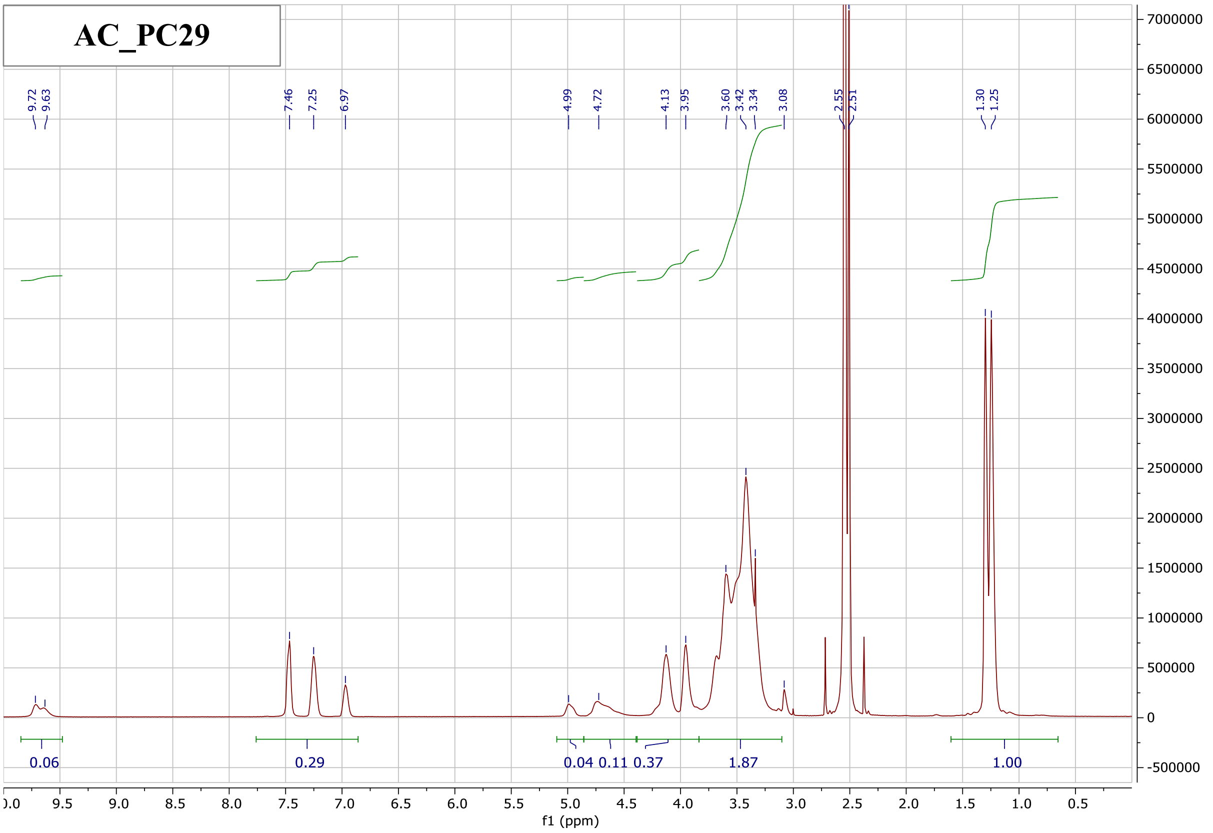

Figure S7. ^{1}H NMR spectrum of AC_PC29 recorded in DMSO-d_6.

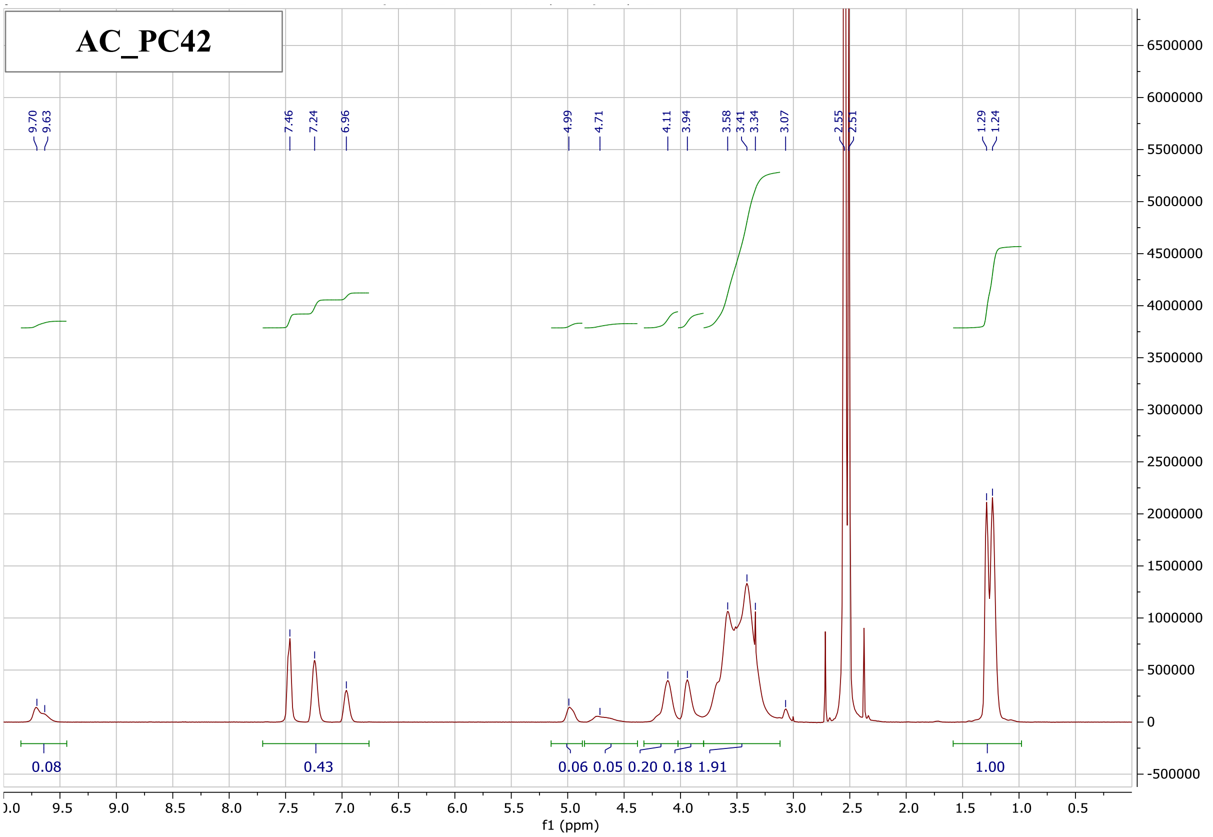

Figure S8. ^{1}H NMR spectrum of AC_PC42 recorded in DMSO-d_6.

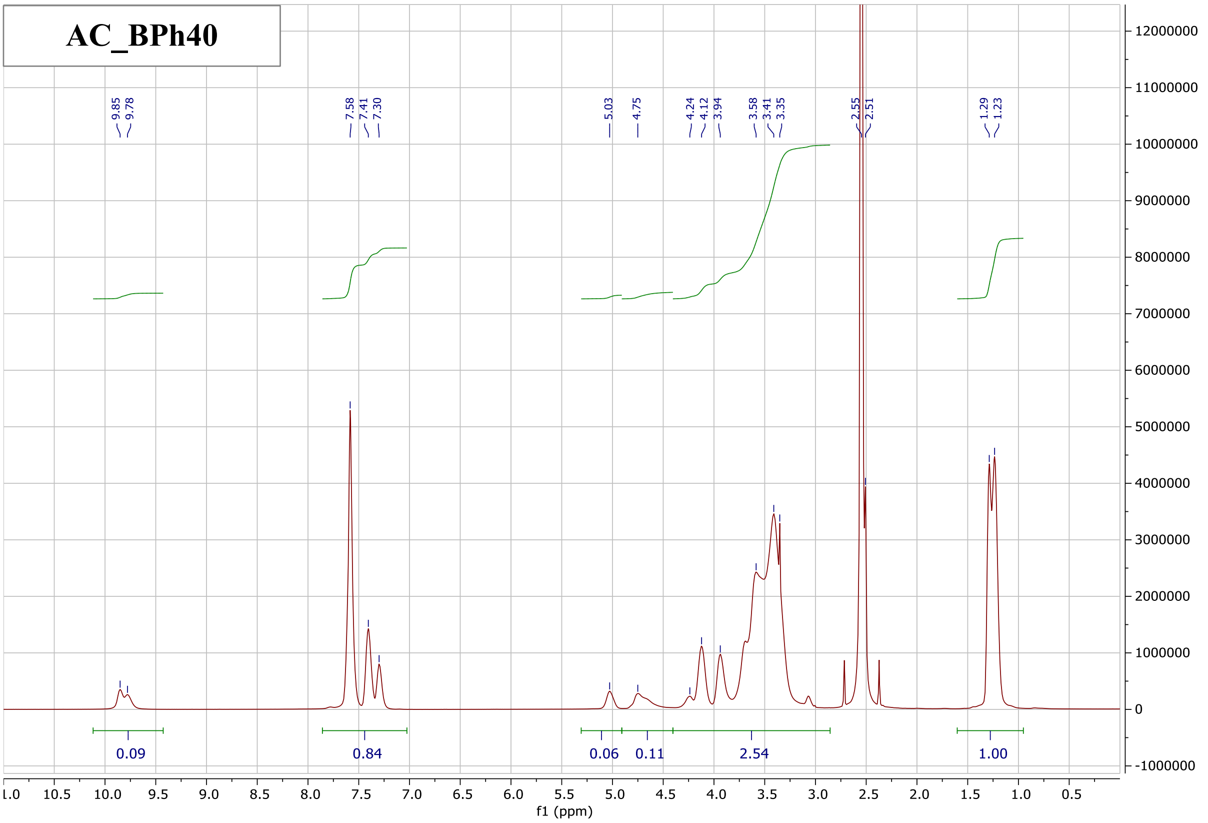

Figure S9. ^{1}H NMR spectrum of AC_BPh40 recorded in DMSO-d_6.

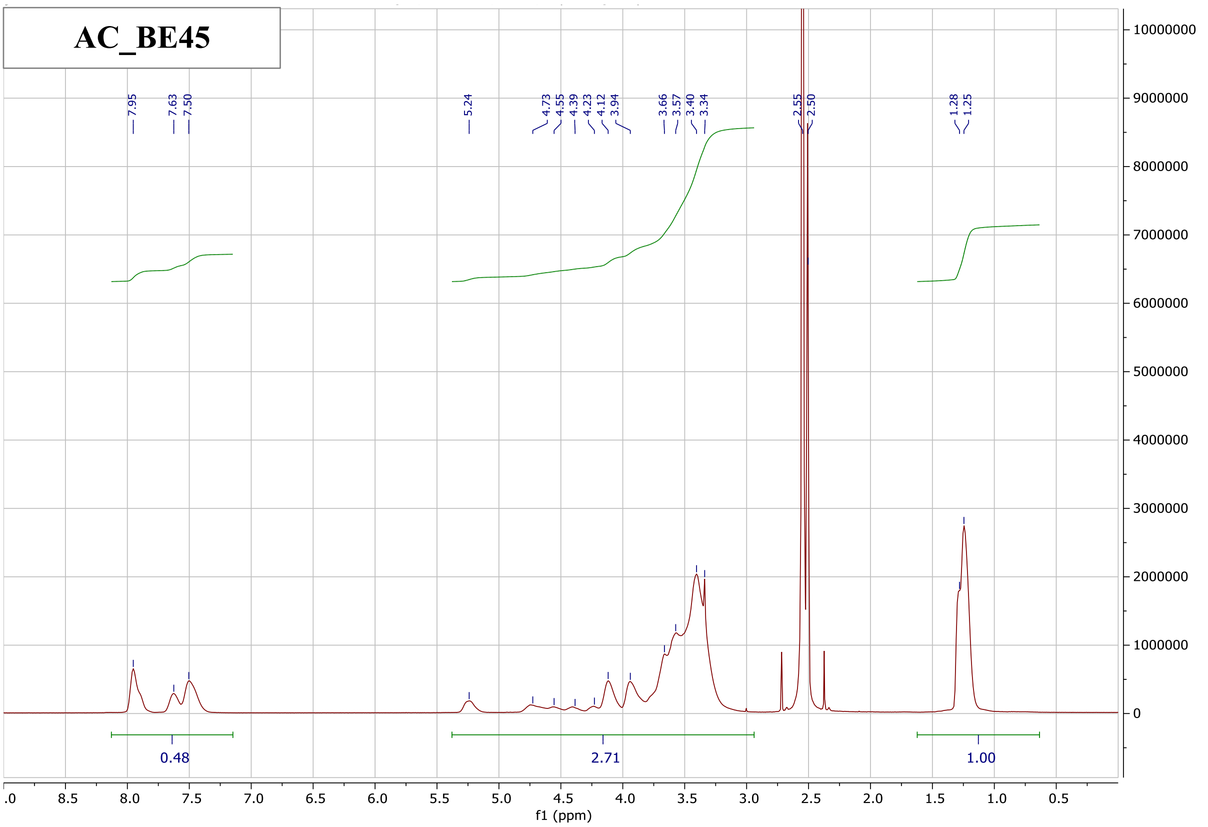

Figure S10. ^{1}H NMR spectrum of AC_BE45 recorded in DMSO-d$_6$.

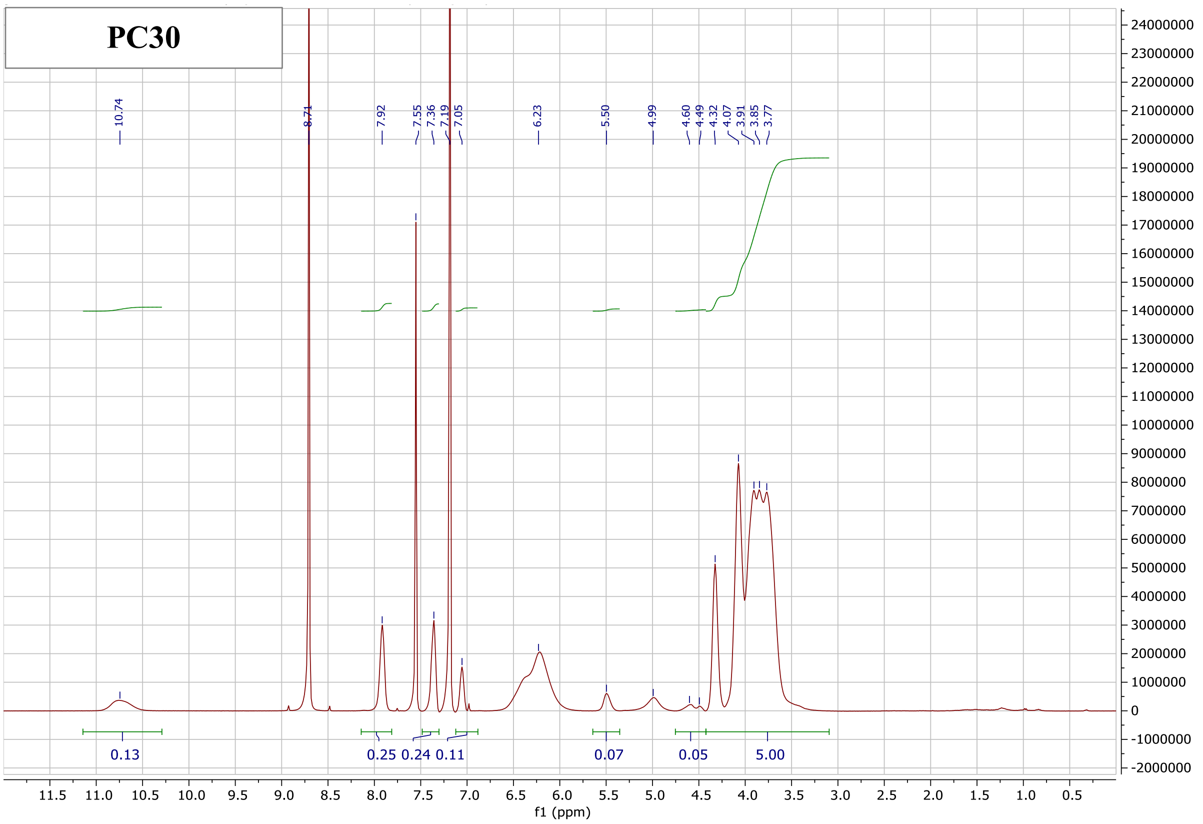

Figure S11. ^{1}H NMR spectrum of PC30 recorded in pyridine-d$_5$.

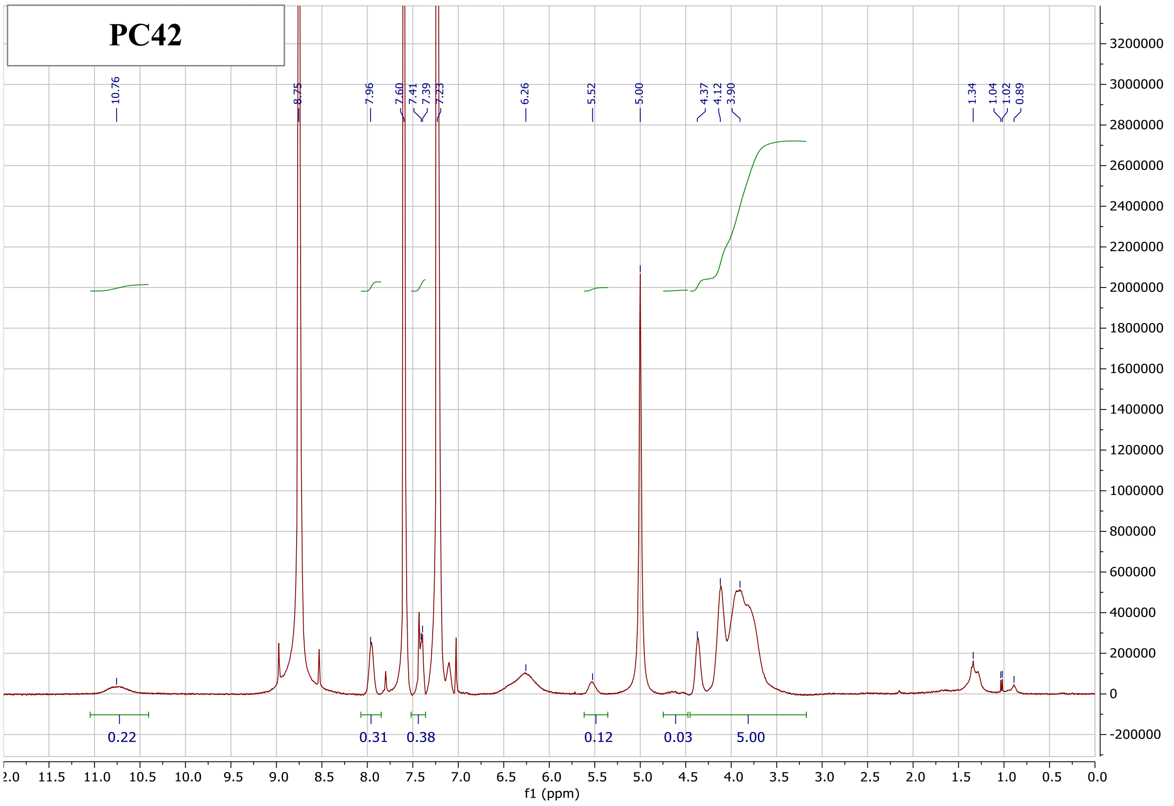

Figure S12. ^{1}H NMR spectrum of PC42 recorded in pyridine-d_5.

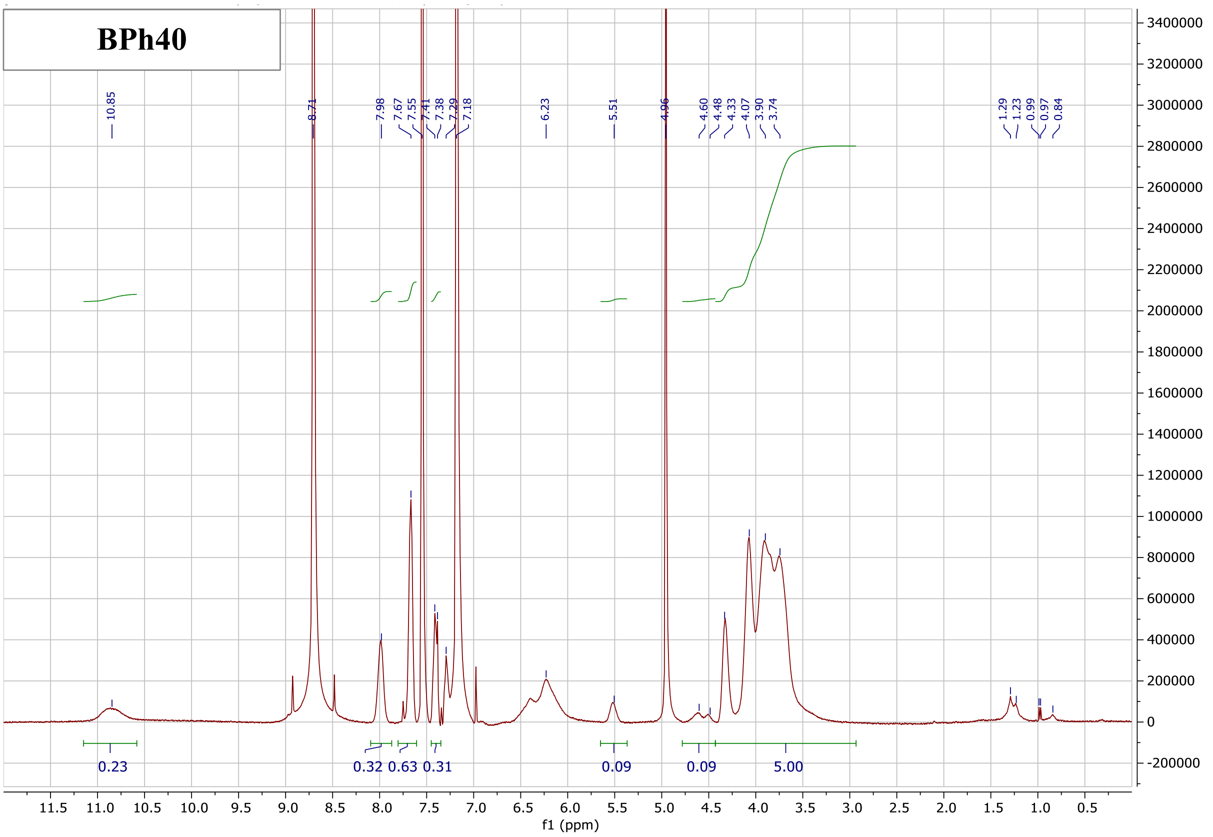

Figure S13. ^{1}H NMR spectrum of HbPGL_BPh40 recorded in pyridine-d_5.

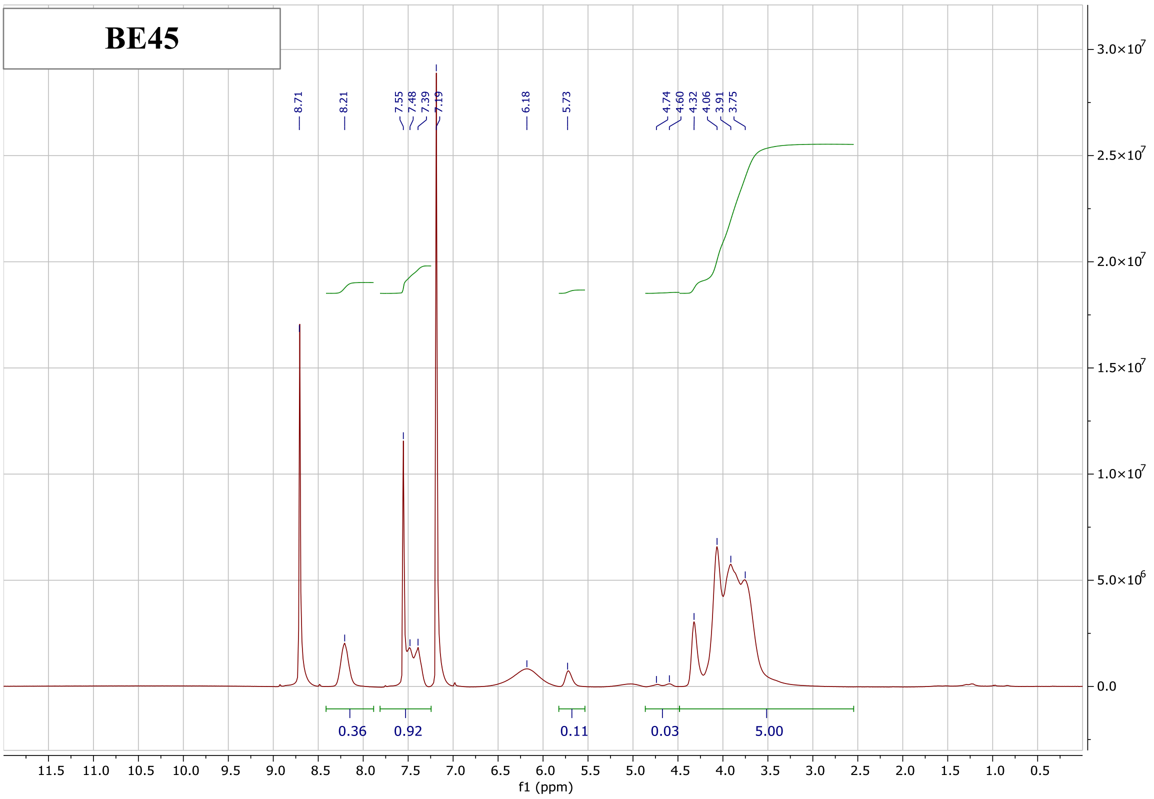

Figure S14. ^{1}H NMR spectrum of HbPGL_BE45 recorded in pyridine-d_5.

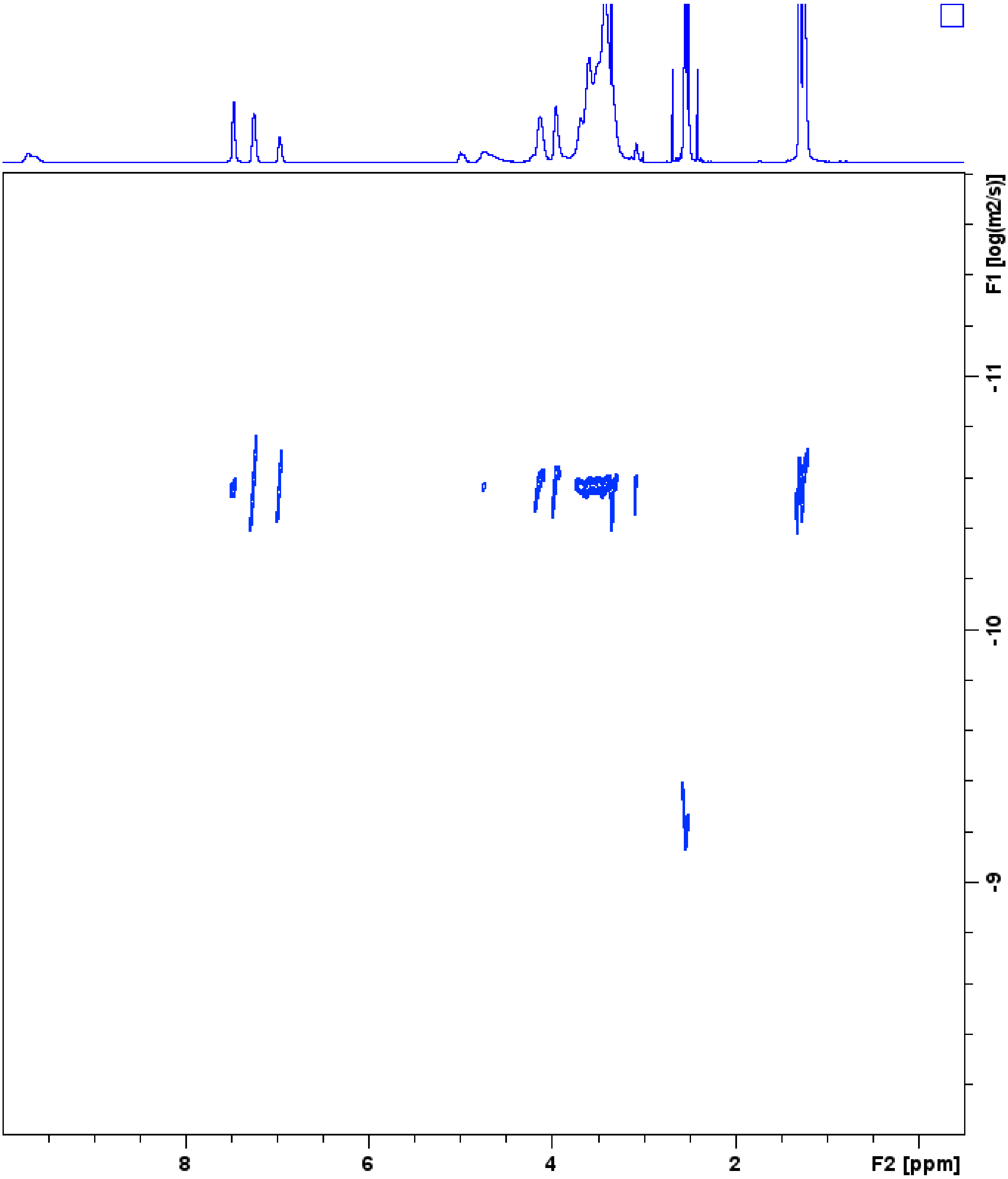

Figure S15. ^{1}H DOSY NMR spectrum of AC_PC30 recorded in DMSO-d_6.

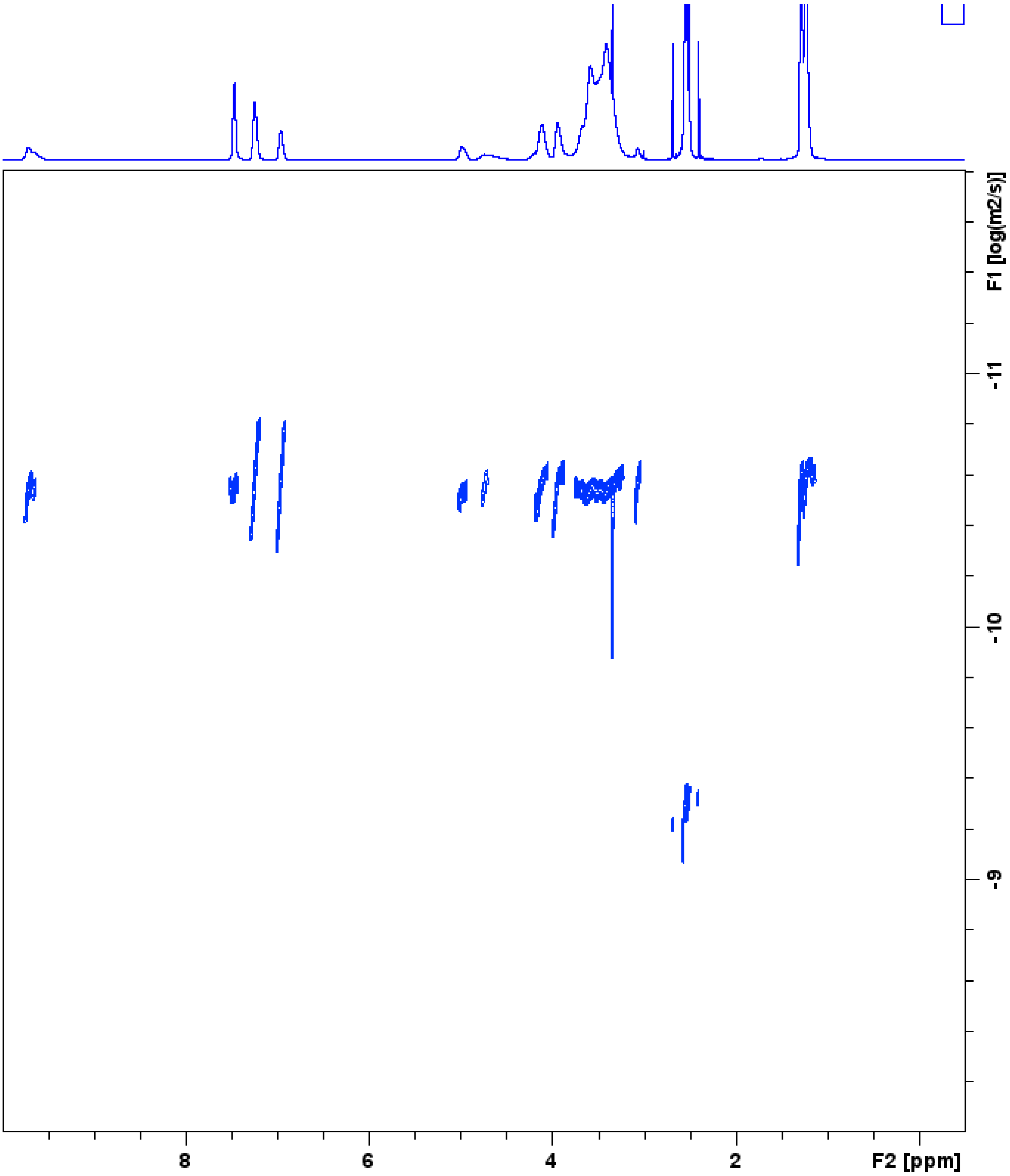

Figure S16. ^{1}H DOSY NMR spectrum of AC_PC42 recorded in DMSO-d_6.

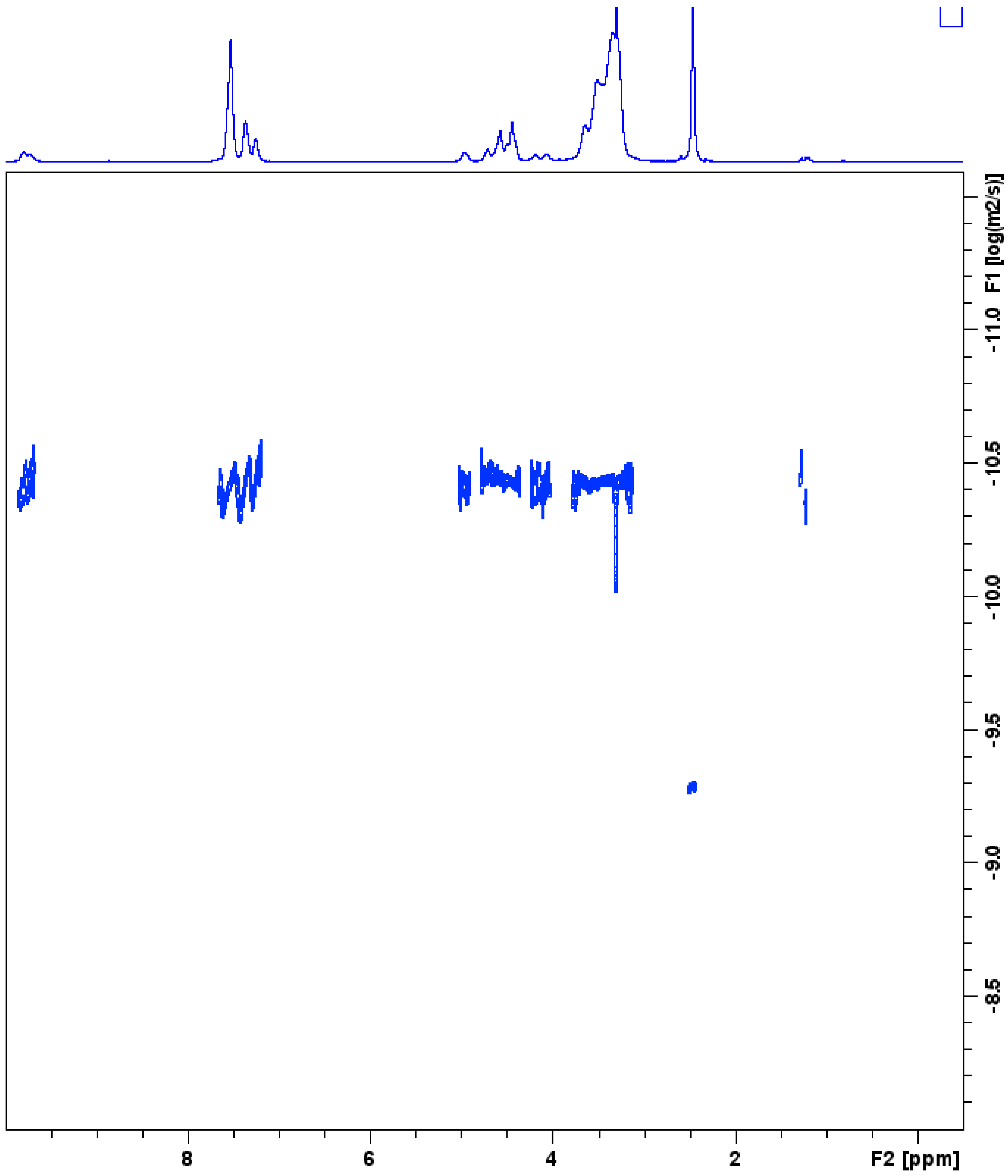

Figure S17. ^{1}H DOSY NMR spectrum of BPh40 recorded in DMSO-d_6.

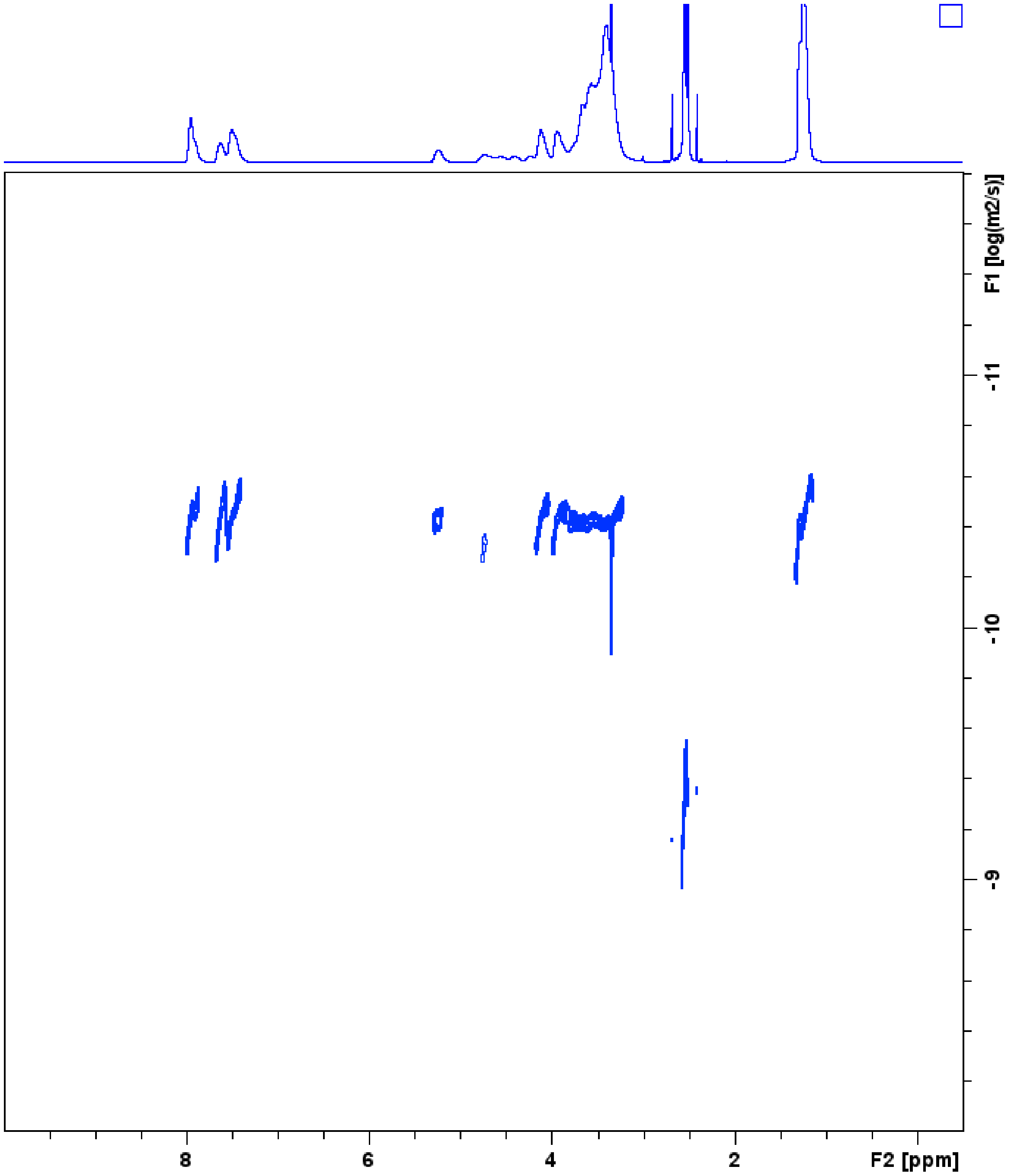

Figure S18. ^{1}H DOSY NMR spectrum of AC_BE45 recorded in DMSO-d_6

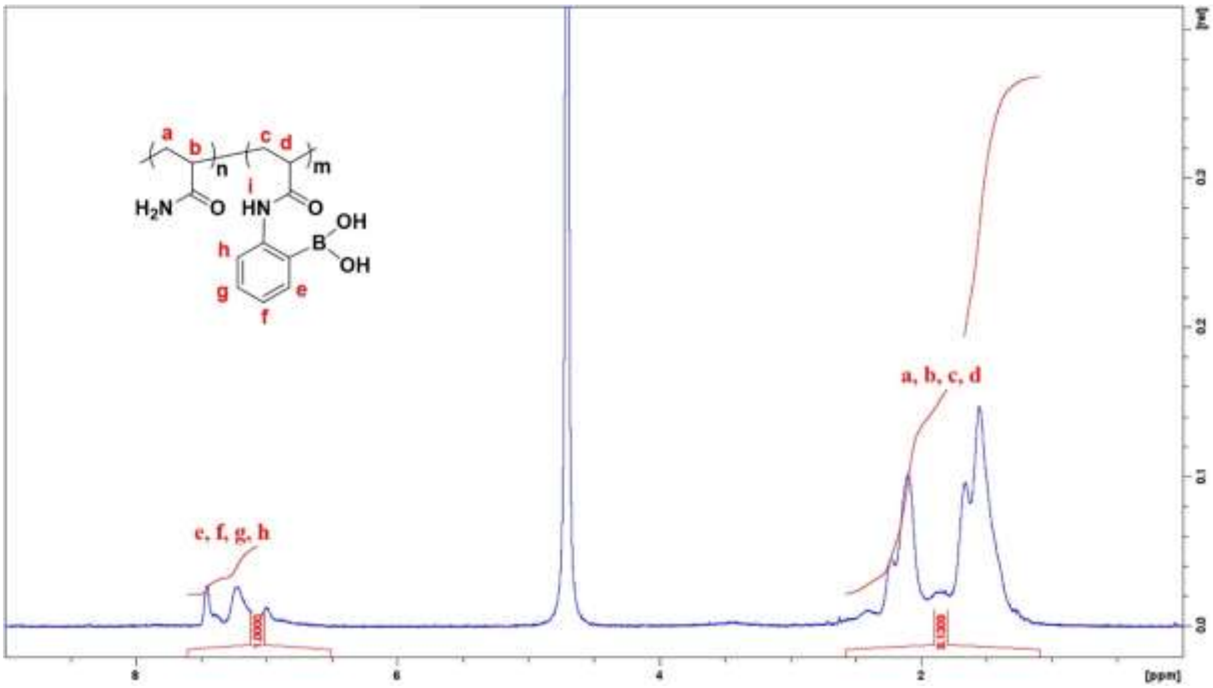

Figure S19. ^{1}H NMR spectrum of poly(AM-*ran*-2-AAPBA)_1 recorded in D_2O.

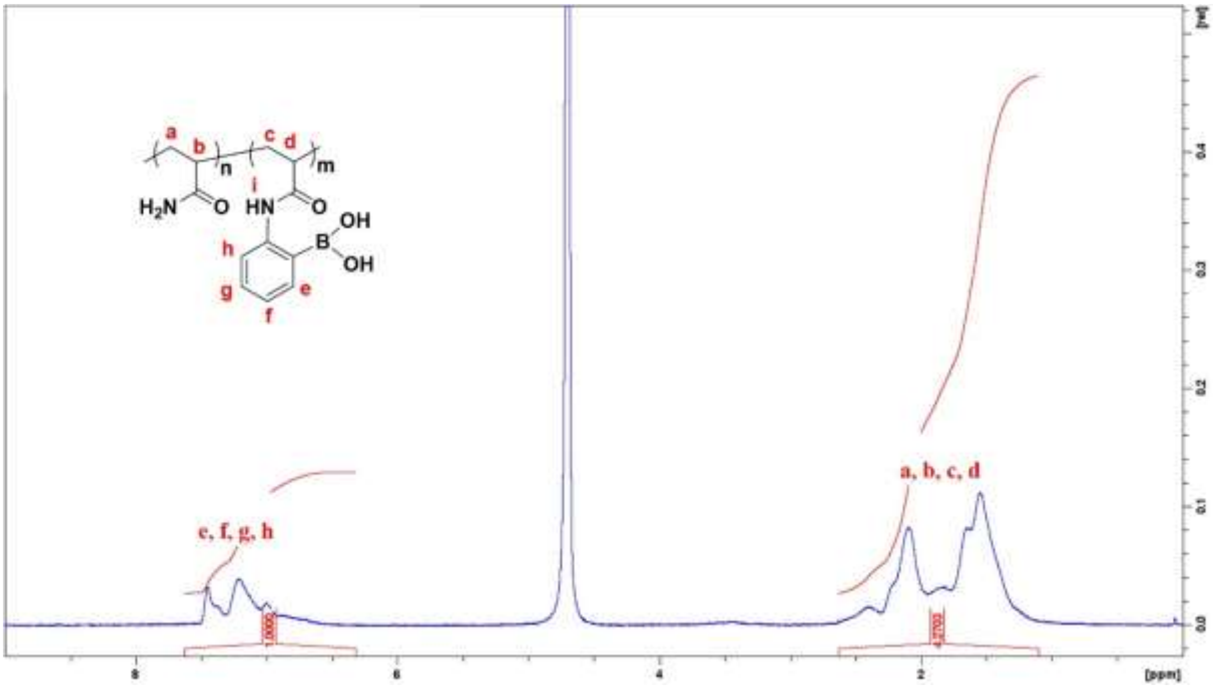

Figure S20. ^{1}H NMR spectrum of poly(AM-*ran*-2-AAPBA)_2 recorded in D_2O.

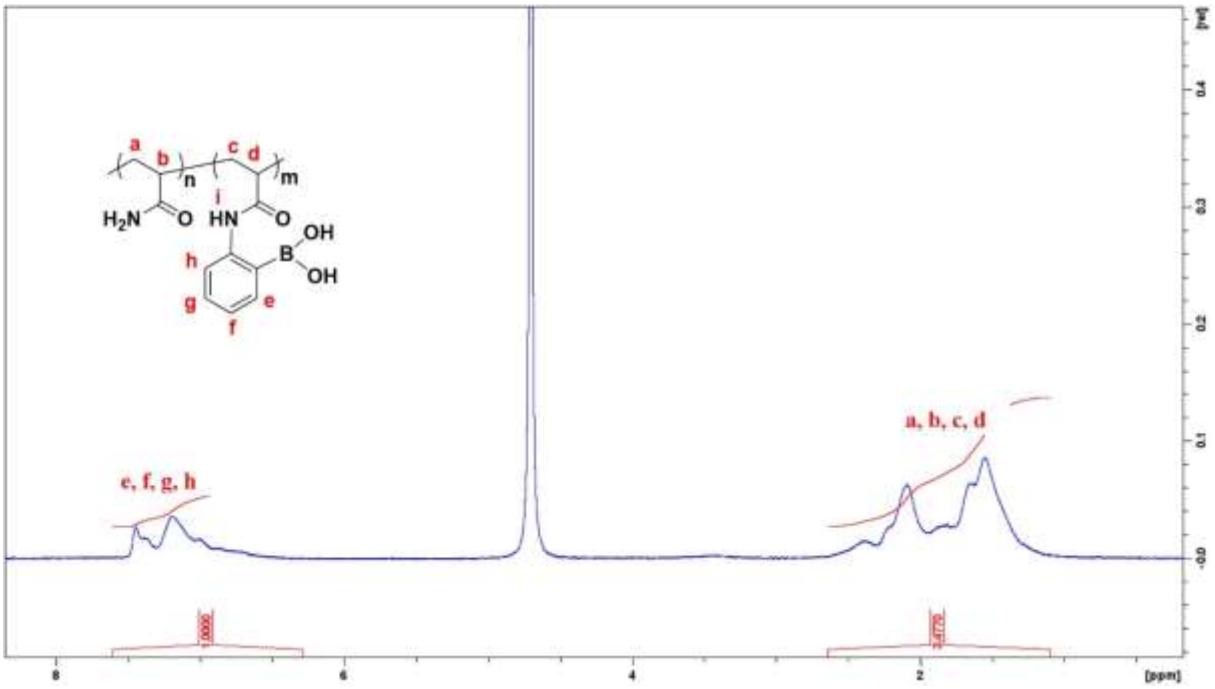

Figure S21. ^{1}H NMR spectrum of poly(AM-*ran*-2-AAPBA)_3 recorded in D_2O.

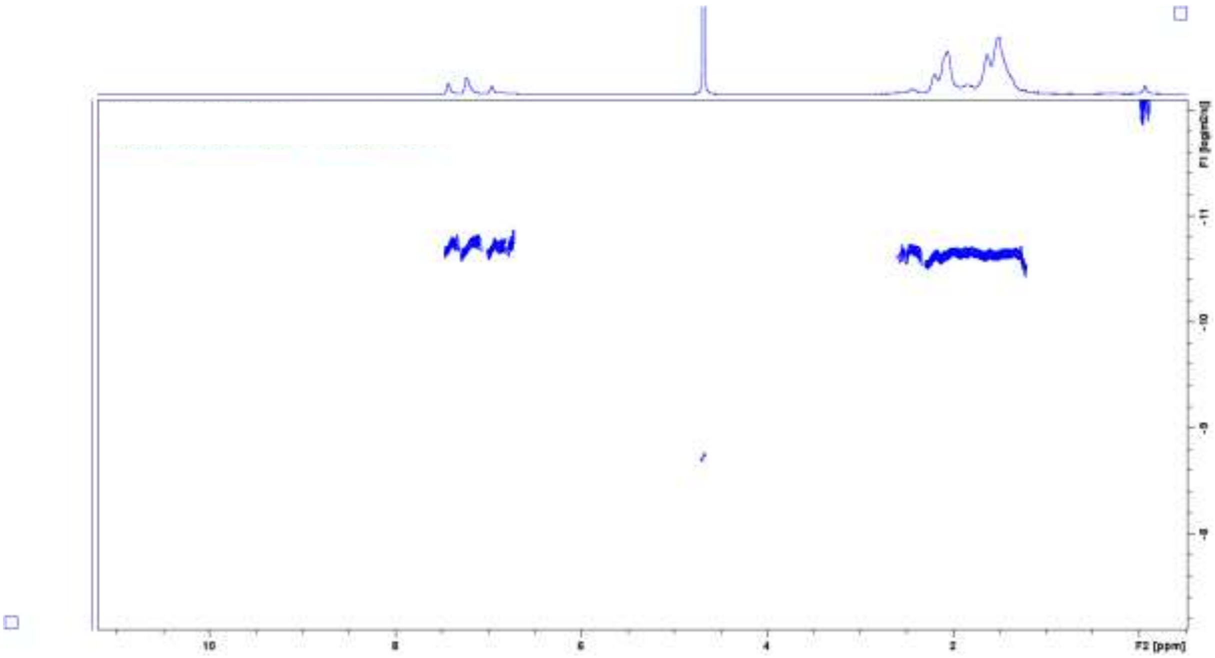

Figure S22. ^{1}H DOSY NMR spectrum of poly(AM-*ran*-2-AAPBA)_1 recorded in D_2O.

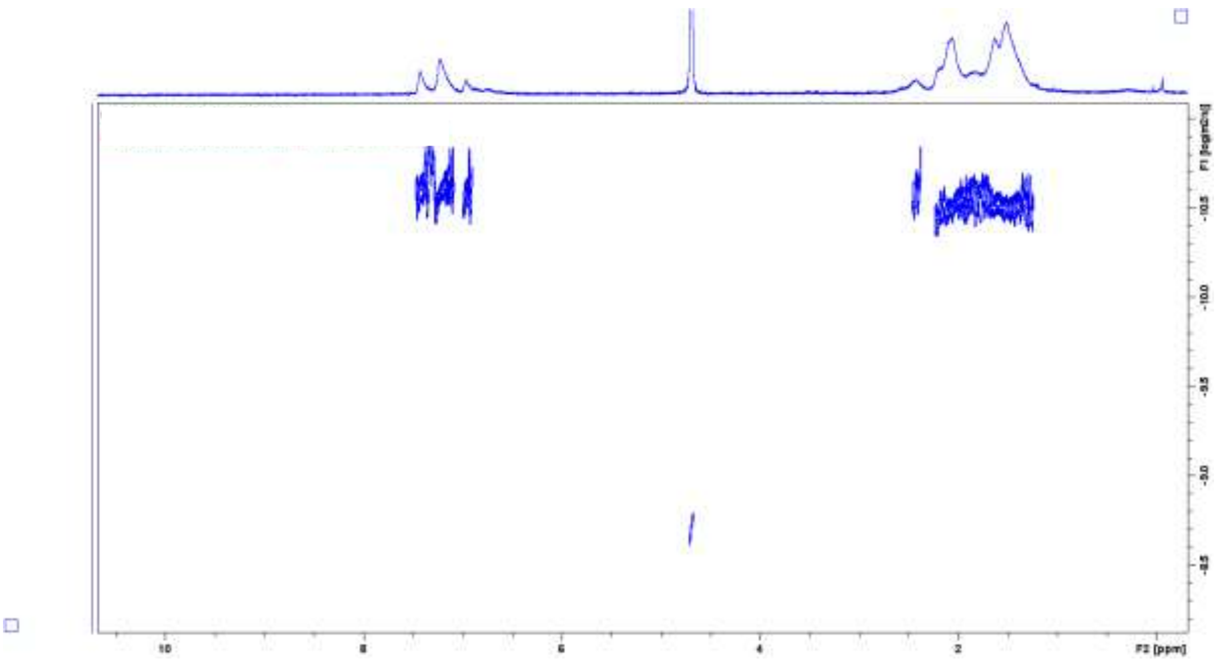

Figure S23. ^{1}H DOSY NMR spectrum of poly(AM-*ran*-2-AAPBA)_2 recorded in D_2O.

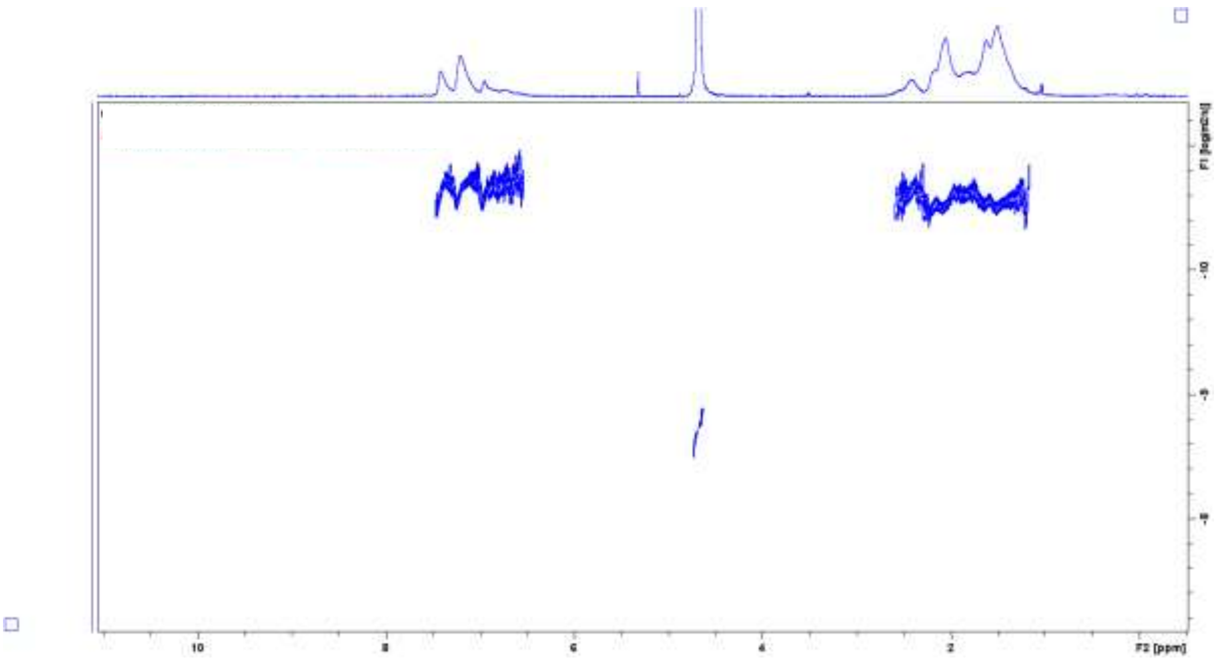

Figure S24. ^{1}H DOSY NMR spectrum of poly(AM-*ran*-2-AAPBA)_3 recorded in D_2O.

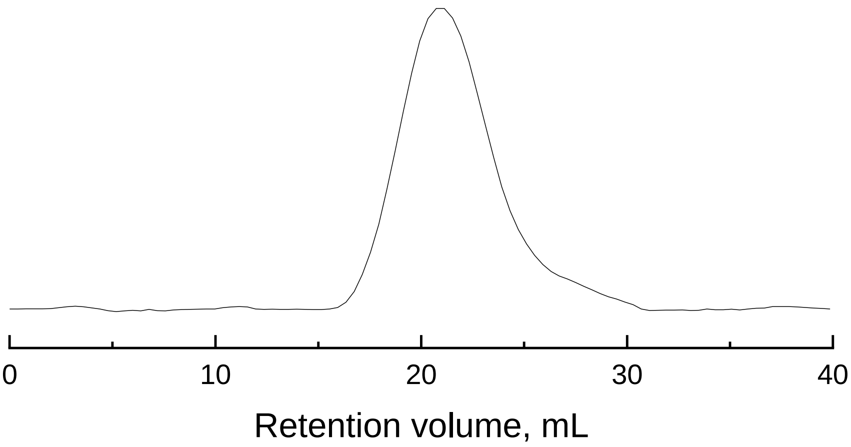

Figure S25. GPC chromatogram of poly(AM-*ran*-2-AAPBA)_1 performed in an aqueous solution at a flow rate of 1.0 mL min using a chromatograph (Knauer K-501 HPLC pump) equipped with a degasser (4-Channel Degasser; K-5004, Knauer), three TSK-GEL columns: G5000 PW XL 1 3000 PW XL 1 2500 PW XL (7.8 3 300 mm; Tosho; 26 8C), an LDC RI detector. Poly(ethylene oxide) was used as a standard.

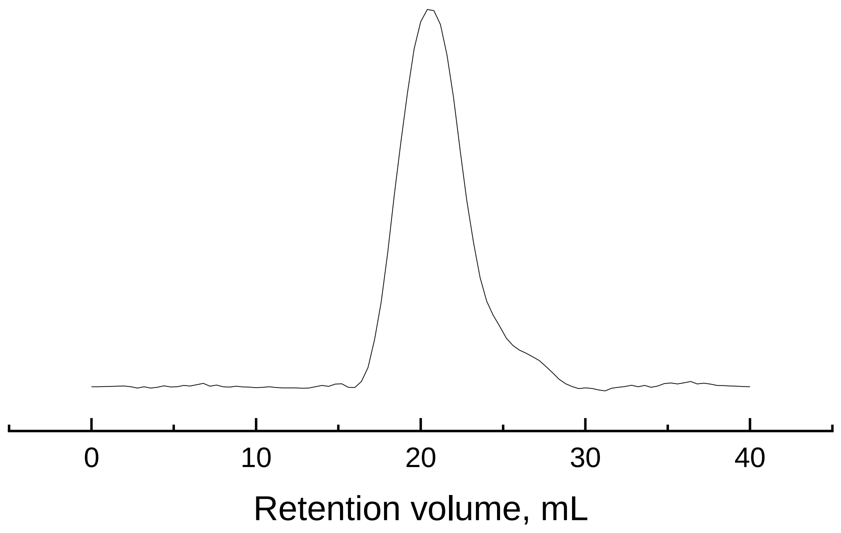

Figure S26. GPC chromatogram of poly(AM-*ran*-2-AAPBA)_2 performed in an aqueous solution at a flow rate of 1.0 mL min using a chromatograph (Knauer K-501 HPLC pump) equipped with a degasser (4-Channel Degasser; K-5004, Knauer), three TSK-GEL columns: G5000 PW XL 1 3000 PW XL 1 2500 PW XL (7.8 3 300 mm; Tosho; 26 8C), an LDC RI detector. Poly(ethylene oxide) was used as a standard.

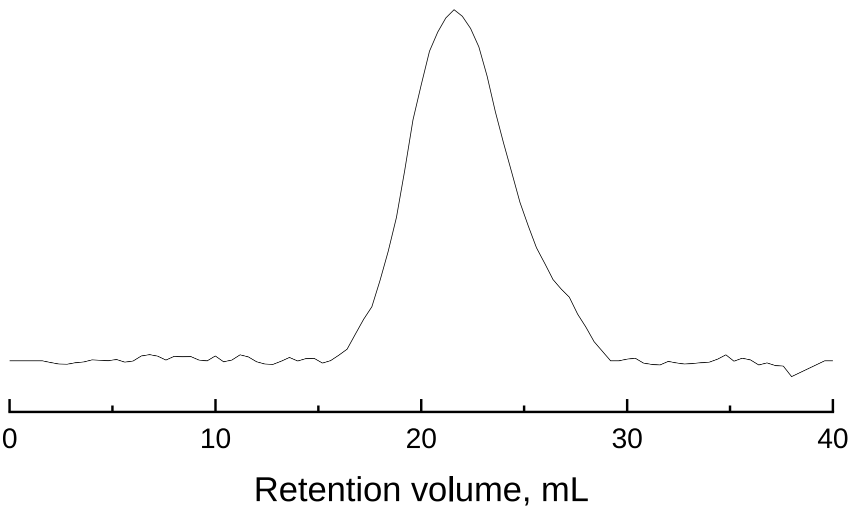

Figure S27. GPC chromatogram of poly(AM-*ran*-2-AAPBA)_3 performed in an aqueous solution at a flow rate of 1.0 mL min using a chromatograph (Knauer K-501 HPLC pump) equipped with a degasser (4-Channel Degasser; K-5004, Knauer), three TSK-GEL columns: G5000 PW XL 1 3000 PW XL 1 2500 PW XL (7.8 3 300 mm; Tosho; 26 8C), an LDC RI detector. Poly(ethylene oxide) was used as a standard.

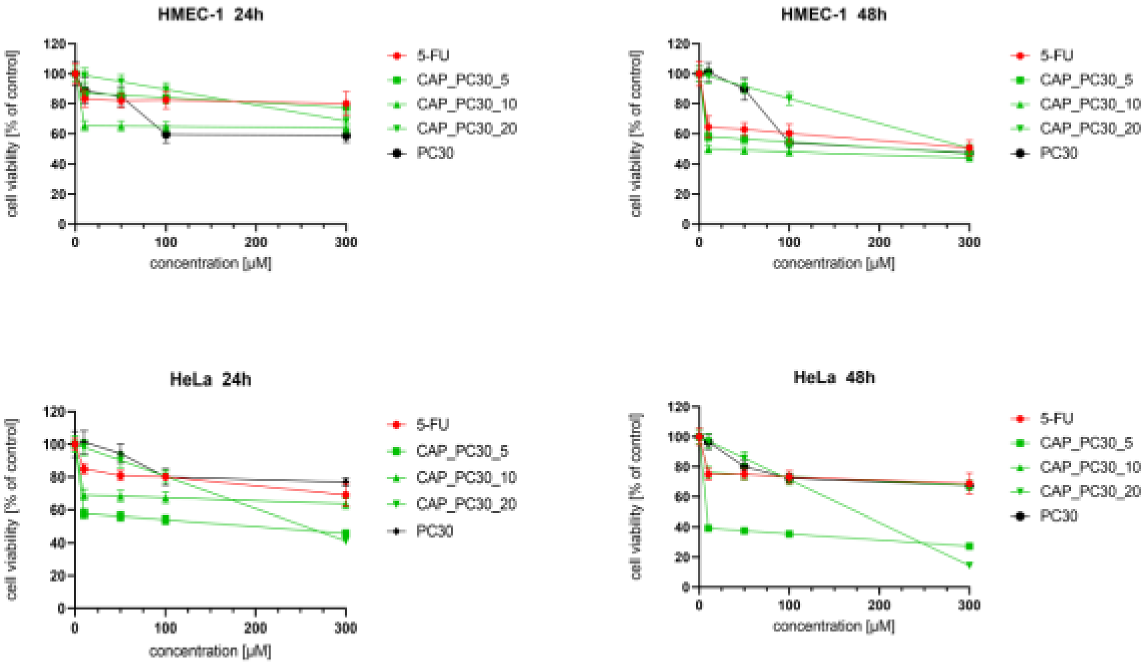

Figure S28. Influence of neat PC30 polymer, free 5-fluorouracil, and 5-fluorouracil-loaded PC30 polymer on HeLa (upper panel) and HMEC1 (lower panel) cells viability after 24- and 48-hour incubation. Results are expressed as mean ± SD (n = 16).

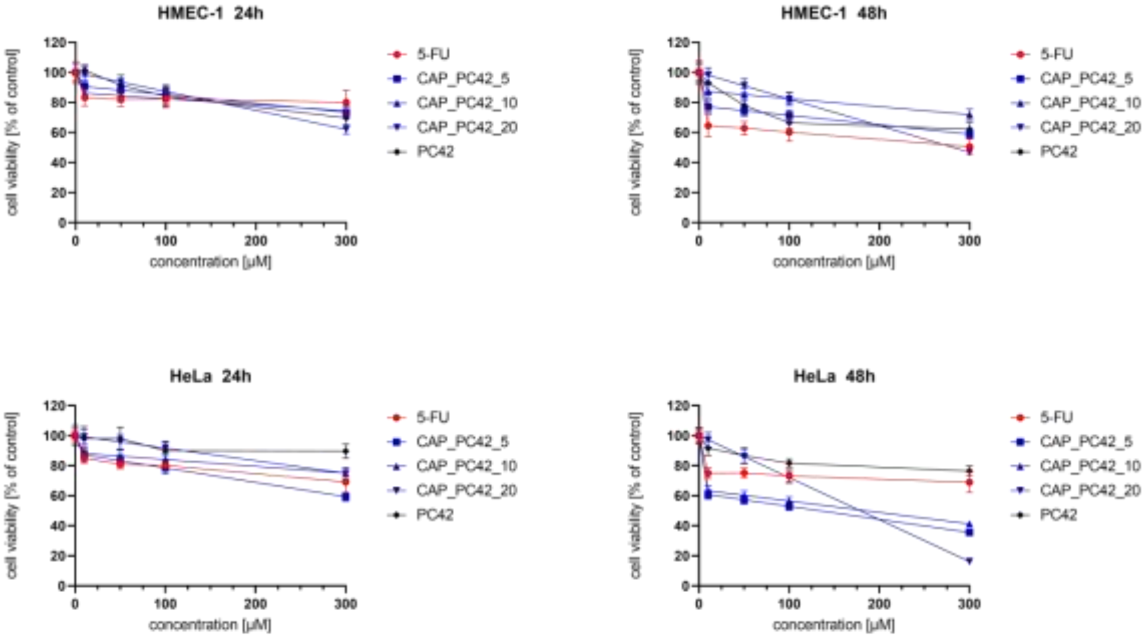

Figure S29. Influence of neat PC42 polymer, free 5-fluorouracil, and 5-fluorouracil-loaded PC42 polymer on HeLa (upper panel) and HMEC1 (lower panel) cells viability after 24- and 48-hour incubation. Results are expressed as mean ± SD (n = 16).

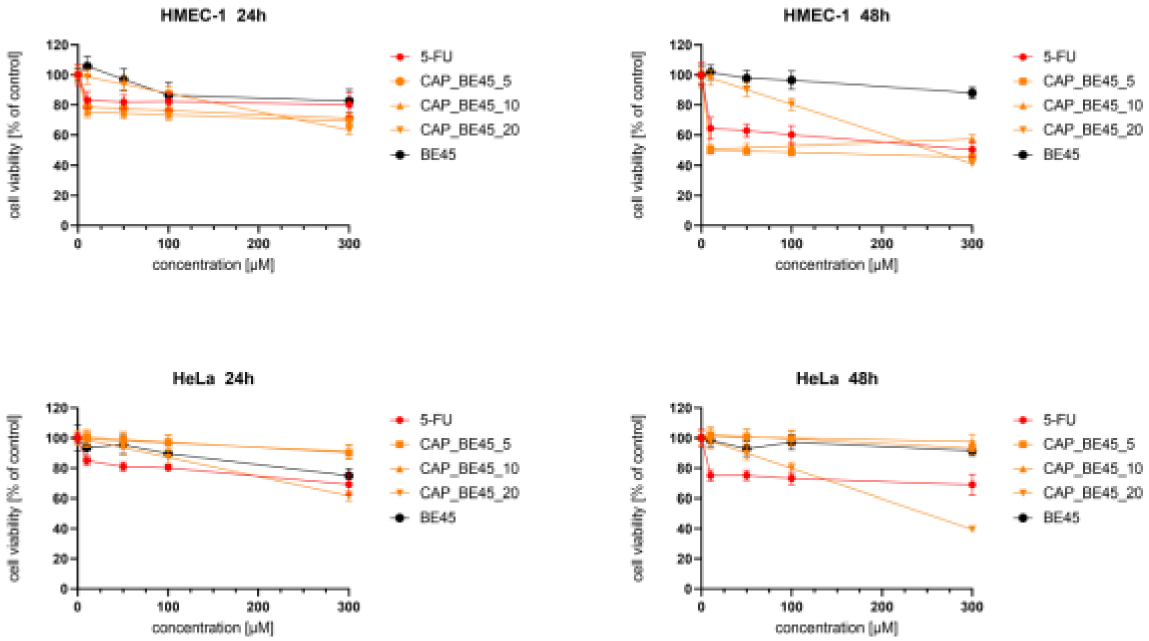

Figure S30. Influence of neat BE54 polymer, free 5-fluorouracil, and 5-fluorouracil-loaded BE54 polymer on HeLa (upper panel) and HMEC1 (lower panel) cells viability after 24- and 48-hour incubation. Results are expressed as mean ± SD (n = 16).

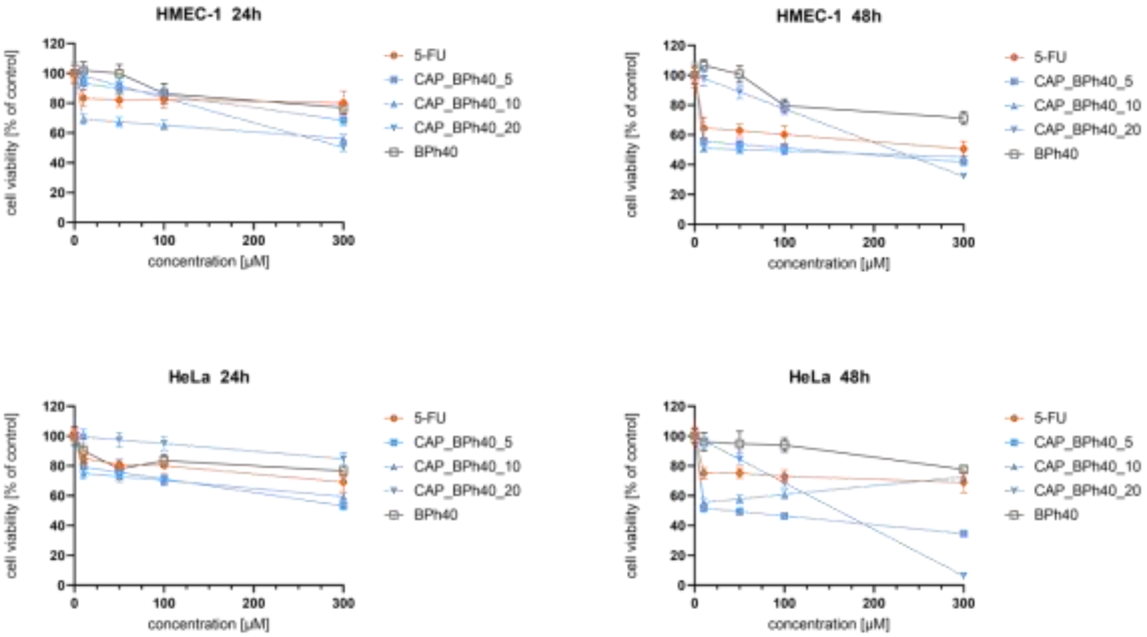

Figure S31. Influence of neat BPh40 polymer, free 5-fluorouracil, and 5-fluorouracil-loaded BPh40 polymer on HeLa (upper panel) and HMEC1 (lower panel) cells viability after 24- and 48-hour incubation. Results are expressed as mean ± SD (n = 16).

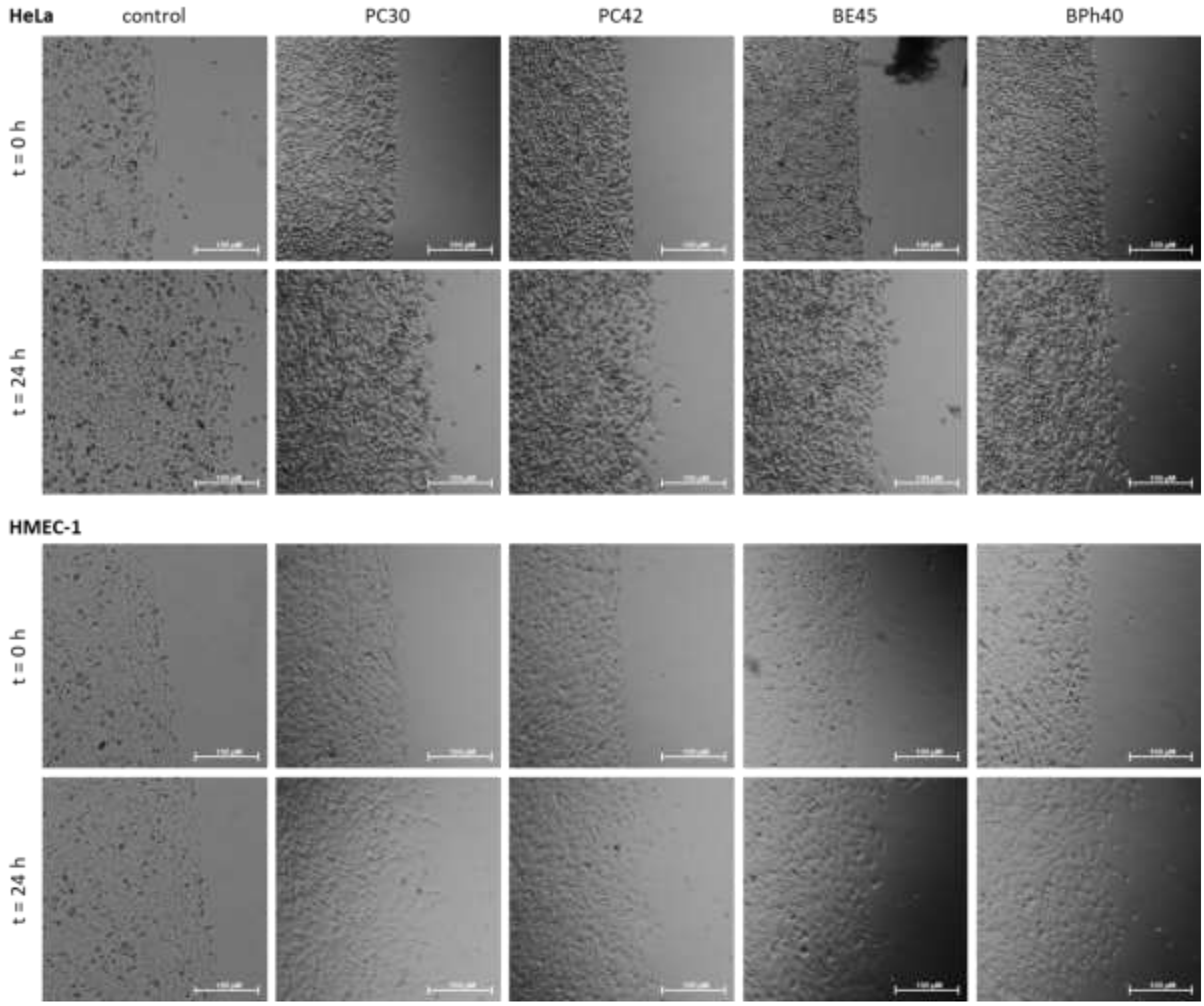

Figure S32. Influence of the pure gels based on PC30, PC42, BE45 and BPh40 polymers on HeLa (upper panel) and HMEC-1 (bottom panel) cell viability immediately after gel administration and after 24 h of incubation. Microscope magnification 4×, bar 100 µm.

To facilitate the evaluation of the microscopic images (main manuscript Figure 5-8 and Supplementary Figure S1), we decided to use the ImageJ program[S1] to assess the area with the cells and determine its increase or decrease caused by the action of the tested gels. Data showing the effect of the tested gels on the viability of HeLa cancer cells and non-cancerous HMEC-1 cells, immediately after gel application and after 24 hours of incubation, are presented in Table S1.

Table S1. Effect of neat hydrogels and drug-loaded hydrogels on the viability of HeLa cells assayed immediately after gel administration and after 24 hours of incubation.

	area with cells	total area	% of area with cells relative to total area
control t = 0 h	42624.00±18.37	87616.00±13.92	48.65±0.04
control t = 24 h	70027.00±14.04	87024.00±10.90	80.47±0.03
pure gel PC30 t = 0 h	46452.00±32.70	87024.00±28.77	53.38±0.07
pure gel PC30 t = 24 h	59295.00±32.24	87025.00±36.21	68.14±0.07
CAP_PC30_5 t = 0 h	44104.00±16.36	87616.00±12.79	50.34±0.03
CAP_PC30_ 5 t = 24 h	48248.00±20.58	87616.00±19.36	55.07±0.05
CAP_PC30_10 t = 0 h	44545.00±20.15	87320.00±15.95	51.01±0.04
CAP_PC30_10 t = 24 h	492.00±35.04	87616.00±15.62	0.56±0.06
CAP_PC30_20 t = 0 h	43365.00±25.60	87025.00±19.94	49.83±0.05
CAP_PC30_20 t = 24 h	1274.00±12.54	87024.00±12.05	1.46±0.03
pure gel PC42 t = 0 h	44992.00±33.07	87616.00±34.81	51.35±0.07
pure gel PC42 t = 24 h	53280.00±30.48	87616.00±34.57	60.81±0.07
CAP_PC42 5 t = 0 h	43512.00±15.99	87616.00±12.15	49.66±0.03
CAP_PC42_5 t = 24 h	46768.00±20.57	87320.00±12.17	53.56±0.04
CAP_PC42_10 t = 0 h	48345.00±18.51	86435.00±14.96	55.93±0.04
CAP_PC42_10 t = 24 h	23934.00±24.55	87320.00±12.17	27.41±0.04
CAP_PC42_20 t = 0 h	39664.00±27.74	87616.00±12.15	45.27±0.05
CAP_PC42_20 t = 24 h	504.00±25.86	86728.00±24.34	0.58±0.06
pure gel BE45 t = 0 h	44840.00±25.71	87025.00±23.37	51.53±0.07
pure gel BE45 t = 24 h	52096.00±32.74	87320.00±36.56	59.66±0.07
CAP_BE45_5 t = 0 h	44982.00±17.51	87024.00±13.39	51.69±0.04

CAP_BE45_5 t = 24 h	50.00±25.24	87616.00±14.91	0.06±0.05
CAP_BE45_10 t = 0 h	44250.00±18.52	87025.00±14.23	50.85±0.04
CAP_BE45_10 t = 24 h	63.00±23.32	87320.00±13.38	0.07±0.04
CAP_BE45_20 t = 0 h	47200.00±32.12	86730.00±26.71	54.42±0.07
CAP_BE45_20 t = 24 h	2760.00±29.86	86435.00±26.85	3.19±0.07
pure gel BPh40t = 0 h	46768.00±31.83	87320.00±35.03	53.56±0.07
pure gel BPh40 t = 24 h	48380.00±31.49	86730.00±32.83	55.78±0.07
CAP_BPh40 5 t = 0 h	45584.00±14.79	87616.00±11.67	52.03±0.03
CAP_BPh40_5 t = 24 h	55056.00±20.43	87616.00±17.99	62.84±0.05
CAP_BPh40_10 t = 0 h	43512.00±15.93	87024.00±12.15	50.00±0.03
CAP_BPh40_10 t = 24 h	48840.00±24.36	87616.00±20.10	55.74±0.05
CAP_BPh40_20 t = 0 h	41313.00±28.96	85556.00±21.82	48.29±0.06
CAP_BPh40_20 t = 24 h	153.00±15.16	87320.00±24.94	0.18±0.05

Table S2. Effect of neat hydrogels and drug-loaded hydrogels on the viability of HMEC-1 cells assayed immediately after gel administration and after 24 hours of incubation.

	area with cells	total area	% of area with cells relative to total area
control t = 0 h	47040.00±14.04	87024.00±10.90	54.05±0.03
control t = 24 h	62622.00±13.66	86436.00±12.08	72.45±0.03
pure gel PC30 t = 0 h	44900.00±18.17	87024.00±24.66	51.59±0.05
pure gel PC30 t = 24 h	50862.00±22.15	87024.00±24.58	58.45±0.05
CAP_PC30_5 t = 0 h	44688.00±21.96	87024.00±18.15	51.35±0.05
CAP_PC30_5 t = 24 h	33755.00±12.57	87320.00±10.78	38.66±0.03
CAP_PC30_10 t = 0 h	47200.00±11.93	87025.00±9.52	54.24±0.03

CAP_PC30_10 t = 24 h	46278.00±12.75	87024.00±10.96	53.18±0.03
CAP_PC30_20 t = 0 h	46746.00±20.59	86436.00±16.57	54.08±0.04
CAP_PC30_20 t = 24 h	36460.00±28.09	86142.00±26.50	42.33±0.06
pure gel PC42 t = 0 h	43364.00±17.98	85849.00±22.63	50.51±0.04
pure gel PC42 t = 24 h	50808.00±22.98	86140.00±26.21	58.98±0.05
CAP_PC42 5_t = 0 h	47334.00±16.16	87320.00±12.97	54.21±0.03
CAP_PC42_5 t = 24 h	44369.00±19.52	87320.00±15.26	50.81±0.04
CAP_PC42_10 t = 0 h	43806.00±14.41	87024.00±10.95	50.34±0.03
CAP_PC42_10 t = 24 h	54096.00±14.26	87024.00±13.39	62.16±0.03
CAP_PC42_20 t = 0 h	46315.00±23.40	87025.00±18.73	53.22±0.05
CAP_PC42_20 t = 24 h	39273.00±22.35	86730.00±20.40	45.28±0.05
pure gel BE45 t = 0 h	43660.00±17.40	87025.00±29.91	50.17±0.05
pure gel BE45 t = 24 h	48970.00±27.58	87025.00±38.31	56.27±0.07
CAP_BE45 5 t = 0 h	47334.00±13.43	87320.00±10.48	54.21±0.03
CAP_BE45 5 t = 24 h	33774.00±13.65	87025.00±12.28	38.81±0.03
CAP_BE45 10 t = 0 h	43218.00±15.16	87024.00±11.49	49.66±0.03
CAP_BE45 10 t = 24 h	40572.00±12.59	87024.00±10.84	46.62±0.03
CAP_BE45 20 t = 0 h	44840.00±22.66	87025.00±17.69	51.53±0.05
CAP_BE45 20 t = 24 h	28620.00±28.21	87025.00±28.38	32.89±0.07
pure gel BPh40 t = 0 h	43955.00±19.33	87025.00±23.32	50.51±0.05
pure gel BPh40 t = 24 h	50150.00±20.81	87025.00±33.02	57.63±0.06
CAP_BPh40_5 t = 0 h	47495.00±12.52	87320.00±9.79	54.39±0.03
CAP_BPh40_5 t = 24 h	38558.00±14.82	87320.00±11.71	44.16±0.03
CAP_BPh40_10 t = 0 h	45725.00±16.41	87025.00±12.63	52.54±0.03
CAP_BPh40_10 t = 24 h	46905.00±14.20	87025.00±12.35	53.90±0.03
CAP_BPh40_20 t = 0 h	45430.00±24.42	86730.00±19.52	52.38±0.05
CAP_BPh40_20 t = 24 h	46610.00±24.00	86730.00±21.57	53.74±0.05

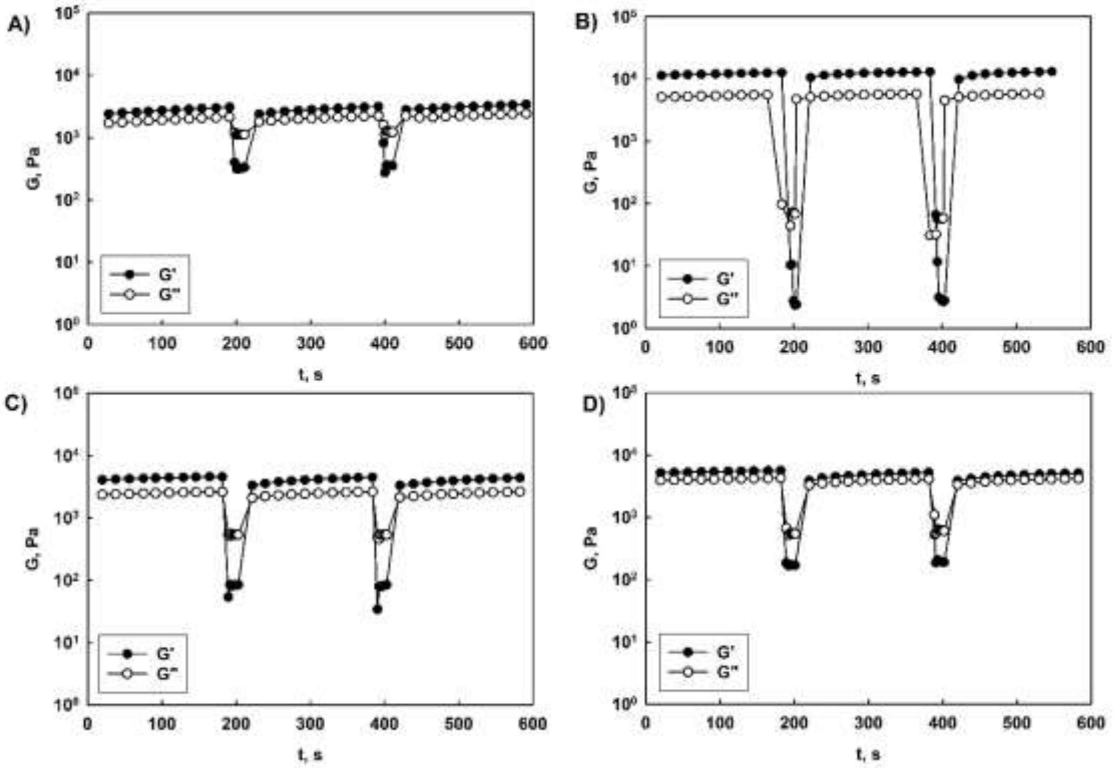

Figure S33. The self-healing tests performed at 37 ºC for hydrogels constructed of PC30 (A), PC42 (B), BE45 (C) and BPh40 (D). The sample of each hydrogel was first placed under a 1% strain for 180 s, and then destroyed with a 5-second pulse of 300% strain, after which 1% was again applied for sample restoration. The hydrogel sample was then destroyed again with 300% strain (5 s) and regenerated to the original values of G' and G” moduli at 1% strain.

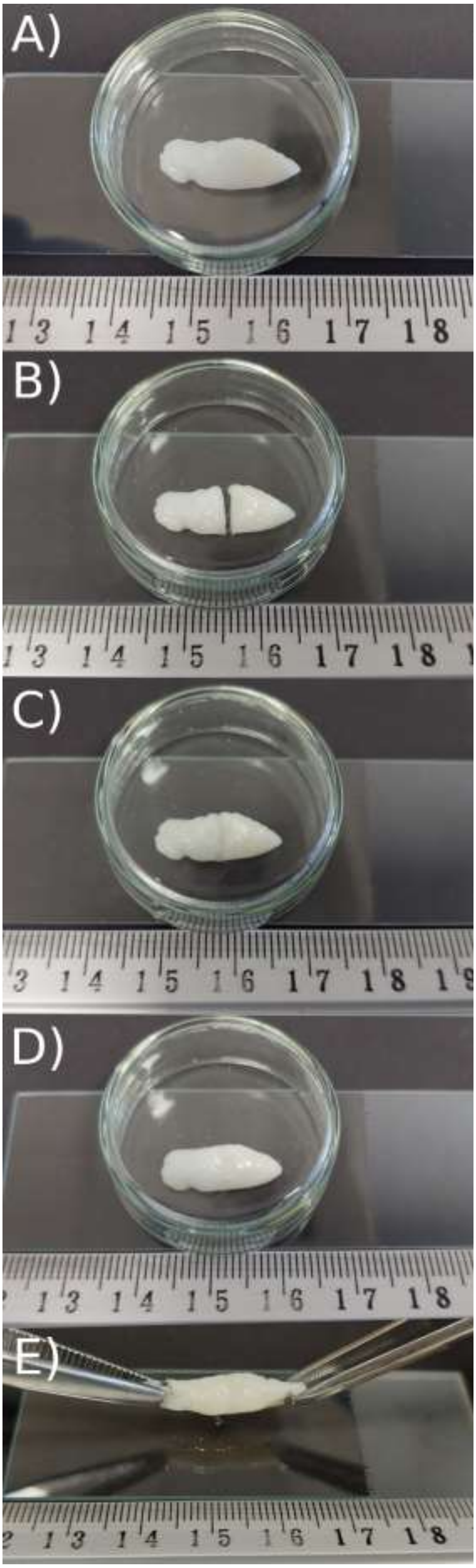
A)
13 14 15 16 17 18
B)
13 14 15 16 17 18
C)
14 15 16 17 18
D)
13 14 15 16 17 18
E)
13 14 15 16 17 18

Figure S34. Illustration of the experiment demonstrating the self-healable behaviour of aryl-enriched HbPGL-based (BE45) hydrogel. The hydrogel sample (A) was ruptured into two pieces (B) and then placed in close contact (C). The sample was gradually repaired after 20 min (D) and then the sample was with a couple of tweezers (E), which revealed that the sample was self-healable under ambient conditions.

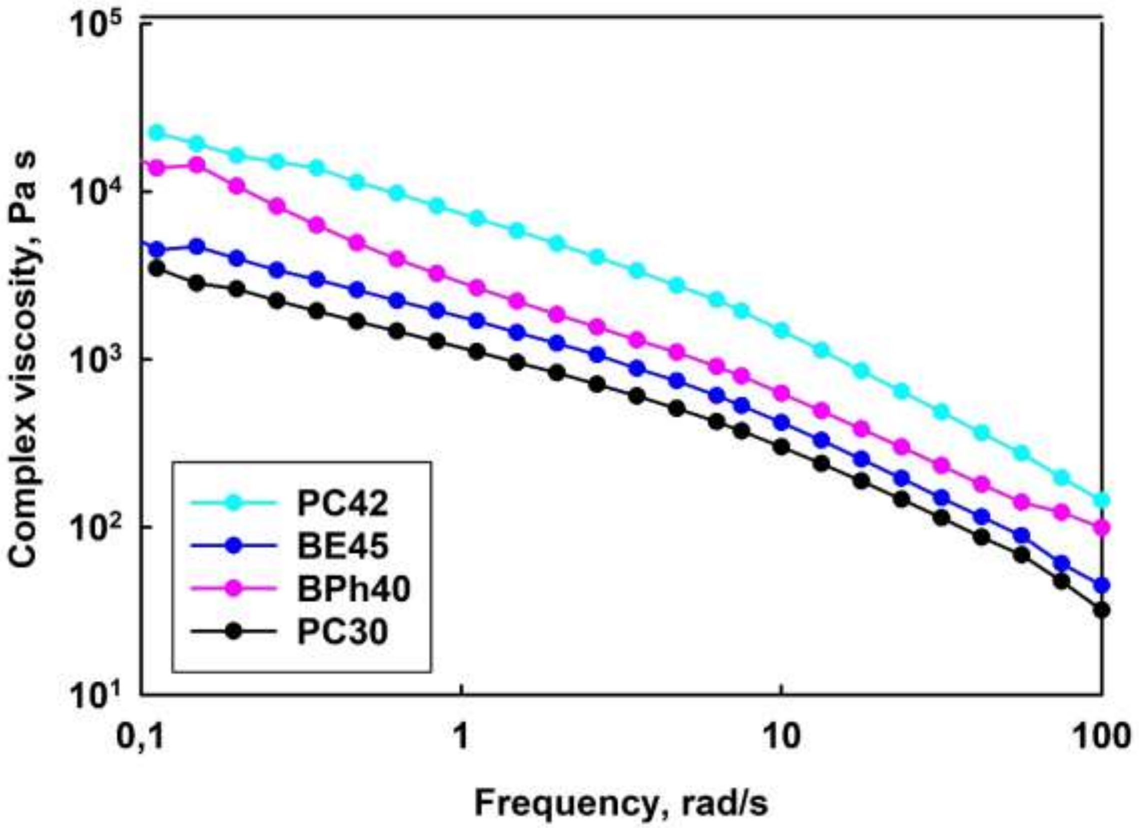

Figure S35. The dependence of the complex viscosity on frequency recorded at 37 °C for drug-loaded hydrogels based on aryl-enriched hyperbranched polyglycidols.

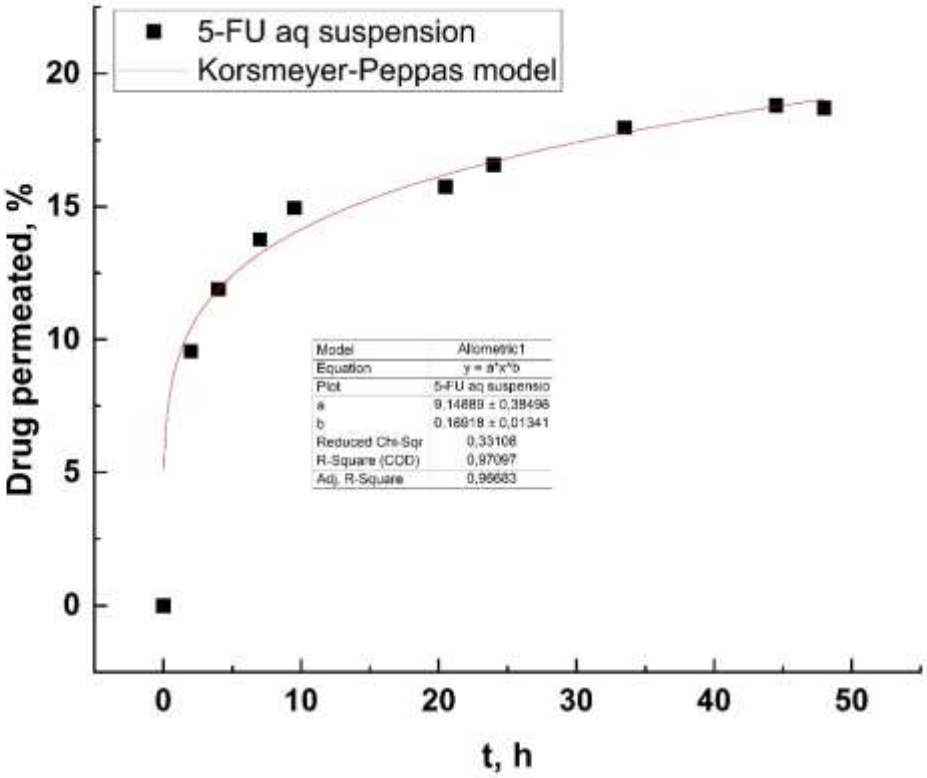

Figure S36. Fitting the release rate data of 5-fluorouracil from the aqueous suspension to a Korsmeyer-Peppas model.

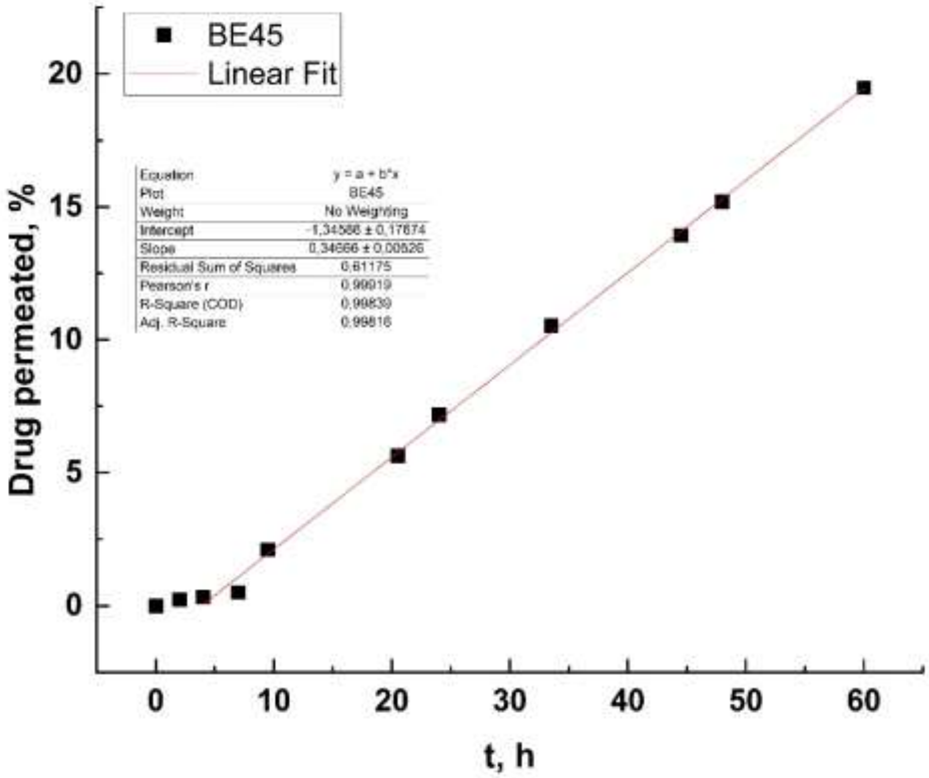

Figure S37. Fitting the release rate data of 5-fluorouracil from BE45-based hydrogel to a zero-order model.

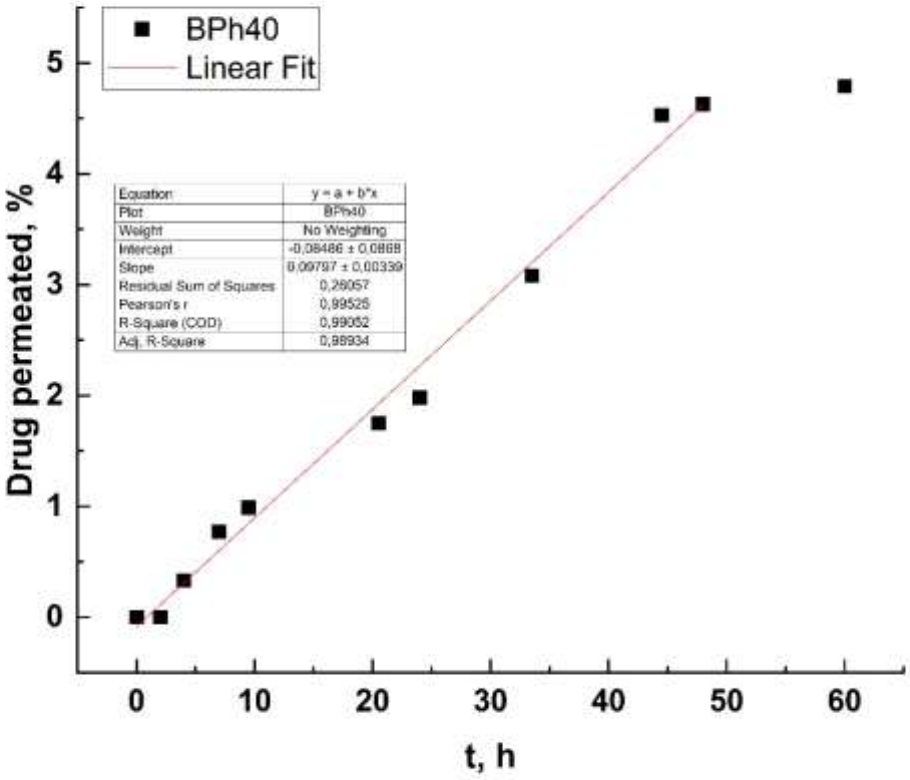

Figure S38. Fitting the release rate data of 5-fluorouracil from BPh40-based hydrogel to a zero-order model.

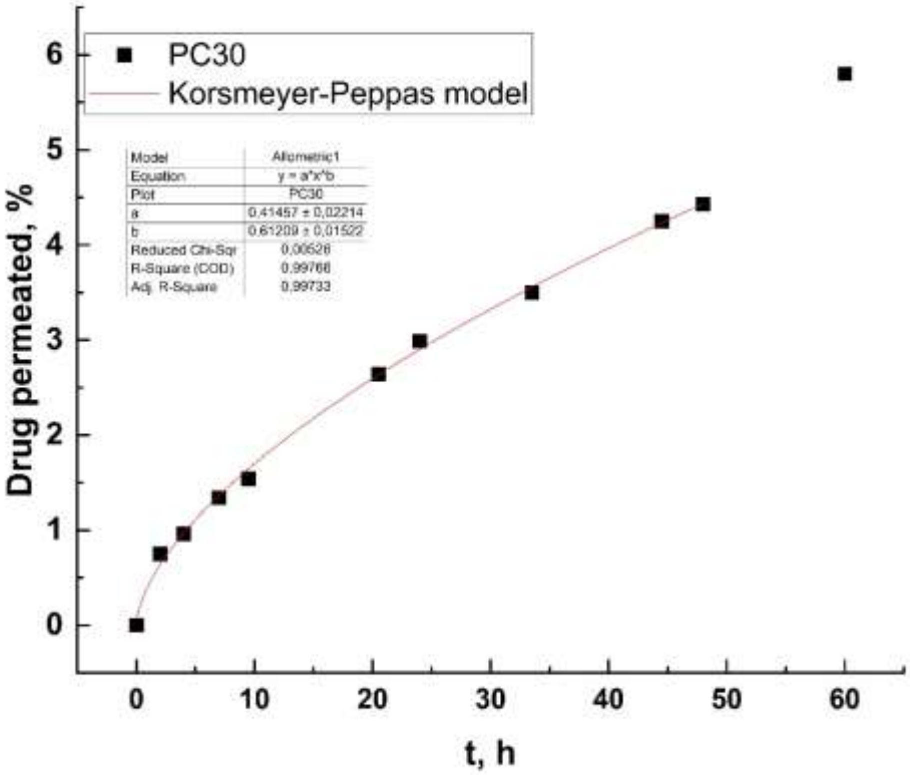

Figure S39. Fitting the release rate data of 5-fluorouracil from PC30-based hydrogel to a Korsmeyer-Peppas model.

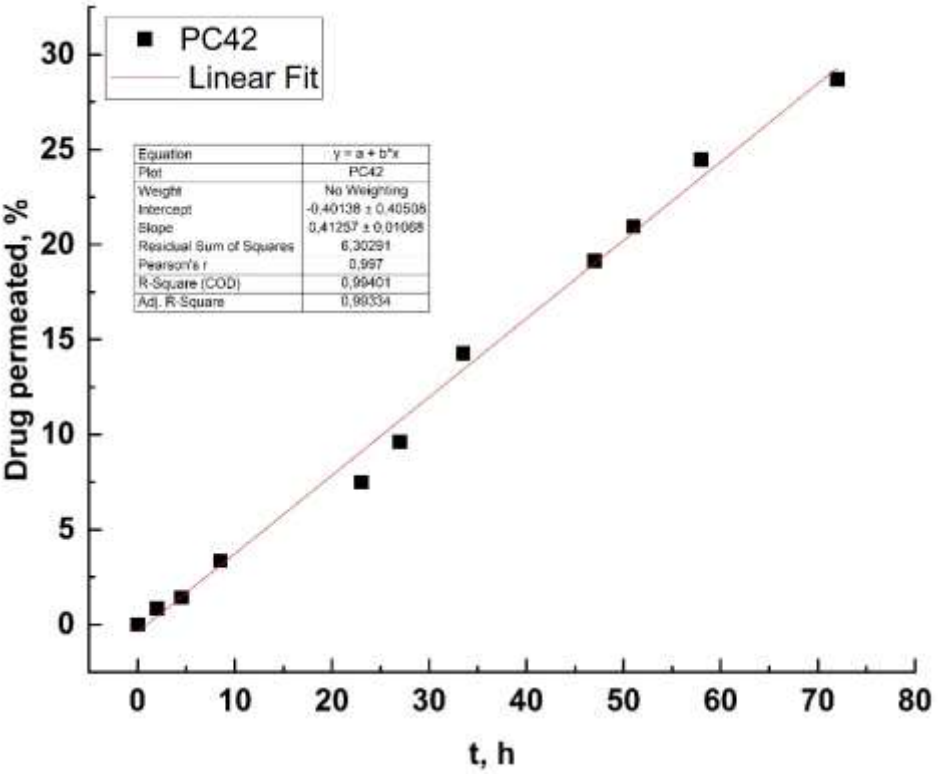

Figure S40. Fitting the release rate data of 5-fluorouracil from PC42-based hydrogel to a zero-order model.

[S1] Schneider CA, Rasband WS, Eliceiri KW. NIH Image to ImageJ: 25 years of image analysis. Nature Methods. 2012; 9(7):671-5. doi: 10.1038/nmeth.2089.
